# Early-Life Adversity Leaves Its Imprint on the Oral Microbiome for More Than 20 Years and Is Associated with Long-Term Immune Changes

**DOI:** 10.3390/ijms222312682

**Published:** 2021-11-24

**Authors:** Eleftheria G. Charalambous, Sophie B. Mériaux, Pauline Guebels, Claude P. Muller, Fleur A. D. Leenen, Martha M. C. Elwenspoek, Ines Thiele, Johannes Hertel, Jonathan D. Turner

**Affiliations:** 1 Immune Endocrine and Epigenetics Research Group, Department of Infection and Immunity, Luxembourg Institute of Health (LIH), 29 Rue Henri Koch, L-4354 Esch-sur-Alzette, Luxembourg; eleftheria.charalambous@lih.lu (E.G.C.); sophie.meriaux@lih.lu (S.B.M.); pauline.guebels@lih.lu (P.G.); claude.muller@lih.lu (C.P.M.); fleur.a.leenen@gmail.com (F.A.D.L.); martha.elwenspoek@bristol.ac.uk (M.M.C.E.); 2 Faculty of Science, Technology and Medicine, University of Luxembourg, 2 Avenue de Université, L-4365 Esch-sur-Alzette, Luxembourg; 3 School of Medicine, National University of Ireland, H91 YR71 Galway, Ireland; ines.thiele@nuigalway.ie (I.T.); Johannes.Hertel@med.uni-greifswald.de (J.H.); 4 Ryan Institute, National University of Galway, H91 TK33 Galway, Ireland; 5 Division of Microbiology, National University of Galway, H91 TK33 Galway, Ireland; 6 APC Microbiome Ireland, T12 HW58 Cork, Ireland; 7 Department of Psychiatry and Psychotherapy, University Medicine Greifswald, 17489 Greifswald, Germany

**Keywords:** early-life adversity, early experience, microbiome, bacterial community, oral microbiome, developmental origins of health and disease, immune system, host-microbe interactions

## Abstract

The early-life microbiome (ELM) interacts with the psychosocial environment, in particular during early-life adversity (ELA), defining life-long health trajectories. The ELM also plays a significant role in the maturation of the immune system. We hypothesised that, in this context, the resilience of the oral microbiomes, despite being composed of diverse and distinct communities, allows them to retain an imprint of the early environment. Using 16S amplicon sequencing on the EpiPath cohort, we demonstrate that ELA leaves an imprint on both the salivary and buccal oral microbiome 24 years after exposure to adversity. Furthermore, the changes in both communities were associated with increased activation, maturation, and senescence of both innate and adaptive immune cells, although the interaction was partly dependent on prior *herpesviridae* exposure and current smoking. Our data suggest the presence of multiple links between ELA, Immunosenescence, and cytotoxicity that occur through long-term changes in the microbiome.

## 1. Introduction

Early-life adversity (ELA) is defined by a poor environment and conditions in early life that induce intense psychophysiological stress [1]. It is mostly observed together with low socioeconomic status and is pathophysiologically correlated with a lifelong imbalance of health and disease [2]. The first 1000 days from conception to 2 years is the most vulnerable life period [3]. At birth, the body is almost fully formed; however, many biological systems continue to mature over the following years. Research on the lifelong health and disease balance has shown the significance of the environment during this period on multiple disease phenotypes [4], including cardiovascular, allergic, and autoimmune disorders, as well as mental disorders [5,6,7,8,9,10,11,12,13,14,15,16,17]. There has been a focus on the molecular mechanisms and the cellular phenotype behind the effect of stress and adversity on immune and endocrine systems as well as epigenetic modifications [5,6,18,19].

ELA has been reported to influence health trajectories via the immune system [18,20,21], with a clear ELA-associated immunophenotype centred around the activation and functional status of T lymphocytes. In the institutionalisation model of early-life stress, strong T-cell immunosenescence has been reported [18,21,22]. Immunosenescence is a form of accelerated immune ageing. The CD57 T- and NK- cell immunosenescence marker is absent in early life and increases with age, with high numbers of such cells in the elderly population. Immunosenescence is driven by chronic inflammation or recurrent viral infections such as CMV [23]. NK functionality is also highly impacted by recurrent reactivation of CMV inducing NK cell exhaustion, increased cytotoxicity, and senescence [24]. Additionally, such viral infections potentially program the immune system [21,22]. Latent CMV infection of haematopoietic progenitor cells reduces GR transcription and translation, impacting immune cell maturation, which can be dependent on CMV reactivation [19,25,26].

The environment is the most critical factor shaping ELA. In the immediate postnatal period, the immune system starts maturing and the first body-area-specific microbial communities are established. Once established, the microbiome modulates the host [27], a mechanism to protect symbiotic microbial communities, where cases of microbial dysbiosis can be fatal [28,29]. The ELM plays important roles in an infant’s subsequent development [5,6] and a long-term health trajectory [5,6,7,8,9,10,11]. Nevertheless, the development of the ELM is critically impacted by the environment, mode of birth, early-life nutrition, and environmental exposure, which leave a clear lifelong trace [16,30]. For example, maternal milk is rich in essential nutrients, protective antibodies, and components essential for the developing microbiome, such as human milk oligosaccharides (HMOs) and short-chain fatty acids (SCFAs) [31,32,33,34,35,36].

The oral microbiome (OM) is composed of various distinct, smaller communities within the oral cavity [37,38,39,40,41] that are robust, stable, and particularly resilient [42,43], particularly to antibiotic therapy [43,44,45,46,47]. Moreover, saliva contains actively secreted components such as cortisol, glucose, lactate, urea, and proteins, such as polypeptides, glycoproteins (cystatins, mucins, and immunoglobulins) and antimicrobial peptides (histatins, defensins, and immunoglobulins-IgA). Many of these are energy sources for the OM, and salivary glycoproteins are the principal nutrient source. These substrates are crucial for the development of multispecies communities and their preservation [41,46,48], and enhance the resistance of the community to environmental stressors [41,49,50]. The long-term stability of the OM leads to the hypothesis that, once established in early life, it remains stable, robust, and resilient, retaining an imprint of the early environment [42,43].

We previously reported higher virally mediated activation and senescence of the immune system in association with ELA in the EpiPath cohort [22,51]. This cohort consists of young adults exposed to ELA by either institutionalisation or separation from their parents at birth, and were subsequently adopted into Luxembourg, while controls were raised by their biological parents. With the growing evidence of a microbiome–immune–system interaction, we attempted to identify if institutionalisation left a mark on the OM of the adoptees. In this study, we sequenced the 16s-rRNA from the buccal and salivary bacterial communities from our cohort. Integrating this with the full immunophenotype, we identified associations with various taxa and analysed how the microbiome interacts with the immune system.

## 2. Results

The V4 region of the bacterial 16S gene was successfully amplified from both buccal swabs and salimetrics oral swabs for the 115 members of the EpiPath cohort, and a total of 45 Gbp sequencing data were obtained. All samples were successfully processed using mothur and a total of 288 and 371 genera from 24 phyla were identified for buccal and salivary samples respectively. The saliva and buccal swabs from the EpiPath cohort were examined independently as they are two distinct oral communities from the same participants.

### 2.1. Microbial Diversity and Overall Microbial Composition

#### 2.1.1. Salivary Microbiome (α- and β-Diversity)

**Figure 1:**
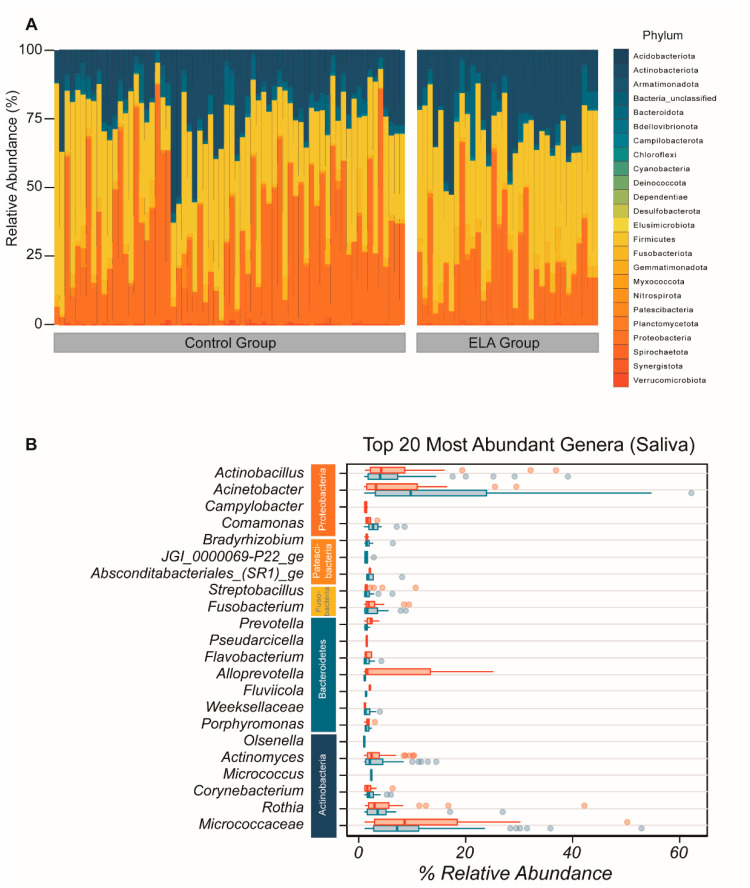
Overall composition of salivary bacterial community. (**A**) Overall microbial composition displayed in stacked area bar plot with the percentage relative abundance of all phyla found in each participant in both study arms. (**B**) Top 20 most abundant genera by mean abundance arranged graphically by phyla. Vertical line = mean; rectangle = 1st to 3rd quartile; horizontal lines = 2.5th to 97.5th percentile. Outliers are indicated as individual data points. Blue, control group; red, ELA group.

We identified sequences from all of the 24 principal bacterial phyla in the overall salivary microbial community. Within these 24 phyla, the most abundant in both control and ELA groups were *Actinobacteria*, *Proteobacteria*, *Firmicutes*, and *Fusobacteriota* (Figure 1A). The most abundant genera of the salivary composition were *Acinetobacter*, *Micrococcaceae*, *Actinobacillus*, *Rothia*, *Corynebacterium*, *Micrococcus*, *Actinomyces*, *Alloprevotela*, *Porphymonas*, *Fusobacterium*, *Weeksellaceae*, *Flavobacterium*, *Bradyrhizobium*, *Porphyromonas*, *_ Comamonas*, *Olsenella*, *Fluviicola*, *Fusobacterium*, *Absconditabacteriales _(SR1)_ge*, *Streptobacillus*, *Fretibacterium*, *JGI_0000069-P22_ge*, *Capnocytophaga*, *Pseudarcicella*, *Tannerella*, *Prevotella*, and *Campylobacter* (Figure 1B). There was no difference in α-diversity between the controls and the adoptees in terms of diversity (controls: mean = 13.87872, SD = 7.127721, adoptees: mean = 14.14869, SD = 6.601899, Wilcoxon rank sum test *p* = 0.7579) and evenness (controls: mean = 0.5619667, SD = 0.06864806, adoptees: mean = 0.5646776, SD = 0.06172601, Wilcoxon rank sum test *p* = 0.9461). Plotting the Shannon evenness index against the inverse Simpson diversity index confirmed that there analogous diversity and evenness between the controls and adoptees (Supplementary Figure S1). Principal coordinate analysis could not detect systematic differences either (Supplementary Figure S1).

#### 2.1.2. Buccal Microbiome (α- and β-Diversity)

**Figure 2:**
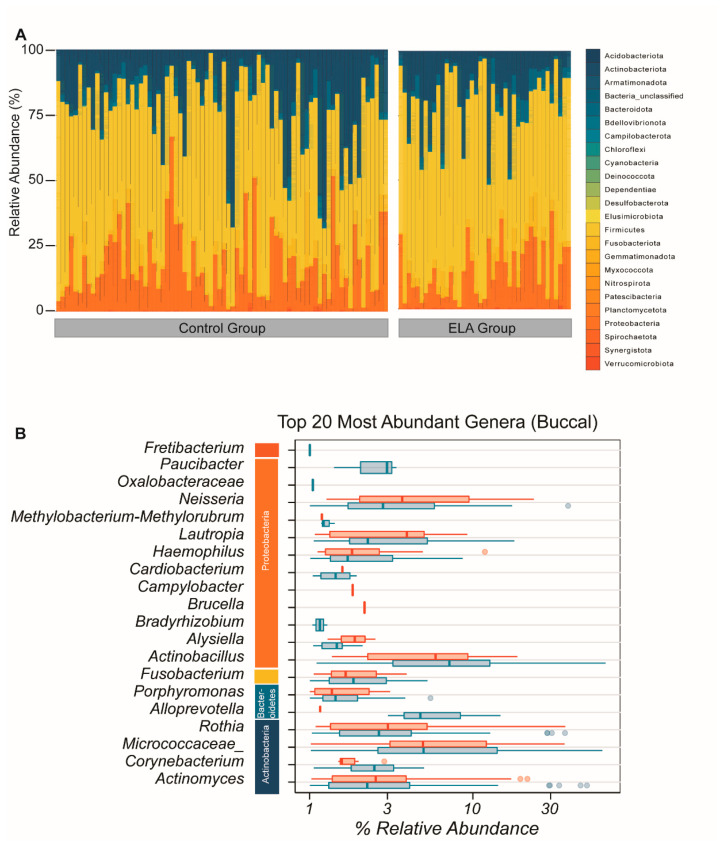
Overall composition of the buccal bacterial community. (**A**) Overall microbial composition displayed in stacked area bar plot with the percentage relative abundance of all phyla found for each participant in both study arms. (**B**) Top 20 most abundant genera by mean abundance arranged graphically by phyla. Vertical line = mean; rectangle = 1st to 3rd quartile; horizontal lines = 2.5th to 97.5the percentile. Outliers are indicated as individual data points. Blue, control group; red, ELA group.

As for the salivary microbiome, we identified sequences from all the 24 principal bacterial phyla in the buccal microbial community. The most abundant phyla were the same as in the salivary microbiome: *Actinobacteria*, *Proteobacteria*, *Firmicutes*, and *Fusobacteriota* (Figure 2A). The most abundant genera were *Actinomyces*, *Corynebacterium*, *Micrococcaceae*, *Rothia*, *Alloprevotela*, *Porphymonas*, *Fusobacterium*, *Actinobacillus*, *Bradyrhizobium*, *Haemophilus*, *Methylobacterium-Methylorubrum*, *Oxalobacteracea*, *Actinomyces*, *Neisseria*, *Paucibacter*, *Lautropia Cardiobacterium*, *Brucella*, *Alysiella*, and *Campylobacter*, therefore revealing a substantial overlap in the detected genera between the saliva and the buccal microbiome (Figure 2B).

As for the salivary microbiome, we observed a very similar diversity and evenness between controls and adoptees as measured by the inverse Simpson index (controls: mean = 12.85828, SD = 10.117292, adoptees: mean = 14.72315, SD = 8.991065, Wilcoxon rank sum test *p* = 0.08803). The Shannon evenness index was similar between adoptees and controls, and, in both, it was higher than in the salivary microbiome (controls: mean = 0.5137724, SD = 0.10141122, adoptees: mean = 0.465861, SD = 0.08482336, Wilcoxon rank sum test *p* = 0.09024). α-diversity was again similar in the controls and the adoptees in terms of evenness (Wilcoxon rank sum test *p* = 0.08803) and diversity (Wilcoxon rank sum test *p* = 0.09024). Plotting the Shannon evenness index against the inverse Simpson diversity index revealed no systematic differences in diversity or evenness between the controls and adoptees (Supplementary Figure S1). Similarly, principal coordinate analysis indicated no differences between adoptees and control (Supplementary Figure S1).

#### 2.1.3. Salivary and Buccal Microbiomes Are Two Separate Entities

**Figure 3:**
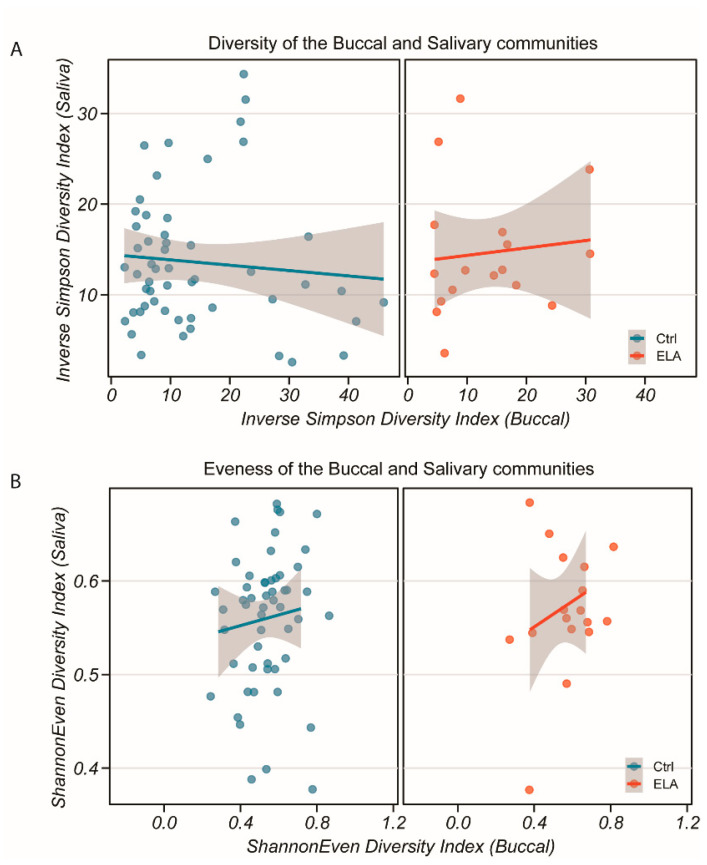
Diversity and evenness of the salivary and buccal bacterial communities in both study groups. (**A**) Inverse Simpson diversity index of saliva against buccal communities for control (**left**) and ELA (**right**). (**B**) Shannon evenness index of saliva against buccal communities for control (**left**) and ELA (**right**). No correlation was found between either community for either measure or group (Spearman’s rank correlation test, *p* > 0.47). Grey shaded area: 95% confidence interval.

To ensure that sample collection was performed correctly and that we had two distinct communities, we compared the diversity and evenness of the salivary and buccal samples. We found a low correlation between the salivary and buccal communities in both the inverse Simpson diversity index (Figure 3A, *p* = 0.47, rho = −0.07372058, Spearman’s rank correlation test) and Shannon evenness index (Figure 3B, *p* = 0.8759, rho = 0.01595802, Spearman’s rank correlation test), giving evidence in favour of the hypothesis that, despite their close physical proximity, they can be seen as distinct communities. Comparing the salivary and buccal microbiomes by group, the diversity ratios of the salivary against buccal communities were similar between the controls and adoptees (Figure 3A, controls: *p* = 0.3311, rho = −0.1222465; adoptees: *p* = 0.7898, rho = 0.04812834; Spearman’s rank correlation test, Figure 3B; controls: *p* = 0.9578, rho = 0.009692513; adoptees: *p* = 0.7898, rho = −0.04812834; Spearman’s rank correlation test). This suggests that the overall composition between the controls and adoptees may be similar, but differences would be seen at the phyla level.

### 2.2. ELA Induces Differences in Specific Taxa in Both Salivary and Buccal Communities

**Figure 4:**
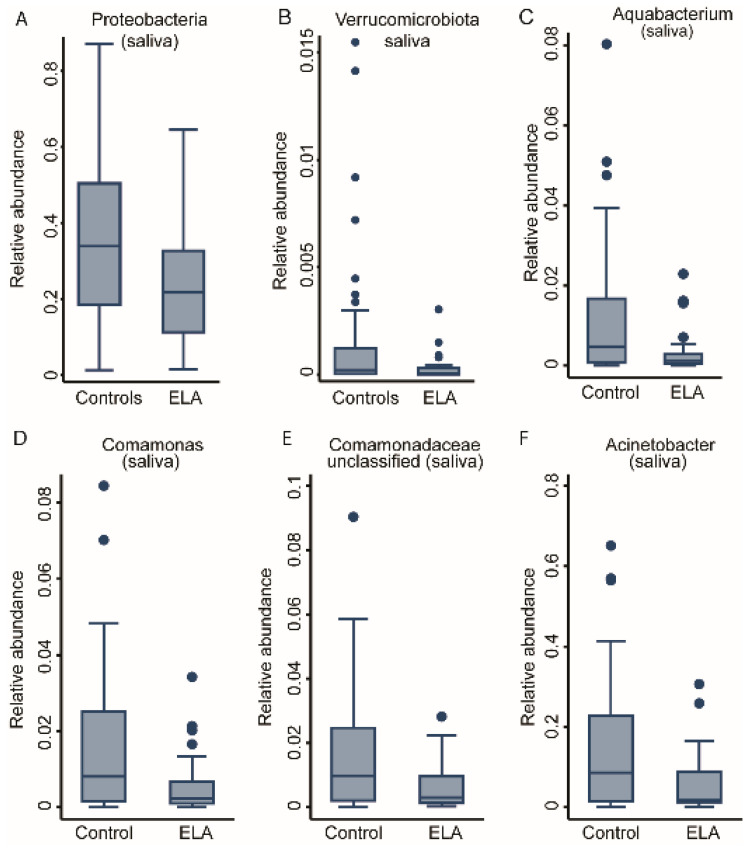
Taxonomic differences between study groups in the salivary bacterial community. Box plots of two phyla, (**A**) Proteobacteria and (**B**) Verruomicrobiota, as well as four genera: (**C**) Aquabacterium, (**D**) Comamonas, (**E**) Comamonadaceae, unclassified and (**F**) Acinetobacter. All are significantly associated with study group; fractional regressions against study group were calculated to determine significance (FDR < 0.05). Horizontal line = mean; rectangle = 1st to 3rd quartile; vertical lines = 2.5th to 97.5th percentile. Outliers are indicated as individual data points.

Investigating the abundance levels of phyla and genera highlighted differences in the community composition across the ELA group and healthy controls (Table 1). While there were no differences in the phyla level in the buccal data, *Proteobacteria* and *Verrucomicrobiota* were significantly lower (FDR < 0.05 for both) in the adoptees in comparison to the controls (Supplementary Table S1A, Figure 4A,B) in the saliva microbiome as detected in fractional regression analyses. Analyses of deeper taxonomy revealed two of the most abundant genera of the *Proteobacteria* phylum, *Comamonas* and *Acinetobacter*, to be significantly lower in the saliva of adoptees compared to controls alongside *Aquabacterium* and unclassified *Comamonadaceae* (Table S1B, Figure 4C–F). In conclusion, while we could not detect systematic differences in the buccal microbiome between the ELA group and the controls, the saliva microbiome was structurally different in its composition, with a prominent role for *Proteobacteria* genera (Figure 4A).

### 2.3. Environmental Covariates

Next, we investigated the impact of environmental factors on the OM to potentially explain the effects of ELA described above (Table 1).

#### 2.3.1. Smoking

**Figure 5:**
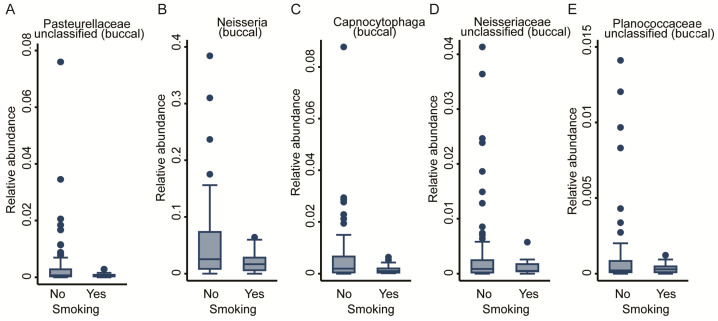
Taxonomic differences in the buccal bacterial community associated with smoking. Box plots of five genera: (**A**) Pasteurellaceae unclassified, (**B**) Neisseria, (**C**) Capnocytophaga, (**D**) Neisseriaceae unclassified, and (**E**) Planococcaceae unclassified. All are significantly associated with study group; fractional regressions against smoking were calculated to determine significance (FDR < 0.05). Horizontal line = mean; rectangle = 1st to 3rd quartile; vertical lines = 2.5th to 97.5th percentile. Outliers are indicated as individual data points.

As lifestyle has a pivotal role in the development of the microbiome, we assessed the effect of smoking on the OM by including smoking status (binary: smokers vs. non-smokers) into the regression modelling (Table 1). No significant genera were detected for the salivary community, whereas from the buccal community, we exposed five genera negatively associated with smoking: three from the *Proteobacteria* phylum, *Neisseria*, *Neisseriaceae_unclassified*, and *Pasteurellaceae_unclassified*; 1 from the *Bacteroidetes* phylum and *Capnocytophaga* genus; as well as one from the *Firmicutes* phylum and *Planococcaceae_unclassified* genus (FDR < 0.05, Figure 5A–E). In sensitivity analyses, we removed smoking as a covariate from the regression equations for the FDR-corrected significant genera to explore potential effect mediation through smoking, but the results remained virtually unchanged. The full results for the buccal and saliva microbiomes can be found in Supplementary Table S2.

#### 2.3.2. Prior Viral Infections

**Figure 6:**
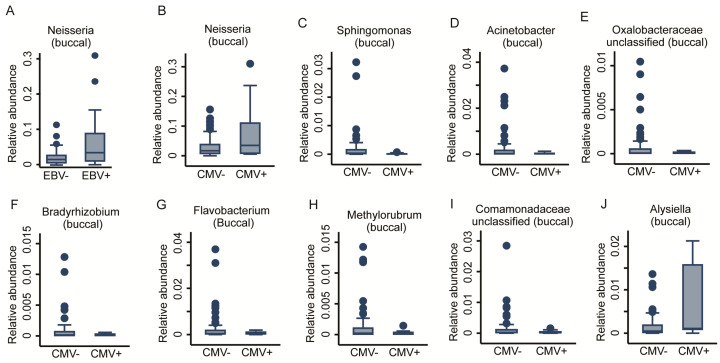
Taxonomic differences in the buccal bacterial community associated with anti-herpesviridiae serological status. Box plots of one genus (**A**) *Neisseria* significantly associated with anti-EBV antibody titres. Nine genera, (**B**) *Neisseria*, (**C**) *Sphingomonas*, (**D**) *Acinetobacter*, (**E**) *Oxalobacteraceae unclassified*, (**F**) *Bradyrhizobium*, (**G**) *Flavobacterium*, (**H**) *Methylorubrum*, (**I**) *Comamonadeceae unclassified*, and (**J**) *Alysiella*, were significantly associated with anti-CMV antibody titres. Fractional regressions against the presence of anti-EBV and anti-CMV antibodies were calculated to determine significance (FDR < 0.05). Horizontal line = mean; rectangle = 1st to 3rd quartile; vertical lines = 2.5th to 97.5th percentile. Outliers are indicated as individual data points.

**Figure 7:**
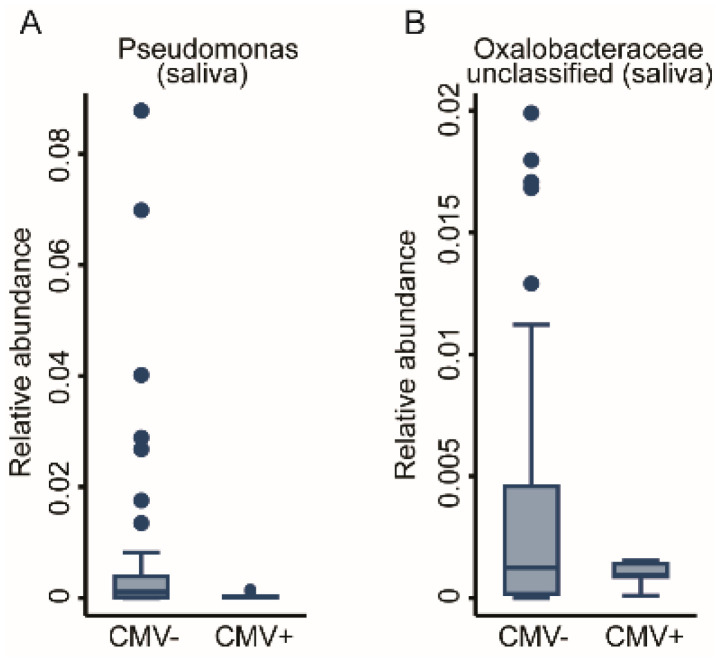
Taxonomic differences in the salivary bacterial community associated with anti-CMV serological status. Box plots of two genera, (**A**) *Pseudomonas* and (**B**) *Oxalobacteraceae unclassified*, which were significantly associated with CMV antibody titres. Fractional regressions against the presence of anti-CMV antibodies were calculated to determined significance (FDR < 0.05). Horizontal line = mean; rectangle = 1st to 3rd quartile; vertical lines = 2.5th to 97.5th percentile. Outliers are indicated as individual data points.

We previously reported that viral infections may mediate the early-life immunophenotype [22]. Consequently, we tested whether prior viral infection, measured as anti- HSV, EBV, and CMV seropositivity, affected the oral bacterial communities. We achieved this via fractional regressions with the antibody titre (binary: positive vs. negative) as the predictor of interest and the genus abundance as the response variable (Table 1). Due to low case numbers of positive titre results for CMV, we could not adjust for basic covariates without inducing numerical instability in the fitting procedure. While HSV titres did not show any association, a positive EBV titre was positively associated with the abundance of the *Neisseria* genus in the buccal microbiome (FDR < 0.05, Figure 6A). However, anti-CMV antibody titres showed a very strong association with the OM. In total, 10 genera had a significant association with CMV titres. Nine genera from the Buccal community, eight derived from the *Proteobacteria* phylum (*Acinetobacter*, *Bradyrhizobium*, *Comamonadaceae_unclassified*, *Methylobacterium-Methylorubrum*, *Oxalobacteraceae_unclassified*, and *Sphingomonas* genera) unveiled a negative association, whereas the genera of *Alysiella* and *Neisseria* demonstrated a positive association. One genus from *Bacteroidetes* phylum, *Flavobacterium*, also appeared to be negatively associated with positive CMV titres (FDR < 0.05, Figure 6B–J). Two genera of the Salivary community from the *Proteobacteria* phylum, Pseudomonas and *Oxalobaceraceae_unclassified*, exhibited a negative association (FDR < 0.05, Figure 7A,B). The full results for the buccal and the saliva microbiome antibody titre associations can be found in Supplementary Table S3. In a further step of sensitivity analysis, we included a positive antibody titre as a covariate into the regression models to investigate the differences in the genus abundances between ELA and controls. However, the results virtually remained the same, indicating either insufficient statistical power to detect potential mediation or that CMV exposure does not mediate ELA-related changes in the OM. This suggests that, unlike increased immunosenescence, the changes we saw in the oral bacterial community are independent of prior exposure to Herpesviridae.

### 2.4. Fractional Regression Models of the Immune–Microbiome Interactions

In the next step, we fitted a series of fractional regression models integrating the relative abundance of the taxonomic levels in the salivary and buccal compositions with our previously published immune-system profiling. Among the full dataset of 48 immune cell populations, we identified 11 significant associations with genera, most importantly for T cells and NK cells (Table 1).

#### 2.4.1. Association with CD4 T-Cell Immunosenescence

**Figure 8:**
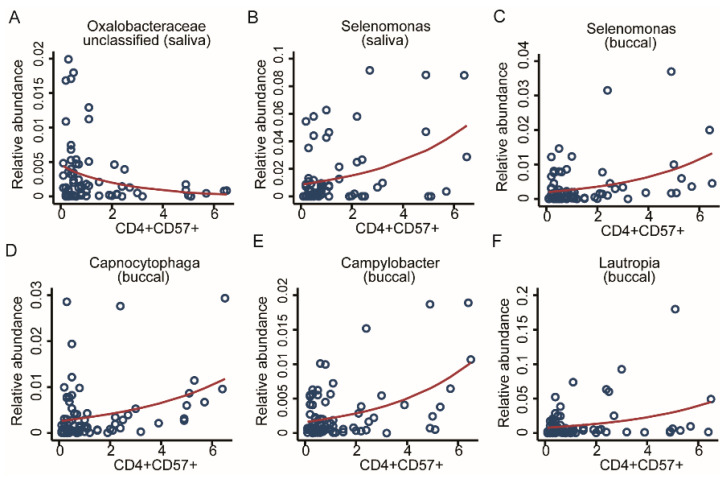
Taxonomic associations in both communities with immunosenescence. Scatter plots with regression lines for six genera. From the salivary community, two genera, (**A**) *Oxalobacteraceae unclassified* and (**B**) *Selenomonas*, as well as four genera from the buccal community, (**C**) *Selenomonas*, (**D**) *Capnocytophaga*, (**E**) *Campylobacter*, and (**F**) *Lautropia*, were all significantly associated with CD4 CD57 cell counts. Fractional regressions against CD4 CD57 cell counts were calculated to determine significance (FDR < 0.05). Regression lines were derived from fractional regressions with logistic parametrisation of the conditional mean.

Immunosenescence is a common result of adversity. Thus, we decided to look for possible associations between adversity, microbiome, and accelerated ageing of immune cells (Table 1). For screening the OM associations with the share of CD57 -positive CD4 and CD8 cells, we used multivariable fractional regressions including the genus abundance as the response variable, and the share of CD57-positive CD4 and CD8 cells as a predictor of interest and the basic set of covariates. Additionally, we included the study-group variable as a covariate to control for potential confounding factors related to ELA status. CD8 T cells were previously reported to be significantly associated with CMV [22], but we found no associated taxonomic markers from the OM. From CD4 T-cells tests, we identified six strong taxonomic associations. Two genera from the salivary microbiome, *Selenomonas* from the *Firmicutes* phylum showed a positive association and *Oxalobacteraceae_unclassified* from the *Proteobacteria* phylum showed a negative association. Four genera from the buccal community: *Selenomonas* from *Firmicutes*, *Capnocytophaga* from the *Bacteroidetes* phylum, and *Campylobacter* and *Lautropia* from the *Proteobacteria* phylum, displayed a positive association (FDR < 0.05, Figure 8A–F). For further exploration, we fit additional fractional regressions using the number of T-helper cells and T-killer cells as predictors of interest using the same set of covariates as before, finding no additionally significant associations after correction for multiple testing. Summary statistics for the buccal and saliva microbiomes are given in Supplementary Table S4.

#### 2.4.2. Association with NK Cell Activity

**Figure 9:**
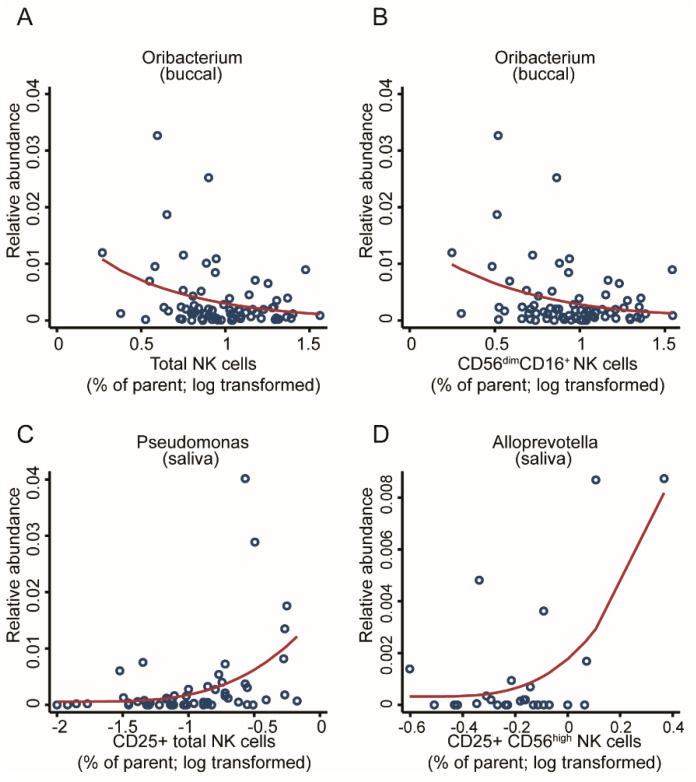
Taxonomic associations in both communities with natural killer cell activity. Scatter plots with regression lines for one genus from the buccal community, *Oribacterium*, was significantly associated with (**A**) the total number of NK cells and (**B**) the total number of mid-maturation NK cells as well as two genera from the salivary community: (**C**) *Pseudomonas*, associated with CD25^+^ NK cell counts; and (**D**) *Alloprevotella*, associated with CD25CD56^hi^ NK cell counts. Fractional regressions against NK cell counts were calculated to determine significance (FDR < 0.05). Regression lines were derived from fractional regressions with logistic parametrisation of the conditional mean.

Innate immune cells such as natural killer (NK) cells are the first line of defence and often interact with commensal bacteria. Adoptees of this cohort showed increased cytotoxicity on their NK cells [52]; hence, we thought to assess for a potential link with the microbiome. Through screening the OM for associations with various types of NK cells, we found three genera associated with cell counts with an FDR < 0.05, while seven additional associations reached an FDR < 0.1 (Supplementary Table S4), hinting that a better-powered study may find a broader association pattern. In the buccal community, the *Oribacterium* genus showed a negative association with the total number of NK cells and the total number of mid-maturation NK cells (FDR < 0.05, Figure 9A,B). In parallel, within the salivary community, several genera were significantly associated with different stages of NK maturation. *Pseudomonas* was found to be positively associated with the total number of CD25 expressing NK cells, which reflects an association with the global activation of NK cells (FDR < 0.05, Figure 9C). The abundance of *Alloprevotella* was positively associated with the abundance of activated immature CD25CD56^hi^ expressing NK cells (FDR < 0.05, Figure 9D).

Summary statistics for the buccal and saliva microbiome are given in Supplementary Table S4 and bacterial taxa are highlighted in Table 1.

## 3. Discussion

In this study, we identified taxonomic differences in the OM 24 years after adversity that were common throughout a cohort of diverse cultural and ethnic origins. We identified genera that had a significantly reduced abundance in the adoptees, which were significantly associated with smoking; immunosenescence of CD4 T cells; circulating number and activation status of NK cells; and anti-CMV and, to a lesser degree, anti-EBV titres. Importantly, we were able to see these differences in both the salivary and buccal microbiomes, both of which are readily accessible and both are regularly and easily sampled, even if the buccal microbiome is somewhat underexplored to date. Our data highlight the distinctness of the salivary and buccal microbiomes in distinct oral niches with unique microbial signatures.

Our findings from the EpiPath cohort closely mirror those of Reid et al. [53], although in significantly different microbial communities. We report differences in the abundance of taxa associated with early institutionalisation and CMV seropositivity. Considering that the gut microbiome (GM) is far more labile to lifestyle and environmental impact than the OM [53], our findings build upon those of Ried et al., opening the possibility of much longer-term studies, as the enhanced stability of the OM suggests that differences may be stable over many decades [53]. Expanding our analyses to associations with the immunosenescent CD4 T cells and the activation status of circulating NK cells strengthens the possible role of microbe–immune cross-talk in ELA and the potentially detrimental outcomes. Furthermore, at the family taxonomic level, we observed highly similar differences to those reported by Reid et al. (e.g., *Prevotella* vs. *Alloprevotella*, both from the *Prevotellaceae* family). This highlights the link between the oral and GMs, as numerous studies provide evidence of bacteria migrating from the oral cavity and colonising the gut, whereas there is no evidence of the opposite happening [54,55,56,57].

Our current findings show that institutionalised, genetically unrelated individuals share particular taxa, identifiable 24 years later, independent of the event of adoption. The buccal community, in contrast to the salivary community, appears to be more prone to lifestyle habits such as smoking, agreeing with previous reports that the salivary community remains stable despite lifestyle-hygiene-related mediations such as flossing [42,43,58]. This agrees with several prior reports of the stability and resilience of oral communities over time [39,41,43,46,47,59,60,61,62]. Although host genetics help to shape microbial communities, previous reports of low variance between twins suggest that the shared early environment is the key determinant of the long-term composition [43,60,62]. Longitudinal observations of twins revealed that the salivary microbiome has a stable core community at the genus level, and as twin lives diverge over time, environmental differences increase the diversity between the microbiomes of twins [60,61]. Furthermore, genetically unrelated people with a shared environment show similar environment-related effects on microbiome composition in the mouth as well as other communities [43,47,61]. Cohabitation appears to have a greater impact on the skin microbiome rather than gut and oral communities, persisting after the cohabitation is terminated [43,58,60,63], an effect that is thought to persist for the long term despite leaving or changing household [41,48,49].

The importance of the OM should not be underestimated. As for the GM, there is a direct interaction between the microbiome and both oral and systemic health. Multiple oral inflammatory microbiome-associated conditions such as periodontitis and carries have strong epidemiological and mechanistic associations to other systemic and gastrointestinal diseases [61,64]. Further associations over the years have identified oral marker links to systemic complications, including cardiovascular, immune, metabolic, respiratory, osteopathic, obstetric, and perinatal complications [64,65,66]. In both healthy and inflammatory statuses, viable oral bacteria are often found to travel from the mouth to the gut and are capable of achieving successful colonisation [54,55,56]. Schmidt et al. found that more than half of identified species often found residing in both mouth and gut exhibited signs of oral–gut transmission for all their study participants. Nearly one-third of these are taxa known to be highly dominant in oral communities [54,57,67]. Interestingly, this is a one-way observation: although oral strains can travel to and colonise the gut, the opposite is unlikely to occur [54,57]. Hence, as dental health research has been suggesting for years, oral microbial composition hinges on oral and dental health. In contrast to the prevailing GM, OM shows rising importance as an indicator of systemic health.

Although observational, we report numerous clear associations and correlations in our statistical model that demonstrate the crosstalk between the microbiome and the immune system. Microbial transmission across the gastrointestinal tract, direct microbial contact with tissue-resident innate cells, probable oral bacterial infection, circulating bacterial toxins, and molecular mimicry are all valid candidate pathways that may explain the observed relationship [65,66]. The ELM plays a crucial role in educating immune cells (immune tolerance) that are completely naïve at birth. As immune cells learn to recognise host cells, they are also programmed to recognise antigens from the developing beneficial endogenous microbiome [68]. Tissue-resident dendritic cells harvest microbial antigens from local microbial communities and present them to other immune cells [68]. In germ-free mice, the absence of a microbiome during the early-life period alters immune functions and induces structural defects in lymphoid tissues. In the presence of microbial communities, these tissue structures form normally. Despite many such observations, it is unclear how this acts mechanistically to alter the formation of epithelial barriers. Evidence from the gut suggests that bacteria can direct the glycosylation of luminally exposed surface proteins, a process whose outcome differs in germ-free mice [69,70,71]. Initially, T_h_17 cells are absent in germ-free mice and only appear upon microbial colonisation [68,69].

It is now well-established that the relationship between stress and chronic disease starts in utero, as susceptibility and occurrence of disease can be predefined by maternal stress [72]. During this period, the naïve, uneducated, immune system develops [73]. NK cells are part of the body’s first line of immune defence, interacting with other immune cells as well as pathogens. In the majority of chronic diseases associated with the early-life environment, NK cells appear to either have an impaired function or an exaggerated cytotoxic activity [74,75]. The most studied NK cell populations are the CD56brightCD16^−^ and CD56dimCD16bright cells and the associated cytotoxic CD56dim and cytokine-producing CD56bright cells [76]. NK cell cytotoxicity is initiated by target cell contact and recognition, which leads to immune synapse formation, resulting in NK-cell-induced target-cell death. The proliferation and expansion of NK cells depend on CD4^+^ T_h_1 cells. Nevertheless, due to the bidirectional relationship between innate and adaptive immunity, NK cells impact CD4^+^ and CD8^+^ T cells through cytokine production [77]. In the absence of short-chain fatty acids (SCFAs), metabolites produced from fibre fermentation by the local microbiome communities, certain CD4 T-cell subsets do not differentiate. Furthermore, naïve CD8 T cells do not differentiate into memory cells in germ-free mice [68,78,79]. The activation of NK cells by pathogen-associated molecular patterns (PAMPs) may initiate an unwanted response in the microbiome and lead to a strong inflammatory response [80]. Similarly, pathogen-driven activation of NK cells can result in increased on-site cytotoxicity, which can also be harmful to local microbial communities. Correspondingly, microbiome members regulate homeostasis by inducing NK cell expansion and cytokine production or driving the proliferation of anti-inflammatory cytokine-producing NK cells, a common event observed with tissue-resident cells and microbiome crosstalk [81]. Furthermore, immunomodulatory properties of the bacterial community may drive antiviral defences regulating the outcome of viral infection [82].

The OM is intimately linked to oral health. Poor oral health is often approached in an eco-social framework, as it is known to be associated with psychosocial adversity [83]. Both epigenetic and behavioural pathways were linked to poor oral health [83]. One of the most studied causal routes is diet. Affordability and access to a nourishing diet” are strongly influenced by socio-economic status [83], which in turn is linked to the composition of the OM. Detrimental shifts in the microbial composition associated with poor immune responses and mental health were documented for both hospitalised and long-term care home residents [84]. The multidirectional interconnected relationship between the microbial composition, the host’s immunological status, and the resulting life-long health trajectory is most probably highly dependent on constant exposure to particular irritants [84].

Our observation that psychosocial adversity is associated with changes in the OM opens many possibilities for future research. The collection of oral samples, primarily saliva, has been the sampling media of choice for psychobiology, lifestyle, and other social to clinical research areas for many decades. Saliva has long been recognised as an accurate, noninvasive, and cost-effective diagnostic approach that can be tailored to personalised medicine strategies [65,85]. Here, we opened up the possibility of using standard salivary swabs previously collected for microbiome studies. Such studies have the potential to provide a more holistic view of host–microbe interactions and the role of the microbiome in health, which is a potential that can now be applied in nearly all areas of psychobiology (and further afield). Our data also provide preliminary mechanistic insights and the perspectives for future detailed mechanistic studies. We know that early oral microbial colonisation is associated with IL-17-producing cells [86], and subsequent chronic oral disease is often initiated by T_h_17 cells and IL-17 [87,88,89]. In our EpiPath cohort, there was a strong ELA-associated increase in immunosenescence-associated chronic inflammation, together with increased T_h_17 cell numbers, although this narrowly missed significance (*p* = 0.06, [51]). The ELA-associated immunophenotype is centred on immunosenescence [22,51]. Here, we saw clear associations between *Selenomonas*, *Campylobacter*, and *Capnocytophaga* with T-cell immunosenescence, and together with the activated immature NK cell-associated *Alloprevotella*, these genera were all associated with periodontitis, gingivitis, and T2D. Diseases such as periodontitis an gingivitis have long been associated with changes in both the local and peripheral immune systems. This may be mediated by IL-17 from T_h_17 cells, and it has been implicated in periodontitis-associated distal diseases in many disease contexts [90,91]. This is directly induced by microbial dysbiosis [92]. Furthermore, direct microbial interaction with immune cells may underlie this, as loss of Toll-like receptor-2 (TLR2) in antigen-presenting cells reduces IL-17 secretion from T_h_17 cells that dysregulate the host immune system in periodontitis [93]. A similar direct link from the microbiome to the induction of a T_h_17 cell response was previously reported for Streptococcus [94]. As such, it is interesting to hypothesise that innate immune signalling from TLRs on immune cells within the oral cavity may directly mediate microbiome–immune interactions, acting locally and distally.

As with all investigations, our study is not without limitations. Due to the limited quantity of the biobanked samples, 16S sequencing was favoured over shotgun sequencing to ensure good-quality data, leading to a limited taxonomic resolution in comparison to metagenomics studies. Future metagenomics studies are needed to refine the herein-presented association pattern, exploring potential differences within one genus. The EpiPath cohort consists of only 115 participants. This is a considerable number for a study on ELA, in which a full psychosocial stress test was performed, together with full immune and psychological profiling. However, this sample size is considered small for a microbiome studies, where statistical screening leads to multiple testing, reducing the statistical power for detecting individual associations. Similarly, the reported mediation analyses lack statistical power, and negative results should not be interpreted as the absence of effects. Similarly, as EpiPath is an adoption cohort, metadata such as the mode of birth, if they were ever collected, were never transferred to the adoptive parents. It is also possible that our data could be interpreted as the early inoculation with different microbiomes that simply persisted until 24 years later. The invasive nature of the ELA questions meant that compromise on microbiome-specific metadata, such as dietary habits and oral health status, was unavoidable if maximum participation in the study was to be ensured. Such information would have enhanced the mechanistic potential of our dataset. Knowledge of potential oral complications such as carries or periodontitis will be necessary in future studies to ensure that mechanistic pathways can be explored [95]. As the cohort consists of observational human data, causal interpretations of the reported associations should be treated with care. However, we demonstrated that 16S sequencing, despite its known limitations, provided clear insight into the long-term effect of ELA on the microbiome. Follow-up studies using shotgun metagenomics may refine the reported associations on the species and strain level.

## 4. Materials and Methods

**Participants** For this study, we used our previously reported EpiPath cohort of 115 adults aged 20 to 25 years [19,22,51,96]. A total of 75 control participants were brought up by their biological parents and 40 participants were adopted in Luxembourg from institutions worldwide. The median age at adoption was 4.3 months (IQR 0–15 months) [51]. Basic immunoprofiling was available for all cohort members [22,51]. Furthermore, detailed NK cell profiling was available for 76 participants (19 cases and 57 controls), and immunosenescence profiles were available for 79 participants (19 ELA and 60 controls) [22,52]. Biobanked oral swabs were available for 98 participants (33 ELA and 65 controls) and buccal swabs for all 115 participants (40 ELA and 75 controls). For one participant without immunosenescence profiling, the body mass index and sex were missing. This individual was excluded from statistical analyses, where the BMI and/or sex were used as covariates.

**Oral samples** Saliva samples were collected using Salimetrics Oral Swabs (Salimetrics, Cambridge, UK). Salivary cortisol levels have previously been reported from these samples [19,96]. Buccal swabs were collected with Isoxelix Buccal Swabs (Isohelix, Harrietsham, U.K.). Microbial DNA was extracted using Qiagen DNA from a body fluids kit (Qiagen, Venlo, The Netherlands) according to the manufacturer’s protocol. Samples were quantified with Qubit 1.2 (Invitrogen, Merelbeke, Belgium) and quality was assessed with a Nanodrop (Thermofisher, Merelbeke, Belgium). The V4 region of the 16S gene was amplified from bacterial DNA using 515F [97] and 806R [98] forward and reverse primers (Eurogentec, Seraing, Belgium). The amplification reagents and library preparation were performed using a Quick-16S kit and its equivalent dual indexes (BaseClear, Leiden, The Netherlands) using the manufacturer’s low microbial DNA concentration protocol. Libraries were quantified with Qubit, 1.2, 1.4 (Thermofisher, Merelbeke, Belgium); quality and size were assessed using a BioAnalyser (Agilent, Diegem, Belgium). Sequencing was performed on an Illumina MiSeq system with v2 sequencing chemistry and 500 bp paired-end reads, as well as 10% PHIX control according to the manufacturer’s protocol.

**Bioinformatic analyses** Fastq files were processed, aligned, and classified using mothur 1.41v [99]. Alpha (inverse Simpson diversity index and Shannon evenness index) and beta diversity (Jaccard Index) were further calculated in the same pipeline. Sequences classification was aligned based on the Silva v138 database [100]. Further integration of microbiome data into the immunophenotype and metadata as well as visualisations were performed with R.

**Statistical analyses** For descriptive statistics, nominal variables are described by proportions, while metric variables are described by means and standard deviations. Evenness and Shannon entropy metrics were calculated for the OM as measures of alpha diversity and compared between ELA cases and controls with Wilcoxon rank sum tests. Additionally, diversity measures were compared across the OM using rank correlations. For investigating statistical associations between taxonomical units and immune-cell numbers, relative abundances for all genera were checked for outliers. Observations that were outliers both in immune-cell numbers and relative abundances (more than four standard deviations away the mean) were excluded from the analyses, when analysing genus–immune-cell associations. Only genera, or phyla, detected in more than 50% of all cases, were analysed. The microbial abundance data were analysed using fractional regressions [101,102]. Fractional regressions are semiparametric methods not relying on distributional assumptions, and are specifically designed for the analyses of relative abundance data, making them suitable for the analysis of microbiome data, as different species abundances may not be sampled from the same class of distributions. Fractional regressions can be parametrised by odds ratios, allowing for easy interpretation of the regression coefficients in terms of the chance that a certain sequence read is assigned to a taxonomic unit [102]. All fractional regression models, if not specified otherwise, included age, BMI, and sex as covariates, and were performed separately for the OM communities. The basic covariates were included mainly to reduce residual variance and thereby increase statistical power to detect associations with the predictor of interests. Using fractional regressions, we screened the microbiome for associations with the study group variable, basic covariates (age, sex, body mass index (BMI), and smoking), antibody titres for Epstein–Barr virus (EBV), cytomegalovirus (CMV), and the herpes simplex virus (HSV), immunosenescence markers, as well as immune cell counts. All *p*-values are reported two-tailed. Statistical analyses were performed in STATA 16/MP (College Station, TX, USA), and correction for multiple testing was performed by applying the false discovery rate (FDR) [103]. An FDR < 0.05 was considered to be significant. Summary statistics of the performed analyses are given in Supplementary Tables S1–S4.

## 5. Conclusions

Our data show a clear link between ELA and the OM that was visible 24 years later. The two oral communities investigated were clearly associated but distinct. We previously reported that ELA induced higher activation and senescence of the immune system. The taxonomic differences in the oral composition were not only associated with ELA but also with the immunosenescence of CD4 T cells, circulating numbers and activation status of NK cells, and anti-CMV titres. Although we do not yet have a detailed mechanistic explanation, our data suggest the presence of multiple links between ELA, immunosenescence, and cytotoxicity that persist through long-term changes in the microbiome.

## Figures and Tables

**Table 1:** An overview of the bacterial taxa associated with all the tested covariates in both saliva and buccal microbiomes.

	Saliva			Buccal		
	Number of Associations (FDR < 0.05)	Positively Associated Taxa	Negatively Associated Taxa	Number of Associations (FDR < 0.05)	Positively Associated Taxa	Negatively Associated Taxa
Smoking	0	-	-	5	-	*Pasteurellaceae* (unclassified), *Neisseria*, *Capnocytophaga*, *Neisseriaceae* (unc ^1^), *Planococcaceae* (unc)
ELA vs. control	4	-	*Aquabacterium*, *Comamonas*, *Comamonadaceae* (unc), *Acinetobacter*	0	-	-
Anti-CMV seropositive	2	-	*Pseudomonas*, *Oxalobaceraceae* (unc)	9	*Alysiella*, *Neisseria*	*Sphingomonas*, *Acinetobacter*, *Oxalobacteraceae* (unc), *Bradyrhizobium*, *Flavobacterium*, *Methylorubrum*, *Comamonadaceae* (unc)
Anti-EBV seropositive	0	-	-	1	*Neisseria*	-
HSV	0	-	-	0	-	-
CD4+ CD57+	2	*Selenomonas*	*Oxalobaceraceae* (unc)	4	*Selenomonas*, *Capnocytophaga*, *Campylobacter*, *Lautropia*	-
CD8+ CD57+	0	-	-	0	-	-
Total CTLs	0	-	-	0	-	-
Total T_h_ cells	0	-	-	0	-	-

^1^ unc = unclassified.

## Data Availability

All data from the EpiPath study are available upon reasonable request to J.D.T.

## Supplementary Materials

The following are available online at https://www.mdpi.com/article/10.3390/ijms222312682/s1.
