# Supplementary material for: Early-Life Adversity Leaves Its Imprint on the Oral Microbiome for More Than 20 Years and Is Associated with Long-Term Immune Changes
Source: Int J Mol Sci. 2021 Nov 24;22(23):12682. doi: 10.3390/ijms222312682 (PMC8657988; doi:10.3390/ijms222312682)
Supplement: Supplementary file 1 [file ijms-22-12682-s001.zip › Charalambous et al_Table_S1.pdf]

Table S1A: Associations of Phyla abundances with ELA-control status

Buccal Microbiome					
**Phylum**	**OR**	**95%-CI**	**p-value**	**rank**	**FDR**
Actinobacteriota	7.58E-01	(.4914952815;1.170459032)	2.12E-01	1	1.9052834
Unclassified bacteria	8.71E-01	(.4112909559;1.846240043)	7.19E-01	2	3.237039
Bacteroidota	1.10E+00	(.7738764914;1.563458515)	5.95E-01	3	1.786069
Campilobacterota	1.39E+00	(.7683524563;2.510154577)	2.77E-01	4	0.6228861
Firmicutes	1.30E+00	(.9060110626;1.877701568)	1.53E-01	5	0.2753866
Fusobacteriota	1.18E+00	(.6666524577;2.072883642)	5.76E-01	6	0.8644104
Patescibacteria	1.01E+00	(.4278179668;2.401568621)	9.75E-01	7	1.2541764
Proteobacteria	7.81E-01	(.5336716021;1.142814416)	2.03E-01	8	0.2284925
Spirochaetota	5.59E-01	(.2389226677;1.309704256)	1.81E-01	9	0.1807781

Saliva Microbiome					
**Phylum**	**OR**	**95%-CI**	**p-value**	**rank**	**FDR**
Actinobacteriota	1.43E+00	(1.028270245;1.986050248)	3.35E-02	1	0.3350537
Unclassified bacteria	7.33E-01	(.535617897;1.003453152)	5.26E-02	2	0.2628482
Bacteroidota	1.24E+00	(.6671648134;2.309373082)	4.95E-01	3	1.6501608
Campilobacterota	1.29E+00	(.7479432582;2.21607497)	3.62E-01	4	0.9046579
Firmicutes	1.25E+00	(.9441143205;1.653706757)	1.19E-01	5	0.2385642
Fusobacteriota	1.48E+00	(.954452545;2.297947367)	7.98E-02	6	0.1329604
Patescibacteria	7.16E-01	(.3969076555;1.29307585)	2.68E-01	7	0.3833293
Planctomycetota	8.22E-01	(.2995985008;2.254657337)	7.03E-01	8	0.8790293
**Proteobacteria**	**5.44E-01**	**(.3739821786;.790428986)**	**1.41E-03**	9	**0.0015708**
**Verrucomicrobiota**	**1.77E-01**	**(.0654450077;.4791024834)**	**6.52E-04**	10	**0.0006522**

OR=odds ratio; 95%-CI=95% confidence inter

p-values derived from fractional regressions adjusted for study group, age, bmi, and sex

Table S1B: Association with ELA of genera found in the saliva microbiome

Genus	OR	95%-CI	p-value	FDR
Aquabacterium	2.57E-01	(.1364154182;.483071718)	2.49E-05	1.22E-03
Comamonas	3.46E-01	(.2061736645;.5795940595)	5.61E-05	1.38E-03
Comamonadaceae_unclassified	3.83E-01	(.2395134643;.6136883574)	6.49E-05	1.06E-03
Acinetobacter	3.54E-01	(.2069970161;.605956567)	1.52E-04	1.86E-03
Weeksellaceae_unclassified	4.92E-01	(.2961217507;.8190095049)	6.35E-03	6.22E-02
Bradyrhizobium	5.42E-01	(.3468853293;.8459965948)	7.03E-03	5.74E-02
Oxalobacteraceae_unclassified	4.03E-01	(.2013851106;.8071226344)	1.03E-02	7.22E-02
Paucibacter	3.90E-01	(.1894384691;.8019501984)	1.05E-02	6.42E-02
Sphingomonas	4.15E-01	(.2084395274;.8262490815)	1.23E-02	6.70E-02
Flavobacterium	4.86E-01	(.2701063477;.8727796432)	1.57E-02	7.72E-02
NA	1.50E+00	(1.072632668;2.105646087)	1.79E-02	7.98E-02
Atopobium	2.59E+00	(1.134622937;5.932681973)	2.39E-02	9.75E-02
Alloprevotella	7.03E+00	(1.267110332;39.00038663)	2.57E-02	9.69E-02
Oribacterium	5.19E-01	(.263902236;1.019239439)	5.68E-02	1.99E-01
Fusobacterium	1.52E+00	(.9385796738;2.472651615)	8.85E-02	2.89E-01
Xanthobacteraceae_unclassified	3.90E-01	(.131489919;1.154565537)	8.90E-02	2.73E-01
Afipia	5.76E-01	(.2915407406;1.138795331)	1.13E-01	3.25E-01
Porphyromonas	1.56E+00	(.8863140516;2.737311194)	1.23E-01	3.36E-01
Streptobacillus	1.39E+00	(.8838666978;2.175504637)	1.55E-01	3.99E-01
Micrococcaceae_unclassified	1.39E+00	(.8640676605;2.22108193)	1.76E-01	4.31E-01
Caulobacter	4.67E-01	(.1517535936;1.435327085)	1.84E-01	4.29E-01
Alysiella	1.96E+00	(.6928627392;5.572535413)	2.04E-01	4.55E-01
Rothia	1.65E+00	(.7071812886;3.828159678)	2.48E-01	5.28E-01
Actinobacillus	1.40E+00	(.7314270578;2.665124775)	3.12E-01	6.36E-01
Methylobacterium-Methylorubrum	7.45E-01	(.4203345224;1.318730583)	3.12E-01	6.11E-01
Haemophilus	1.79E+00	(.5715340277;5.626585486)	3.17E-01	5.97E-01
Campylobacter	1.32E+00	(.7659698965;2.277299588)	3.17E-01	5.75E-01
Veillonella	1.32E+00	(.7522301501;2.319957063)	3.33E-01	5.82E-01
Neisseriaceae_unclassified	1.55E+00	(.6278386319;3.824849683)	3.42E-01	5.78E-01
Actinomyces	1.23E+00	(.7849806603;1.934168768)	3.64E-01	5.95E-01
Catonella	7.63E-01	(.4190428346;1.389519426)	3.77E-01	5.95E-01
Prevotella	1.31E+00	(.6714528482;2.565547534)	4.26E-01	6.53E-01
Pseudomonas	6.82E-01	(.2434170647;1.912801083)	4.67E-01	6.94E-01
Streptococcus	1.11E+00	(.8392410082;1.457779044)	4.74E-01	6.83E-01
Enterococcaceae_unclassified	1.31E+00	(.5543141736;3.082120872)	5.41E-01	7.57E-01
Cardiobacterium	1.18E+00	(.6471943295;2.157764796)	5.87E-01	7.99E-01
Lautropia	8.34E-01	(.4100107348;1.694946426)	6.15E-01	8.15E-01
Pasteurellaceae_unclassified	8.28E-01	(.3926821535;1.746303905)	6.20E-01	8.00E-01
Planococcaceae_unclassified	9.18E-01	(.6337713011;1.328928236)	6.49E-01	8.16E-01
Prevotellaceae_ge	8.02E-01	(.2846835833;2.26046182)	6.77E-01	8.29E-01
Fluviicola	8.59E-01	(.409493832;1.80218177)	6.88E-01	8.22E-01
Lachnoanaerobaculum	1.14E+00	(.4984749436;2.594551891)	7.60E-01	8.87E-01
Absconditabacteriales_(SR1)_ge	8.88E-01	(.3004725401;2.624752818)	8.30E-01	9.46E-01
Corynebacterium	1.04E+00	(.5928416891;1.807956393)	9.03E-01	1.01E+00
Capnocytophaga	9.66E-01	(.5330604877;1.750105903)	9.09E-01	9.90E-01
Neisseria	1.02E+00	(.6090655307;1.721462713)	9.29E-01	9.89E-01
Prevotellaceae_unclassified	1.03E+00	(.3662794033;2.921160924)	9.49E-01	9.89E-01

Table S1c: Associations of ELA in association with genera found in the buccal microbiome

Genus	OR	95%-CI	p-value	FDR
Actinobacillus	5.08E-01	(.3086350199;.8373503615)	7.88E-03	3.86E-01
Prevotella	1.53E+00	(.9118341947;2.570592052)	1.07E-01	2.63E+00
Micrococcaceae_unclassified	7.03E-01	(.4482886246;1.101153501)	1.24E-01	2.02E+00
Alloprevotella	5.56E-01	(.2593771756;1.190048538)	1.30E-01	1.60E+00
Parvimonas	2.19E+00	(.7492105022;6.42233874)	1.52E-01	1.49E+00
Treponema	5.76E-01	(.2435808739;1.360352511)	2.08E-01	1.70E+00
Catonella	1.60E+00	(.7534537039;3.382478786)	2.22E-01	1.55E+00
Veillonella	1.33E+00	(.8346179681;2.121042835)	2.30E-01	1.41E+00
Sphingomonas	4.95E-01	(.154215271;1.590542221)	2.38E-01	1.29E+00
Oribacterium	1.65E+00	(.6934776952;3.925220149)	2.58E-01	1.26E+00
Campylobacter	1.39E+00	(.7684413903;2.511308854)	2.77E-01	1.23E+00
Lachnoanaerobaculum	1.37E+00	(.7655392143;2.467760074)	2.87E-01	1.17E+00
Pasteurellaceae_unclassified	5.26E-01	(.1594361419;1.736155135)	2.92E-01	1.10E+00
Neisseria	1.32E+00	(.7795644327;2.24896748)	2.99E-01	1.05E+00
Neisseriaceae_unclassified	1.68E+00	(.6052043796;4.690231685)	3.18E-01	1.04E+00
Acinetobacter	1.76E+00	(.5793678164;5.327071667)	3.19E-01	9.78E-01
Paucibacter	5.60E-01	(.1666177252;1.883860192)	3.49E-01	1.01E+00
Weeksellaceae_unclassified	1.31E+00	(.7290202577;2.367987824)	3.64E-01	9.90E-01
Bradyrhizobium	6.18E-01	(.2150952346;1.77799016)	3.72E-01	9.60E-01
Terrisporobacter	1.50E+00	(.6040566535;3.717971527)	3.83E-01	9.38E-01
Comamonadaceae_unclassified	6.15E-01	(.2004370391;1.887777383)	3.96E-01	9.23E-01
Anaerovoracaceae_ge	1.53E+00	(.5744129543;4.063554053)	3.96E-01	8.82E-01
Solobacterium	1.38E+00	(.6392022272;2.980568951)	4.12E-01	8.78E-01
Fusobacterium	1.22E+00	(.7483967648;1.99798928)	4.22E-01	8.62E-01
Cardiobacterium	6.32E-01	(.2043585979;1.953467795)	4.25E-01	8.34E-01
Planococcaceae_unclassified	7.34E-01	(.3422259954;1.572688093)	4.26E-01	8.03E-01
Lautropia	7.14E-01	(.2817486546;1.808219909)	4.77E-01	8.66E-01
Streptococcus	1.16E+00	(.7500878084;1.806954195)	4.98E-01	8.71E-01
Tannerella	1.31E+00	(.5956024207;2.870501761)	5.04E-01	8.51E-01
Rothia	7.86E-01	(.384991727;1.604226753)	5.08E-01	8.30E-01
Haemophilus	1.22E+00	(.5747054638;2.605271395)	6.01E-01	9.49E-01
Oxalobacteraceae_unclassified	7.88E-01	(.30982799;2.003747121)	6.17E-01	9.44E-01
Enterococcaceae_unclassified	7.91E-01	(.2762110363;2.267024518)	6.63E-01	9.84E-01
NA	9.47E-01	(.7278918193;1.233164029)	6.88E-01	9.92E-01
Atopobium	8.56E-01	(.3948313465;1.857492956)	6.95E-01	9.73E-01
Streptobacillus	1.13E+00	(.5368584069;2.387016415)	7.45E-01	1.01E+00
Capnocytophaga	8.65E-01	(.3561172145;2.101378153)	7.49E-01	9.92E-01
Porphyromonas	1.08E+00	(.6706403576;1.736332059)	7.54E-01	9.72E-01
Selenomonas	1.11E+00	(.575777507;2.136733357)	7.57E-01	9.51E-01
Actinomyces	9.06E-01	(.4221076858;1.943527047)	7.99E-01	9.79E-01
Lachnospiraceae_unclassified	9.24E-01	(.4628872577;1.845945626)	8.24E-01	9.84E-01
Veillonellaceae_unclassified	1.06E+00	(.5672208221;1.963839752)	8.65E-01	1.01E+00
Alysiella	1.07E+00	(.3856954288;2.942103626)	9.03E-01	1.03E+00
Prevotellaceae_unclassified	9.75E-01	(.5594377958;1.698960359)	9.29E-01	1.03E+00
Methylobacterium-Methylorubrum	9.50E-01	(.3101126275;2.911983292)	9.29E-01	1.01E+00
Flavobacterium	1.05E+00	(.2951584787;3.730920206)	9.41E-01	1.00E+00
Afipia	9.60E-01	(.2275626716;4.046963619)	9.55E-01	9.96E-01
Prevotellaceae_ge	1.01E+00	(.5012802881;2.016770805)	9.88E-01	1.01E+00
Corynebacterium	9.96E-01	(.42535939;2.332319976)	9.93E-01	9.93E-01

OR=Odds ratio; 95%-CI= 95% confidence interval
p-values derived from fractional regressions adjusted for study group, age, bmi, and sex
