# Supplementary material for: Early-Life Adversity Leaves Its Imprint on the Oral Microbiome for More Than 20 Years and Is Associated with Long-Term Immune Changes
Source: Int J Mol Sci. 2021 Nov 24;22(23):12682. doi: 10.3390/ijms222312682 (PMC8657988; doi:10.3390/ijms222312682)
Supplement: Supplementary file 1 [file ijms-22-12682-s001.zip › Charalambous et al_Table_S2.pdf]

Table S2A: Associations of smoking status with genera found in the buccal microbiome

Genus	OR	95%-CI	p-value	FDR
**Pasteurellaceae_unclassified**	**0.18416**	**(.0882504192;.3842884818)**	**6.5408E-06**	**3.07E-04**
**Neisseria**	**0.33383**	**(.2039517418;.5464300441)**	**1.2784E-05**	**3.00E-04**
**Capnocytophaga**	**0.27789**	**(.1534441642;.5032734525)**	**2.3796E-05**	**3.73E-04**
**Neisseriaceae_unclassified**	**0.28664**	**(.1411815038;.5819628069)**	**0.00054379**	**6.39E-03**
**Planococcaceae_unclassified**	**0.34890**	**(.179864814;.6767849401)**	**0.00184061**	**1.73E-02**
Corynebacterium	0.42418	(.2193716352;.8202173354)	0.01080199	8.46E-02
Weeksellaceae_unclassified	0.53038	(.3242522222;.8675378043)	0.01153863	7.75E-02
Cardiobacterium	0.32054	(.1161503068;.8846009319)	0.02804108	1.65E-01
Haemophilus	0.54382	(.3148748185;.9392304492)	0.028901	1.51E-01
Veillonellaceae_unclassified	2.11181	(1.059560491;4.2090631)	0.03363892	1.58E-01
Oxalobacteraceae_unclassified	0.39179	(.1613492956;.951361892)	0.0384412	1.64E-01
Fusobacterium	0.59367	(.358775015;.9823572047)	0.04243416	1.66E-01
Oribacterium	0.52066	(.2560685275;1.058643641)	0.07145597	2.58E-01
Porphyromonas	0.61857	(.3549683334;1.077932699)	0.09005094	3.02E-01
Catonella	0.55791	(.2804210358;1.109976033)	0.09637583	3.02E-01
Prevotellaceae_ge	0.60216	(.3240466825;1.118977351)	0.10862859	3.19E-01
Streptobacillus	0.61679	(.3206511607;1.186413327)	0.14766641	4.08E-01
Atopobium	1.52644	(.8254818663;2.822631925)	0.17750441	4.63E-01
Lachnospiraceae_unclassified	0.67242	(.3740666452;1.208734816)	0.18471088	4.57E-01
Micrococcaceae_unclassified	1.44444	(.8340184609;2.501644397)	0.18943143	4.45E-01
Terrisporobacter	0.50871	(.1722908945;1.502004118)	0.2211252	4.95E-01
Anaerovoracaceae_ge	0.50915	(.1715331514;1.511254372)	0.22396566	4.78E-01
Comamonadaceae_unclassified	0.54970	(.1962189486;1.539987533)	0.25492457	5.21E-01
Paucibacter	0.60971	(.2495634568;1.489591279)	0.27765813	5.44E-01
Streptococcus	1.21863	(.8247183574;1.800691873)	0.32091931	6.03E-01
Actinomyces	0.73037	(.3864796891;1.380241884)	0.33325067	6.02E-01
Tannerella	0.67336	(.2902902462;1.561930046)	0.35693207	6.21E-01
Lachnoanaerobaculum	0.79063	(.4705503426;1.328429408)	0.37490973	6.29E-01
Sphingomonas	0.69353	(.2973974625;1.617326302)	0.3969427	6.43E-01
Alysiella	0.59286	(.1705252558;2.061161882)	0.4108938	6.44E-01
Parvimonas	0.50698	(.0909333241;2.826543475)	0.4384554	6.65E-01
Acinetobacter	0.56266	(.1295988542;2.442855988)	0.44268471	6.50E-01
Actinobacillus	1.25329	(.6964998062;2.255176673)	0.45130089	6.43E-01
Solobacterium	1.36372	(.5896676559;3.153855002)	0.46833372	6.47E-01
Veillonella	1.16998	(.7267474407;1.883519804)	0.51816761	6.96E-01
Campylobacter	0.77455	(.3278297307;1.830008466)	0.56032062	7.32E-01
Prevotellaceae_unclassified	0.76938	(.3066283531;1.930479527)	0.57645446	7.32E-01
Rothia	0.78659	(.3384295361;1.828202482)	0.57693684	7.14E-01
Bradyrhizobium	0.81445	(.351836189;1.885353872)	0.63176292	7.61E-01
Treponema	1.30889	(.3475984942;4.928657739)	0.69069713	8.12E-01
Methylobacterium-Methylorubrum	0.81054	(.271579459;2.419102882)	0.70653796	8.10E-01
Lautropia	0.82530	(.2772654051;2.456561317)	0.7300846	8.17E-01
Afipia	1.17706	(.3532532331;3.922003595)	0.79065382	8.64E-01
Enterococcaceae_unclassified	1.07698	(.409054992;2.8355092)	0.88065273	9.41E-01
Unclassified	0.97541	(.6665871114;1.427295538)	0.89798546	9.38E-01
Alloprevotella	1.07841	(.2998557306;3.878443781)	0.90797186	9.28E-01
Selenomonas	1.05892	(.3676922102;3.049603199)	0.91551656	9.16E-01
Prevotella	0.98800	(.5997700558;1.627539347)	0.96219885	9.42E-01
Flavobacterium	0.97845	(.2407624143;3.976355485)	0.97570193	9.36E-01

OR=odds ratio; 95%-CI=95% confidence interval
p-values from fractional regressions adjusting for group variable, age, bmi, and sex

Table S2B: Associations of smoking status with the genera found in the saliva

Genus	OR	95%-CI	p-value	FDR
Catonella	4.49E-01	(.2178701123;.9239007714)	2.97E-02	1.39E+00
Sphingomonas	2.09E+00	(1.054048893;4.158110399)	3.48E-02	8.19E-01
Campylobacter	1.78E+00	(1.029160714;3.091013508)	3.92E-02	6.14E-01
Lachnospiraceae_unclassified	3.40E+00	(1.011134838;11.41645349)	4.79E-02	5.63E-01
Pasteurellaceae_unclassified	4.86E-01	(.2243812132;1.053247321)	6.75E-02	6.34E-01
Haemophilus	3.88E-01	(.1332542203;1.132300821)	8.32E-02	6.52E-01
Lachnoanaerobaculum	5.77E-01	(.3062045523;1.088777565)	8.97E-02	6.02E-01
Lautropia	4.87E-01	(.2054590259;1.155204734)	1.03E-01	6.03E-01
Porphyromonas	1.63E+00	(.8222330698;3.23320732)	1.62E-01	8.44E-01
Selenomonas	1.68E+00	(.7778708236;3.635443888)	1.86E-01	8.76E-01
Prevotella	1.49E+00	(.8154635591;2.722295092)	1.95E-01	8.32E-01
Streptobacillus	1.41E+00	(.8297158372;2.395909687)	2.04E-01	7.99E-01
Enterococcaceae_unclassified	1.67E+00	(.7433440156;3.770470627)	2.14E-01	7.72E-01
Planococcaceae_unclassified	7.67E-01	(.495558488;1.188571072)	2.36E-01	7.91E-01
Fluviicola	4.39E-01	(.1048779745;1.836144267)	2.59E-01	8.13E-01
Caulobacter	1.78E+00	(.6104679477;5.180458497)	2.91E-01	8.56E-01
Cardiobacterium	6.10E-01	(.2266756276;1.64193808)	3.28E-01	9.07E-01
Capnocytophaga	1.32E+00	(.6969022919;2.500641907)	3.94E-01	1.03E+00
Unclassified	8.46E-01	(.5744322516;1.245226795)	3.96E-01	9.80E-01
Streptococcus	8.61E-01	(.6050799814;1.226485866)	4.08E-01	9.59E-01
Pseudomonas	1.52E+00	(.5440082029;4.250562061)	4.24E-01	9.49E-01
Prevotellaceae_unclassified	7.09E-01	(.29691096;1.694905257)	4.40E-01	9.39E-01
Oribacterium	7.40E-01	(.3262751314;1.679870196)	4.72E-01	9.65E-01
Afipia	7.62E-01	(.3594085408;1.615692782)	4.78E-01	9.37E-01
Atopobium	1.33E+00	(.590336806;2.977766283)	4.94E-01	9.30E-01
Oxalobacteraceae_unclassified	7.70E-01	(.361068115;1.642306744)	4.99E-01	9.02E-01
Bradyrhizobium	1.19E+00	(.7215804692;1.949226296)	5.01E-01	8.72E-01
Rothia	1.32E+00	(.5826126071;3.003762325)	5.04E-01	8.45E-01
Absconditabacteriales_(SR1)_ge	6.89E-01	(.2006580313;2.362483728)	5.53E-01	8.96E-01
Corynebacterium	8.44E-01	(.4737418894;1.504653319)	5.66E-01	8.87E-01
Fusobacterium	1.17E+00	(.6504030087;2.096779569)	6.03E-01	9.15E-01
Veillonella	1.14E+00	(.6637544329;1.942251723)	6.43E-01	9.44E-01
Actinomyces	8.93E-01	(.5530969504;1.442330678)	6.44E-01	9.17E-01
Flavobacterium	8.71E-01	(.4596317047;1.64955152)	6.71E-01	9.28E-01
Comamonadaceae_unclassified	1.09E+00	(.6501999703;1.84088433)	7.35E-01	9.87E-01
Alloprevotella	8.27E-01	(.2170728994;3.149249643)	7.80E-01	1.02E+00
Paucibacter	1.13E+00	(.4782237914;2.652560535)	7.86E-01	9.98E-01
Alysiella	9.11E-01	(.3973690661;2.08743373)	8.25E-01	1.02E+00
Micrococcaceae_unclassified	9.27E-01	(.4720416202;1.82009375)	8.26E-01	9.95E-01
Methylobacterium-	1.07E+00	(.5605169305;2.036298688)	8.41E-01	9.88E-01
Aquabacterium	9.33E-01	(.4546546443;1.914635951)	8.50E-01	9.74E-01
Actinobacillus	1.06E+00	(.5738762913;1.957941877)	8.52E-01	9.54E-01
Neisseria	1.07E+00	(.5071073349;2.250675006)	8.62E-01	9.42E-01
Neisseriaceae_unclassified	1.06E+00	(.4257837993;2.622608895)	9.05E-01	9.67E-01
Prevotellaceae_ge	1.05E+00	(.4371056404;2.504544405)	9.19E-01	9.60E-01
Xanthobacteraceae_unclassified	1.03E+00	(.3602690552;2.966221156)	9.51E-01	9.71E-01
Weeksellaceae_unclassified	9.81E-01	(.5334960996;1.804577306)	9.51E-01	9.51E-01
Acinetobacter	9.82E-01	(.5458440949;1.768217137)	9.53E-01	9.33E-01
Comamonas	9.93E-01	(.5539973779;1.778921291)	9.80E-01	9.40E-01

OR=Odds ratio; 95%-CI= 95% confidence interval
p-values derived from fractional regressions adjusted for study group, age, bmi, and sex
