# Supplementary material for: Early-Life Adversity Leaves Its Imprint on the Oral Microbiome for More Than 20 Years and Is Associated with Long-Term Immune Changes
Source: Int J Mol Sci. 2021 Nov 24;22(23):12682. doi: 10.3390/ijms222312682 (PMC8657988; doi:10.3390/ijms222312682)
Supplement: Supplementary file 1 [file ijms-22-12682-s001.zip › Charalambous et al_Table_S3.pdf]

Table S3A: Associations of positive HSV antibody titres with genera found in the buccal microbiome

Genus	OR	95-CI	p-value	FDR
Enterococcaceae_unclassified	3,35E+00	(.9628683457;11.64643429)	5,74E-02	2,70E+00
Selenomonas	5,40E-01	(.2788621867;1.04632112)	6,79E-02	1,60E+00
Parvimonas	3,95E-01	(.1324966716;1.178222939)	9,58E-02	1,50E+00
Weeksellaceae_unclassified	1,49E+00	(.9211803043;2.42121669)	1,04E-01	1,22E+00
Prevotellaceae_ge	6,15E-01	(.3408508629;1.110118895)	1,07E-01	1,00E+00
Neisseria	1,65E+00	(.8946744056;3.039679424)	1,09E-01	8,53E-01
Planococcaceae_unclassified	2,06E+00	(.7741410336;5.488473493)	1,48E-01	9,92E-01
Solobacterium	6,12E-01	(.3013980616;1.243285737)	1,75E-01	1,03E+00
Oribacterium	5,66E-01	(.2407441551;1.332309532)	1,93E-01	1,01E+00
Micrococcaceae_unclassified	6,59E-01	(.3343847474;1.29753849)	2,27E-01	1,07E+00
Neisseriaceae_unclassified	1,64E+00	(.7208346166;3.7391844)	2,38E-01	1,02E+00
Porphyromonas	1,46E+00	(.776753025;2.739489394)	2,40E-01	9,41E-01
Rothia	1,54E+00	(.6567505769;3.606997212)	3,21E-01	1,16E+00
Actinomyces	1,63E+00	(.6131240319;4.316730054)	3,28E-01	1,10E+00
Actinobacillus	1,37E+00	(.6965900768;2.697983449)	3,61E-01	1,13E+00
Streptococcus	8,28E-01	(.5424127238;1.264526593)	3,83E-01	1,12E+00
Bradyrhizobium	6,36E-01	(.22866965;1.769707755)	3,86E-01	1,07E+00
Prevotella	7,98E-01	(.4644193011;1.372140506)	4,15E-01	1,08E+00
Acinetobacter	6,38E-01	(.2009560188;2.022454693)	4,45E-01	1,10E+00
Unclassified	8,54E-01	(.5669064655;1.286924482)	4,51E-01	1,06E+00
Lachnospiraceae_unclassified	7,95E-01	(.422354663;1.495937984)	4,77E-01	1,07E+00
Lachnoanaerobaculum	8,21E-01	(.4767435882;1.414223604)	4,77E-01	1,02E+00
Sphingomonas	7,05E-01	(.2494143104;1.990722399)	5,09E-01	1,04E+00
Flavobacterium	1,39E+00	(.4539338822;4.281798437)	5,62E-01	1,10E+00
Alysiella	7,82E-01	(.3299219752;1.855133145)	5,77E-01	1,09E+00
Treponema	7,73E-01	(.272051533;2.19467501)	6,28E-01	1,14E+00
Lautropia	1,24E+00	(.5126911378;2.976217418)	6,38E-01	1,11E+00
Tannerella	8,17E-01	(.3513477912;1.900274637)	6,39E-01	1,07E+00
Cardiobacterium	1,25E+00	(.476727934;3.286611084)	6,48E-01	1,05E+00
Veillonellaceae_unclassified	8,18E-01	(.3409510907;1.961091714)	6,52E-01	1,02E+00
Campylobacter	8,76E-01	(.4771318838;1.607075393)	6,68E-01	1,01E+00
Paucibacter	7,95E-01	(.265524989;2.378979952)	6,81E-01	1,00E+00
Veillonella	9,00E-01	(.5415619513;1.494045411)	6,82E-01	9,72E-01
Corynebacterium	8,38E-01	(.3527420845;1.988766814)	6,88E-01	9,51E-01
Prevotellaceae_unclassified	8,66E-01	(.4228538674;1.77538639)	6,95E-01	9,34E-01
Atopobium	8,67E-01	(.4150086562;1.812605724)	7,05E-01	9,21E-01
Comamonadaceae_unclassified	8,10E-01	(.2714806476;2.418714988)	7,06E-01	8,97E-01
Alloprevotella	1,18E+00	(.4798436169;2.915479215)	7,15E-01	8,85E-01
Catonella	1,13E+00	(.5444304631;2.360883081)	7,37E-01	8,89E-01
Haemophilus	1,13E+00	(.5456298494;2.330217852)	7,46E-01	8,76E-01
Oxalobacteraceae_unclassified	1,17E+00	(.3736236956;3.641728979)	7,91E-01	9,07E-01
Terrisporobacter	1,11E+00	(.4641920298;2.664110004)	8,12E-01	9,08E-01
Pasteurellaceae_unclassified	8,75E-01	(.2903405405;2.639091347)	8,13E-01	8,89E-01
Fusobacterium	9,47E-01	(.585056988;1.533973466)	8,26E-01	8,82E-01
Streptobacillus	1,07E+00	(.5743139048;1.999981326)	8,28E-01	8,64E-01
Methylobacterium-Methylorubrum	1,07E+00	(.3750089347;3.055195953)	8,99E-01	9,18E-01

Capnocytophaga	9,82E-01	(.4808890728;2.007138201)	9,61E-01	9,61E-01

OR=Odds ratio; 95%-CI=95% confidence interval

p-values derived from fractional regressions

HSV = Herpes Simplex Virus

Table S3B: Associations of positive HSV antibody titres with genera found in the saliva microbiome

Genus	OR	95-CI	p-value	FDR
Streptococcus	6,73E-01	(.4762567362;.950904716)	2,47E-02	1,21E+00
Paucibacter	2,08E+00	(.9526141238;4.523622353)	6,61E-02	1,62E+00
Aquabacterium	1,90E+00	(.9530647114;3.80504014)	6,82E-02	1,11E+00
Comamonas	1,75E+00	(.9402841895;3.245352111)	7,75E-02	9,50E-01
Xanthobacteraceae_unclassified	2,68E+00	(.8705195495;8.273284229)	8,57E-02	8,40E-01
Corynebacterium	6,23E-01	(.361809606;1.073580765)	8,84E-02	7,22E-01
Comamonadaceae_unclassified	1,64E+00	(.8919296244;3.022671354)	1,11E-01	7,79E-01
Acinetobacter	1,66E+00	(.8462000406;3.271440334)	1,40E-01	8,57E-01
Atopobium	4,60E-01	(.1619051592;1.307172099)	1,45E-01	7,90E-01
Prevotellaceae_ge	2,25E+00	(.7356453322;6.889760033)	1,55E-01	7,60E-01
Enterococcaceae_unclassified	4,89E-01	(.1821647606;1.314144259)	1,56E-01	6,96E-01
Alloprevotella	3,06E-01	(.0585105554;1.596071447)	1,60E-01	6,53E-01
Neisseriaceae_unclassified	2,06E+00	(.743502462;5.690955817)	1,65E-01	6,21E-01
Bradyrhizobium	1,55E+00	(.7921959967;3.046126322)	2,00E-01	6,99E-01
Weeksellaceae_unclassified	1,54E+00	(.7729001407;3.058772093)	2,20E-01	7,19E-01
Actinomyces	7,22E-01	(.4278388766;1.219815884)	2,24E-01	6,85E-01
Planococcaceae_unclassified	7,57E-01	(.4498268339;1.273917444)	2,94E-01	8,49E-01
Capnocytophaga	1,50E+00	(.6640412523;3.376440991)	3,31E-01	9,00E-01
Selenomonas	6,56E-01	(.2522803842;1.7037345)	3,86E-01	9,96E-01
Caulobacter	1,68E+00	(.5053222113;5.556920776)	3,99E-01	9,77E-01
Fluviicola	1,66E+00	(.5105455722;5.386289944)	4,00E-01	9,34E-01
Catonella	1,39E+00	(.6365005886;3.018604796)	4,11E-01	9,15E-01
Prevotellaceae_unclassified	6,98E-01	(.2720166959;1.790916662)	4,55E-01	9,68E-01
Absconditabacteriales_(SR1)_ge	5,83E-01	(.1124948971;3.024015053)	5,21E-01	1,06E+00
Porphyromonas	1,22E+00	(.6393518525;2.330602678)	5,46E-01	1,07E+00
Sphingomonas	1,21E+00	(.6283460638;2.327446854)	5,69E-01	1,07E+00
Lachnoanaerobaculum	1,40E+00	(.4351532221;4.479590387)	5,75E-01	1,04E+00
Methylobacterium-Methylorubrum	1,18E+00	(.6439313098;2.150443118)	5,97E-01	1,04E+00
Flavobacterium	1,19E+00	(.6206626074;2.288069966)	5,98E-01	1,01E+00
Rothia	1,24E+00	(.5553310255;2.746971763)	6,05E-01	9,88E-01
Cardiobacterium	8,00E-01	(.3376093846;1.897447529)	6,13E-01	9,69E-01
Unclassified	8,91E-01	(.5502304482;1.441945069)	6,38E-01	9,77E-01
Lachnospiraceae_unclassified	7,81E-01	(.2749092583;2.219238527)	6,43E-01	9,55E-01
Campylobacter	8,66E-01	(.4465938715;1.67862164)	6,70E-01	9,65E-01
Pasteurellaceae_unclassified	1,25E+00	(.416324462;3.754098625)	6,91E-01	9,67E-01
Fusobacterium	8,83E-01	(.4771163371;1.635253816)	6,93E-01	9,43E-01
Prevotella	8,74E-01	(.4417628565;1.730356352)	7,00E-01	9,27E-01
Pseudomonas	1,23E+00	(.3732801316;4.075033407)	7,31E-01	9,42E-01
Actinobacillus	1,14E+00	(.521964377;2.496094909)	7,40E-01	9,30E-01
Oribacterium	8,45E-01	(.2820837624;2.531281443)	7,64E-01	9,35E-01
Afipia	1,11E+00	(.5595891236;2.200596108)	7,66E-01	9,15E-01
Streptobacillus	9,11E-01	(.4910492634;1.68846246)	7,66E-01	8,94E-01
Parvimonas	8,78E-01	(.3055037671;2.52354993)	8,09E-01	9,22E-01
Micrococcaceae_unclassified	9,31E-01	(.4763113827;1.821524122)	8,36E-01	9,31E-01
Veillonella	1,07E+00	(.5459367568;2.083750636)	8,50E-01	9,26E-01
Oxalobacteraceae_unclassified	1,07E+00	(.5102061446;2.245528462)	8,57E-01	9,13E-01

Lautropia	9,36E-01	(.4390055011;1.995707582)	8,64E-01	9,01E-01
Neisseria	9,67E-01	(.4789839646;1.953593574)	9,26E-01	9,46E-01
Terrisporobacter	9,74E-01	(.3381818226;2.802361985)	9,60E-01	9,60E-01

OR=Odds ratio; 95%-CI=95% confidence interval

p-values derived from fractional regressions

HSV = Herpes Simplex Virus

Table S3C: Associations of positive EBV antibody titres with genera found in the buccal microbiome

Genus	OR	95-CI	p-value	FDR
**Neisseria**	**2,55E+00**	**(1.519815814;4.276392071)**	**3,91E-04**	**1,84E-02**
Prevotella	1,75E+00	(1.019355173;3.017072194)	4,24E-02	9,97E-01
Oribacterium	2,45E+00	(1.016869998;5.926943565)	4,58E-02	7,18E-01
Neisseriaceae_unclassified	2,08E+00	(1.010529863;4.265418499)	4,68E-02	5,49E-01
Terrisporobacter	2,45E+00	(.9984653;6.019178626)	5,04E-02	4,74E-01
Tannerella	2,20E+00	(.9537875398;5.074257758)	6,45E-02	5,05E-01
Fusobacterium	1,54E+00	(.9429643789;2.51549918)	8,45E-02	5,67E-01
Streptobacillus	1,65E+00	(.9054165242;2.992975121)	1,02E-01	6,01E-01
Weeksellaceae_unclassified	1,47E+00	(.9123244172;2.370758338)	1,13E-01	5,92E-01
Porphyromonas	1,52E+00	(.8654030694;2.655219735)	1,46E-01	6,85E-01
Lachnospiraceae_unclassified	1,59E+00	(.8504892982;2.98419736)	1,46E-01	6,23E-01
Parvimonas	2,08E+00	(.6923908118;6.267411668)	1,92E-01	7,50E-01
Catonella	1,62E+00	(.7819942955;3.335721742)	1,95E-01	7,06E-01
Campylobacter	1,53E+00	(.7954178067;2.955996962)	2,02E-01	6,77E-01
Solobacterium	1,75E+00	(.7290744553;4.220516903)	2,10E-01	6,57E-01
Prevotellaceae_ge	1,46E+00	(.7906246781;2.699158525)	2,26E-01	6,65E-01
Lachnoanaerobaculum	1,39E+00	(.7990242627;2.407730548)	2,45E-01	6,77E-01
Acinetobacter	1,82E+00	(.6306751539;5.272519381)	2,67E-01	6,98E-01
Enterococcaceae_unclassified	1,96E+00	(.5559871875;6.905981028)	2,95E-01	7,30E-01
Capnocytophaga	1,41E+00	(.7350987327;2.697996604)	3,02E-01	7,10E-01
Streptococcus	8,15E-01	(.5469974931;1.214058852)	3,14E-01	7,03E-01
Actinobacillus	7,34E-01	(.3963013321;1.35984501)	3,26E-01	6,96E-01
Pasteurellaceae_unclassified	6,11E-01	(.2190499908;1.701821887)	3,46E-01	7,06E-01
Alysiella	1,50E+00	(.6435465626;3.476883436)	3,49E-01	6,84E-01
Rothia	1,46E+00	(.6561701749;3.228225389)	3,56E-01	6,69E-01
Micrococcaceae_unclassified	7,45E-01	(.3955422543;1.404951962)	3,64E-01	6,57E-01
Haemophilus	1,40E+00	(.6698416861;2.922936413)	3,71E-01	6,47E-01
Unclassified	8,40E-01	(.5690919993;1.240013905)	3,80E-01	6,38E-01
Oxalobacteraceae_unclassified	1,43E+00	(.4644796717;4.373085379)	5,36E-01	8,68E-01
Cardiobacterium	1,35E+00	(.4906724754;3.707911381)	5,62E-01	8,80E-01
Prevotellaceae_unclassified	1,23E+00	(.6089440126;2.489685643)	5,62E-01	8,53E-01
Flavobacterium	7,56E-01	(.2670576234;2.139859707)	5,98E-01	8,79E-01
Lautropia	8,08E-01	(.3352457181;1.949272613)	6,36E-01	9,05E-01
Corynebacterium	1,24E+00	(.4927786948;3.137673436)	6,45E-01	8,91E-01
Paucibacter	1,33E+00	(.3821393951;4.655133431)	6,52E-01	8,75E-01
Selenomonas	1,19E+00	(.5173963598;2.730495427)	6,84E-01	8,93E-01
Comamonadaceae_unclassified	8,27E-01	(.2622629139;2.60831964)	7,46E-01	9,48E-01
Alloprevotella	8,87E-01	(.3553550826;2.215186819)	7,98E-01	9,87E-01
Sphingomonas	1,16E+00	(.3346169116;4.010647079)	8,16E-01	9,84E-01
Veillonellaceae_unclassified	1,08E+00	(.4645604175;2.508659429)	8,59E-01	1,01E+00
Methylobacterium-Methylorubrum	1,09E+00	(.3998762032;2.978219345)	8,65E-01	9,91E-01
Veillonella	1,04E+00	(.6331578603;1.71628183)	8,70E-01	9,74E-01
Treponema	9,15E-01	(.3161084922;2.650583236)	8,70E-01	9,51E-01
Bradyrhizobium	1,07E+00	(.3524177507;3.273529065)	9,00E-01	9,61E-01
Atopobium	9,77E-01	(.5057621153;1.886680538)	9,44E-01	9,86E-01
Actinomyces	1,02E+00	(.3922071432;2.655739239)	9,67E-01	9,88E-01
Planococcaceae_unclassified	9,82E-01	(.3498383532;2.757418781)	9,73E-01	9,73E-01

OR=Odds ratio; 95%-CI=95% confidence interval
p-values derived from fractional regressions
EBV = Ebstein-Barr virus

Table S3D: Associations of positive EBV antibody titres with genera found in the saliva microbiome

Genus	OR	95-CI	p-value	FDR
Absconditabacteriales_(SR1)_ge	1,96E-01	(.0674918827;.5716781914)	2,83E-03	1,39E-01
Sphingomonas	5,05E-01	(.2800379012;.9121646701)	2,35E-02	5,76E-01
Bradyrhizobium	5,35E-01	(.3059798667;.9348298007)	2,81E-02	4,58E-01
Afipia	5,74E-01	(.3050025099;1.078908846)	8,47E-02	1,04E+00
Alloprevotella	4,13E+00	(.7018452647;24.31870307)	1,17E-01	1,14E+00
Pseudomonas	2,09E+00	(.7420355939;5.893271527)	1,63E-01	1,33E+00
Xanthobacteraceae_unclassified	5,06E-01	(.1822368373;1.402388253)	1,90E-01	1,33E+00
Veillonella	1,47E+00	(.7987436624;2.690942594)	2,17E-01	1,33E+00
Methylobacterium-Methylorubrum	6,90E-01	(.3781642102;1.257873701)	2,26E-01	1,23E+00
Weeksellaceae_unclassified	6,96E-01	(.3694203355;1.31283686)	2,63E-01	1,29E+00
Lautropia	6,94E-01	(.3450827502;1.396426421)	3,06E-01	1,36E+00
Pasteurellaceae_unclassified	1,59E+00	(.6286834141;4.028053147)	3,27E-01	1,33E+00
Paucibacter	6,51E-01	(.2682473669;1.580298066)	3,43E-01	1,29E+00
Caulobacter	5,44E-01	(.1509340703;1.962499407)	3,53E-01	1,23E+00
Parvimonas	6,69E-01	(.2862487065;1.562217248)	3,53E-01	1,15E+00
Lachnospiraceae_unclassified	6,53E-01	(.2383256413;1.790979395)	4,08E-01	1,25E+00
Actinobacillus	1,36E+00	(.6462695164;2.848853971)	4,20E-01	1,21E+00
Corynebacterium	8,03E-01	(.4681248676;1.378312178)	4,26E-01	1,16E+00
Streptobacillus	1,27E+00	(.7068053212;2.270367061)	4,27E-01	1,10E+00
Flavobacterium	1,29E+00	(.6843949188;2.446155756)	4,28E-01	1,05E+00
Streptococcus	8,86E-01	(.6227781951;1.259464724)	4,99E-01	1,16E+00
Selenomonas	1,28E+00	(.5913886801;2.764571987)	5,32E-01	1,19E+00
Micrococcaceae_unclassified	1,21E+00	(.6642021223;2.205541093)	5,33E-01	1,14E+00
Planococcaceae_unclassified	8,34E-01	(.4706627104;1.476926083)	5,33E-01	1,09E+00
Rothia	1,30E+00	(.5464250639;3.075294906)	5,56E-01	1,09E+00
Catonella	1,24E+00	(.6002377731;2.574155491)	5,58E-01	1,05E+00
Comamonas	8,28E-01	(.4352022231;1.577208423)	5,67E-01	1,03E+00
Prevotella	8,23E-01	(.4188208289;1.616587552)	5,71E-01	1,00E+00
Terrisporobacter	1,29E+00	(.5164830423;3.245560204)	5,82E-01	9,83E-01
Actinomyces	1,14E+00	(.708362172;1.8397106)	5,87E-01	9,58E-01
Enterococcaceae_unclassified	7,74E-01	(.29697448;2.016050205)	6,00E-01	9,48E-01
Atopobium	7,62E-01	(.2733313142;2.123821853)	6,03E-01	9,24E-01
Porphyromonas	8,43E-01	(.4419423588;1.608422766)	6,05E-01	8,98E-01
Prevotellaceae_unclassified	1,30E+00	(.4684171789;3.61091097)	6,14E-01	8,85E-01
Cardiobacterium	8,34E-01	(.3877008673;1.793228622)	6,42E-01	8,99E-01
Fusobacterium	1,13E+00	(.6415672081;2.006847512)	6,64E-01	9,04E-01
Lachnoanaerobaculum	1,25E+00	(.4353701168;3.601679381)	6,76E-01	8,96E-01
Neisseriaceae_unclassified	1,22E+00	(.4318214998;3.448550802)	7,07E-01	9,12E-01
Oribacterium	1,19E+00	(.410514237;3.460494457)	7,47E-01	9,38E-01
Oxalobacteraceae_unclassified	8,86E-01	(.4209140542;1.866608404)	7,51E-01	9,20E-01
Acinetobacter	9,01E-01	(.4537345826;1.787737501)	7,65E-01	9,14E-01
Aquabacterium	8,96E-01	(.4288631199;1.869986928)	7,69E-01	8,97E-01
Comamonadaceae_unclassified	9,23E-01	(.4929546805;1.727832164)	8,02E-01	9,14E-01
Capnocytophaga	9,06E-01	(.4057324481;2.02155892)	8,09E-01	9,01E-01
Campylobacter	1,07E+00	(.575073453;2.001303897)	8,25E-01	8,99E-01
Fluviicola	1,13E+00	(.3604394943;3.511361993)	8,39E-01	8,94E-01
Unclassified	9,61E-01	(.6303399941;1.46607459)	8,55E-01	8,91E-01

Prevotellaceae_ge	1,07E+00	(.3133629531;3.658495777)	9,13E-01	9,32E-01
Neisseria	1,01E+00	(.5203947428;1.96417889)	9,74E-01	9,74E-01

OR=Odds ratio; 95%-CI=95% confidence interval

p-values derived from fractional regressions

EBV = Ebstein-Barr virus

Table S3E: Associations of positive CMV antibody titres with genera found in the buccal microbiome

Genus	OR	95-CI	p-value	FDR
**Sphingomonas**	**9,25E-02**	**(.0357713752;.2394287092)**	**9,23E-07**	**4,34E-05**
**Acinetobacter**	**1,15E-01**	**(.0487637334;.2735630572)**	**9,28E-07**	**2,18E-05**
**Oxalobacteraceae_unclassified**	**1,83E-01**	**(.086996909;.3863687417)**	**8,19E-06**	**1,28E-04**
**Bradyrhizobium**	**2,14E-01**	**(.0937337942;.4864120848)**	**2,37E-04**	**2,79E-03**
**Flavobacterium**	**2,40E-01**	**(.1047194475;.5478757044)**	**7,11E-04**	**6,68E-03**
**Methylobacterium-Methylorubrum**	**2,12E-01**	**(.0812142408;.5508005392)**	**1,47E-03**	**1,15E-02**
**Comamonadaceae_unclassified**	**2,66E-01**	**(.1025200124;.6912306567)**	**6,56E-03**	**4,40E-02**
**Alysiella**	**3,53E+00**	**(1.421307232;8.752763873)**	**6,57E-03**	**3,86E-02**
**Neisseria**	**2,90E+00**	**(1.320194017;6.355304747)**	**7,98E-03**	**4,17E-02**
Paucibacter	2,46E-01	(.0792483749;.7645702479)	1,53E-02	7,21E-02
Actinobacillus	4,86E-01	(.2613144501;.9055257247)	2,30E-02	9,84E-02
Solobacterium	2,71E+00	(1.131176943;6.49773642)	2,53E-02	9,92E-02
Catonella	1,89E+00	(1.006822283;3.550213487)	4,76E-02	1,72E-01
Lachnospiraceae_unclassified	1,89E+00	(.9916072066;3.584464747)	5,31E-02	1,78E-01
Enterococcaceae_unclassified	3,89E-01	(.145983963;1.036667469)	5,90E-02	1,85E-01
Campylobacter	1,94E+00	(.8928194749;4.223966787)	9,41E-02	2,76E-01
Terrisporobacter	1,97E+00	(.8057305674;4.829442597)	1,37E-01	3,79E-01
Cardiobacterium	2,24E+00	(.7694458503;6.541771219)	1,39E-01	3,63E-01
Pasteurellaceae_unclassified	4,96E-01	(.1948316375;1.264438316)	1,42E-01	3,51E-01
Capnocytophaga	1,84E+00	(.7969297621;4.245402848)	1,53E-01	3,60E-01
Prevotella	1,44E+00	(.82887464;2.503937922)	1,95E-01	4,38E-01
Selenomonas	2,05E+00	(.6883654875;6.109957351)	1,97E-01	4,21E-01
Veillonellaceae_unclassified	2,08E+00	(.6714530871;6.470525406)	2,04E-01	4,16E-01
Haemophilus	7,12E-01	(.4176297771;1.213611138)	2,12E-01	4,15E-01
Planococcaceae_unclassified	2,49E+00	(.5917394194;10.46453917)	2,14E-01	4,01E-01
Corynebacterium	1,70E+00	(.7149410647;4.023799396)	2,31E-01	4,17E-01
Veillonella	1,44E+00	(.7566445018;2.747288697)	2,66E-01	4,63E-01
Streptococcus	7,07E-01	(.3770055132;1.324990634)	2,79E-01	4,68E-01
Prevotellaceae_unclassified	1,61E+00	(.6503251915;3.965128421)	3,04E-01	4,93E-01
Oribacterium	1,98E+00	(.5092684962;7.680896486)	3,25E-01	5,08E-01
Treponema	1,77E+00	(.3974612799;7.887390791)	4,54E-01	6,88E-01
Neisseriaceae_unclassified	1,47E+00	(.5017389536;4.320031923)	4,81E-01	7,07E-01
Fusobacterium	1,18E+00	(.7179832733;1.946489259)	5,11E-01	7,27E-01
Streptobacillus	1,17E+00	(.6980882396;1.961887876)	5,51E-01	7,61E-01
Parvimonas	1,53E+00	(.3665898423;6.409261982)	5,58E-01	7,50E-01
Rothia	1,51E+00	(.3509114773;6.492731852)	5,80E-01	7,57E-01
Lachnoanaerobaculum	1,17E+00	(.6676387996;2.052488652)	5,82E-01	7,40E-01
Unclassified	1,16E+00	(.6709853799;1.998766477)	5,98E-01	7,40E-01
Atopobium	1,17E+00	(.6423764227;2.116623596)	6,14E-01	7,39E-01
Lautropia	1,24E+00	(.4235017045;3.630642815)	6,95E-01	8,16E-01
Prevotellaceae_ge	1,09E+00	(.6198600273;1.906104512)	7,71E-01	8,84E-01
Porphyromonas	9,17E-01	(.5077249673;1.656670483)	7,74E-01	8,67E-01
Alloprevotella	9,25E-01	(.4638447576;1.845049926)	8,25E-01	9,02E-01
Tannerella	1,09E+00	(.4586829325;2.579319605)	8,49E-01	9,07E-01
Weeksellaceae_unclassified	1,05E+00	(.5301220754;2.091904194)	8,83E-01	9,22E-01
Micrococcaceae_unclassified	1,02E+00	(.3862107682;2.697546233)	9,67E-01	9,88E-01

Actinomyces	1,00E+00	(.3915465875;2.572170637)	9,94E-01	9,94E-01

OR=Odds ratio; 95%-CI=95% confidence interval

p-values derived from fractional regressions

CMV = Cytomegalovirus

Table S3F: Associations of positive CMV antibody titres with genera found in the saliva microbiome

Genus	OR	95-CI	p-value	FDR
**Pseudomonas**	**5,95E-02**	**(.0211642304;.1670737399)**	**8,57E-08**	**4,20E-06**
**Oxalobacteraceae_unclas**	**2,93E-01**	**(.1758440061;.489146861)**	**2,60E-06**	**6,38E-05**
Flavobacterium	4,00E-01	(.1981722504;.8089339426)	1,07E-02	1,76E-01
Parvimonas	2,67E+00	(1.077628966;6.638138841)	3,39E-02	4,15E-01
Bradyrhizobium	5,00E-01	(.2565350729;.9744169952)	4,17E-02	4,09E-01
Selenomonas	2,54E+00	(1.022141438;6.308954359)	4,47E-02	3,65E-01
Actinobacillus	4,34E-01	(.1908425595;.9861104736)	4,62E-02	3,24E-01
Fluviicola	2,96E-01	(.0881195409;.9968409695)	4,94E-02	3,03E-01
Lachnospiraceae_unclassi	4,20E+00	(.9798272016;17.9879751)	5,33E-02	2,90E-01
Afipia	3,97E-01	(.1491868771;1.058190158)	6,48E-02	3,17E-01
Weeksellaceae_unclassifie	4,55E-01	(.184492126;1.12072314)	8,68E-02	3,87E-01
Lautropia	5,65E-01	(.275653974;1.159854948)	1,20E-01	4,89E-01
Comamonadaceae_unclas	5,25E-01	(.2185908962;1.2632997)	1,51E-01	5,67E-01
Micrococcaceae_unclassif	1,79E+00	(.7751095209;4.115697346)	1,73E-01	6,06E-01
Cardiobacterium	4,42E-01	(.1346214362;1.453830234)	1,79E-01	5,85E-01
Actinomyces	1,74E+00	(.7534154074;4.014640808)	1,95E-01	5,96E-01
Corynebacterium	1,54E+00	(.7866242519;2.998513877)	2,09E-01	6,02E-01
Alloprevotella	3,42E-01	(.0618024342;1.894096593)	2,19E-01	5,97E-01
Atopobium	2,26E+00	(.608401361;8.370440095)	2,24E-01	5,77E-01
Prevotellaceae_ge	4,78E-01	(.1420881008;1.605268874)	2,32E-01	5,69E-01
Pasteurellaceae_unclassifi	2,41E+00	(.5373418047;10.76838073)	2,51E-01	5,86E-01
Paucibacter	6,24E-01	(.2753803735;1.412433576)	2,58E-01	5,74E-01
Methylobacterium-Methy	6,70E-01	(.3226053031;1.39200817)	2,83E-01	6,03E-01
Comamonas	5,66E-01	(.1962630619;1.630329589)	2,91E-01	5,95E-01
Veillonella	6,07E-01	(.2360554468;1.560916232)	3,00E-01	5,88E-01
Prevotella	5,75E-01	(.1867103922;1.770501416)	3,35E-01	6,31E-01
Absconditabacteriales_(SR	5,97E-01	(.1992876232;1.789527178)	3,57E-01	6,48E-01
Acinetobacter	5,84E-01	(.1848795313;1.844687947)	3,59E-01	6,29E-01
Aquabacterium	6,38E-01	(.2440708084;1.669085681)	3,60E-01	6,08E-01
Xanthobacteraceae_uncla	5,06E-01	(.1172265501;2.182188311)	3,61E-01	5,89E-01
Catonella	6,85E-01	(.286976681;1.633143895)	3,93E-01	6,21E-01
Sphingomonas	7,05E-01	(.2994656778;1.657443691)	4,22E-01	6,47E-01
Oribacterium	6,03E-01	(.1709073775;2.124681805)	4,31E-01	6,40E-01
Prevotellaceae_unclassifie	1,53E+00	(.5251598984;4.475179632)	4,34E-01	6,26E-01
Capnocytophaga	1,72E+00	(.4046269539;7.275411511)	4,64E-01	6,49E-01
Unclassified	1,16E+00	(.7477427724;1.812365245)	5,01E-01	6,82E-01
Neisseriaceae_unclassified	1,76E+00	(.3320056382;9.310044899)	5,07E-01	6,71E-01
Terrisporobacter	7,24E-01	(.237954618;2.199828271)	5,68E-01	7,33E-01
Caulobacter	7,56E-01	(.2360829082;2.419129001)	6,37E-01	8,00E-01
Streptococcus	1,08E+00	(.6591399529;1.775981768)	7,55E-01	9,25E-01
Neisseria	8,60E-01	(.2692237387;2.74910613)	8,00E-01	9,56E-01
Porphyromonas	1,08E+00	(.4360705941;2.698120533)	8,61E-01	1,00E+00
Campylobacter	9,06E-01	(.2882590717;2.845635621)	8,65E-01	9,86E-01
Fusobacterium	1,05E+00	(.5146679608;2.133045436)	8,98E-01	1,00E+00
Enterococcaceae_unclassi	1,07E+00	(.348173641;3.267229585)	9,10E-01	9,91E-01
Rothia	1,04E+00	(.3756501792;2.866691486)	9,43E-01	1,00E+00

Lachnoanaerobaculum	9,69E-01	(.3485101071;2.696846441)	9,53E-01	9,93E-01
Streptobacillus	1,01E+00	(.4717928378;2.170012064)	9,76E-01	9,96E-01
Planococcaceae_unclassif	1,01E+00	(.6110941744;1.658662739)	9,79E-01	9,79E-01

OR=Odds ratio; 95%-CI=95% confidence interval

p-values derived from fractional regressions

CMV = Cytomegalovirus
