# Supplementary material for: Early-Life Adversity Leaves Its Imprint on the Oral Microbiome for More Than 20 Years and Is Associated with Long-Term Immune Changes
Source: Int J Mol Sci. 2021 Nov 24;22(23):12682. doi: 10.3390/ijms222312682 (PMC8657988; doi:10.3390/ijms222312682)
Supplement: Supplementary file 1 [file ijms-22-12682-s001.zip › Charalambous et al_Table_S4.pdf]

Table S4A: Associations of CD57 positive CD8 cell counts with genera found in the buccal microbiome

Genus	OR	95%-CI	p-value	FDR
Selenomonas	1,06E+00	(1.019047306;1.105776267)	4,16E-03	1,96E-01
Pasteurellaceae_unclassified	1,05E+00	(1.014175348;1.07806829)	4,20E-03	9,86E-02
Campylobacter	1,05E+00	(1.015501471;1.088003461)	4,59E-03	7,19E-02
Streptococcus	9,73E-01	(.9545970331;.9916711372)	4,80E-03	5,64E-02
Lachnoanaerobaculum	1,04E+00	(1.01087326;1.064701517)	5,48E-03	5,16E-02
Lautropia	1,06E+00	(1.016710044;1.114811689)	7,69E-03	6,03E-02
Capnocytophaga	1,05E+00	(1.009383493;1.085781094)	1,38E-02	9,28E-02
Fusobacterium	1,02E+00	(1.000953915;1.048653758)	4,13E-02	2,43E-01
Neisseriaceae_unclassified	1,03E+00	(.9983529783;1.070198994)	6,19E-02	3,23E-01
Flavobacterium	1,03E+00	(.9960616421;1.072950235)	7,98E-02	3,75E-01
Lachnospiraceae_unclassified	1,03E+00	(.9957935117;1.06250037)	8,82E-02	3,77E-01
Parvimonas	1,05E+00	(.9931812929;1.10204458)	8,87E-02	3,48E-01
Comamonadaceae_unclassified	1,03E+00	(.9935216073;1.066245746)	1,10E-01	3,97E-01
Tannerella	1,04E+00	(.9908586845;1.094108495)	1,10E-01	3,70E-01
Cardiobacterium	1,03E+00	(.9913667356;1.074084418)	1,25E-01	3,90E-01
Prevotella	1,02E+00	(.9925562751;1.054911216)	1,39E-01	4,09E-01
Streptobacillus	1,03E+00	(.9913970299;1.061580761)	1,43E-01	3,95E-01
Prevotellaceae_unclassified	1,03E+00	(.9860034954;1.076320522)	1,84E-01	4,80E-01
Bradyrhizobium	1,03E+00	(.9879011373;1.063970983)	1,88E-01	4,65E-01
Rothia	1,02E+00	(.9914316638;1.041818567)	2,01E-01	4,72E-01
Solobacterium	1,04E+00	(.9807062837;1.092931286)	2,09E-01	4,69E-01
Actinomyces	1,03E+00	(.9817233365;1.086941314)	2,11E-01	4,52E-01
Treponema	1,04E+00	(.9744569692;1.114258574)	2,29E-01	4,68E-01
Unclassified	1,01E+00	(.9909613353;1.037358048)	2,37E-01	4,64E-01
Haemophilus	1,02E+00	(.9847784185;1.062071643)	2,44E-01	4,59E-01
Terrisporobacter	1,03E+00	(.9804805524;1.079443658)	2,48E-01	4,47E-01
Corynebacterium	1,02E+00	(.9840409753;1.060535339)	2,64E-01	4,59E-01
Actinobacillus	9,85E-01	(.9525978545;1.018481283)	3,75E-01	6,30E-01
Sphingomonas	1,01E+00	(.9727615077;1.058059621)	5,02E-01	8,13E-01
Planococcaceae_unclassified	9,85E-01	(.9418891723;1.02975254)	5,02E-01	7,86E-01
Methylobacterium-Methylorubru	1,01E+00	(.9720015295;1.059196211)	5,07E-01	7,68E-01
Porphyromonas	1,01E+00	(.9804928536;1.040545873)	5,09E-01	7,47E-01
Veillonellaceae_unclassified	1,02E+00	(.958326418;1.088505412)	5,16E-01	7,35E-01
Enterococcaceae_unclassified	9,78E-01	(.913630674;1.046934422)	5,22E-01	7,22E-01
Prevotellaceae_ge	1,01E+00	(.9831681154;1.032974039)	5,40E-01	7,25E-01
Veillonella	1,01E+00	(.9838403182;1.030758467)	5,56E-01	7,26E-01
Alloprevotella	9,87E-01	(.9414214294;1.03527617)	5,96E-01	7,57E-01
Catonella	1,01E+00	(.9744802875;1.045429362)	6,04E-01	7,48E-01
Oxalobacteraceae_unclassified	1,01E+00	(.9727007222;1.047643502)	6,18E-01	7,45E-01
Weeksellaceae_unclassified	9,94E-01	(.9693857715;1.018755148)	6,22E-01	7,30E-01
Oribacterium	9,94E-01	(.9597643455;1.029161018)	7,29E-01	8,36E-01
Neisseria	1,00E+00	(.9761130927;1.031902412)	7,99E-01	8,94E-01
Paucibacter	1,01E+00	(.9593440451;1.054043504)	8,17E-01	8,93E-01
Alysiella	1,00E+00	(.9626760823;1.047531872)	8,46E-01	9,03E-01
Acinetobacter	1,00E+00	(.9662916018;1.037541426)	9,44E-01	9,86E-01

Micrococcaceae_unclassified	9,99E-01	(.9536615638;1.046475038)	9,66E-01	9,87E-01
Atopobium	1,00E+00	(.9680550367;1.033455878)	9,89E-01	9,89E-01

OR=odds ratio; 95%-CI=95% confidence interval

p-values from fractional regressions adjusting for group variable, age, bmi, and sex

Table S4B: Associations of CD57 positive CD8 cell counts with genera found in the saliva microbiome

Genus	OR	95%-CI	p-value	FDR
Actinomyces	1,03E+00	(1.005497914;1.045091151)	1,19E-02	5,81E-01
Selenomonas	1,04E+00	(1.009266303;1.079550071)	1,25E-02	3,07E-01
Enterococcaceae_unclassified	9,46E-01	(.901462782;.9936663461)	2,67E-02	4,36E-01
Prevotellaceae_ge	9,08E-01	(.8318479498;.9921185036)	3,27E-02	4,00E-01
Atopobium	1,03E+00	(1.002453137;1.066836389)	3,45E-02	3,38E-01
Unclassified	1,02E+00	(1.001031144;1.038644977)	3,85E-02	3,14E-01
Lachnospiraceae_unclassified	1,06E+00	(.9961896611;1.130732339)	6,55E-02	4,58E-01
Parvimonas	1,04E+00	(.9974842242;1.080877303)	6,62E-02	4,06E-01
Xanthobacteraceae_unclassified	1,03E+00	(.9963341151;1.060658457)	8,37E-02	4,55E-01
Comamonadaceae_unclassified	9,75E-01	(.9470632044;1.004589174)	9,78E-02	4,79E-01
Neisseriaceae_unclassified	9,72E-01	(.9391661582;1.006295926)	1,09E-01	4,85E-01
Absconditabacteriales_(SR1)_ge	1,08E+00	(.9814828161;1.198914434)	1,11E-01	4,53E-01
Oxalobacteraceae_unclassified	9,75E-01	(.9431248868;1.008959446)	1,49E-01	5,63E-01
Capnocytophaga	9,80E-01	(.9511511725;1.009001021)	1,72E-01	6,03E-01
Porphyromonas	9,80E-01	(.9516514996;1.009010096)	1,74E-01	5,69E-01
Oribacterium	9,65E-01	(.9142906792;1.018482301)	1,95E-01	5,98E-01
Corynebacterium	1,02E+00	(.9910577811;1.040050761)	2,19E-01	6,30E-01
Flavobacterium	9,82E-01	(.9540348603;1.010864121)	2,19E-01	5,97E-01
Neisseria	9,76E-01	(.9387634908;1.014742842)	2,21E-01	5,71E-01
Veillonella	9,76E-01	(.9357380701;1.017723181)	2,54E-01	6,23E-01
Aquabacterium	9,82E-01	(.9489336121;1.015398443)	2,82E-01	6,59E-01
Comamonas	9,80E-01	(.9435732779;1.017096288)	2,83E-01	6,30E-01
Fusobacterium	9,86E-01	(.9620553622;1.011389834)	2,84E-01	6,04E-01
Weeksellaceae_unclassified	9,74E-01	(.9269173028;1.024013449)	3,05E-01	6,22E-01
Fluviicola	9,81E-01	(.9465024616;1.017576773)	3,09E-01	6,06E-01
Actinobacillus	9,75E-01	(.9278769413;1.025172057)	3,26E-01	6,14E-01
Micrococcaceae_unclassified	1,02E+00	(.9798089546;1.062761193)	3,29E-01	5,97E-01
Streptobacillus	9,89E-01	(.9664579052;1.011874642)	3,41E-01	5,97E-01
Acinetobacter	9,85E-01	(.9515834844;1.019014058)	3,78E-01	6,39E-01
Pseudomonas	9,76E-01	(.924911746;1.03086017)	3,89E-01	6,35E-01
Lachnoanaerobaculum	1,02E+00	(.9786002307;1.054988612)	4,06E-01	6,41E-01
Rothia	1,01E+00	(.983121972;1.038984985)	4,52E-01	6,92E-01
Caulobacter	9,78E-01	(.9218767787;1.038529069)	4,74E-01	7,04E-01
Streptococcus	9,94E-01	(.9787162623;1.010364908)	4,90E-01	7,07E-01
Planococcaceae_unclassified	1,01E+00	(.9796229393;1.043790627)	4,91E-01	6,88E-01
Catonella	9,88E-01	(.9540620972;1.023010809)	4,95E-01	6,74E-01
Cardiobacterium	1,01E+00	(.9801524443;1.039667958)	5,31E-01	7,03E-01
Afipia	9,92E-01	(.9627857812;1.021403622)	5,79E-01	7,46E-01
Lautropia	9,91E-01	(.9613698019;1.022271674)	5,79E-01	7,28E-01
Paucibacter	9,91E-01	(.9558816622;1.026862119)	6,11E-01	7,48E-01
Prevotella	9,92E-01	(.953801864;1.030984344)	6,72E-01	8,04E-01
Campylobacter	1,01E+00	(.9721624546;1.039352469)	7,61E-01	8,88E-01
Alloprevotella	1,01E+00	(.9503825426;1.071296224)	7,69E-01	8,76E-01

Pasteurellaceae_unclassified	1,01E+00	(.965168039;1.047023888)	8,00E-01	8,91E-01
Prevotellaceae_unclassified	9,96E-01	(.9634643065;1.029982011)	8,22E-01	8,95E-01
Methylobacterium-Methylorubrum	9,97E-01	(.969616086;1.025966861)	8,56E-01	9,12E-01
Bradyrhizobium	9,99E-01	(.9759828181;1.022039042)	9,15E-01	9,54E-01
Sphingomonas	1,00E+00	(.9746438546;1.027715979)	9,51E-01	9,71E-01
Terrisporobacter	1,00E+00	(.9520322338;1.050833752)	9,93E-01	9,93E-01

OR=odds ratio; 95%-CI=95% confidence interval

p-values from fractional regressions adjusting for group variable, age, bmi, and sex

Table S4C: Associations of CD57 positive CD4 cell counts with genera found in the buccal microbiome

Genus	OR	95%-CI	p-value	FDR
**Selenomonas**	**1,49E+00**	**(1.236485177;1.800796148)**	**3,00E-05**	**1,41E-03**
**Capnocytophaga**	**1,29E+00**	**(1.11729367;1.487781755)**	**5,05E-04**	**1,19E-02**
**Campylobacter**	**1,33E+00**	**(1.11904027;1.586964657)**	**1,27E-03**	**1,99E-02**
**Unclassified**	**1,19E+00**	**(1.069179519;1.331674525)**	**1,61E-03**	**1,89E-02**
**Lautropia**	**1,45E+00**	**(1.132156916;1.867550353)**	**3,37E-03**	**3,16E-02**
**Streptococcus**	**8,22E-01**	**(.7140288254;.9460282362)**	**6,28E-03**	**4,92E-02**
Streptobacillus	1,18E+00	(1.03430999;1.337848617)	1,34E-02	8,97E-02
Paucibacter	7,11E-01	(.5349113139;.9439431986)	1,84E-02	1,08E-01
Veillonella	1,15E+00	(1.021582871;1.297250887)	2,09E-02	1,09E-01
Alysiella	1,23E+00	(1.031614151;1.47164796)	2,13E-02	9,99E-02
Oxalobacteraceae_unclassified	7,96E-01	(.6424767456;.9865974736)	3,72E-02	1,59E-01
Terrisporobacter	1,23E+00	(.9949330188;1.523053523)	5,57E-02	2,18E-01
Tannerella	1,18E+00	(.9875392457;1.414863293)	6,83E-02	2,47E-01
Lachnoanaerobaculum	1,14E+00	(.9902061871;1.305474017)	6,87E-02	2,31E-01
Actinobacillus	8,61E-01	(.7321501107;1.011787576)	6,91E-02	2,16E-01
Haemophilus	1,15E+00	(.9855646132;1.340617805)	7,59E-02	2,23E-01
Sphingomonas	7,75E-01	(.5821988524;1.031758691)	8,08E-02	2,24E-01
Comamonadaceae_unclassified	8,25E-01	(.6594102679;1.033156215)	9,39E-02	2,45E-01
Corynebacterium	1,21E+00	(.9635885962;1.526269402)	1,00E-01	2,48E-01
Prevotellaceae_ge	1,11E+00	(.976279781;1.257219459)	1,12E-01	2,64E-01
Fusobacterium	1,12E+00	(.9735972129;1.289547919)	1,13E-01	2,52E-01
Neisseria	1,13E+00	(.970547132;1.316184962)	1,15E-01	2,46E-01
Prevotella	1,12E+00	(.9709079635;1.297716031)	1,19E-01	2,42E-01
Treponema	1,33E+00	(.9222307907;1.918346905)	1,27E-01	2,48E-01
Lachnospiraceae_unclassified	1,15E+00	(.9580465002;1.380311467)	1,34E-01	2,51E-01
Bradyrhizobium	8,46E-01	(.6688687297;1.068807683)	1,60E-01	2,90E-01
Methylobacterium-Methylorubrum	8,56E-01	(.6850031323;1.06887017)	1,70E-01	2,95E-01
Porphyromonas	1,12E+00	(.9533266752;1.305693797)	1,72E-01	2,90E-01
Prevotellaceae_unclassified	1,18E+00	(.9204757323;1.522657674)	1,89E-01	3,06E-01
Actinomyces	1,18E+00	(.9079131016;1.541125812)	2,13E-01	3,34E-01
Solobacterium	1,20E+00	(.8806870371;1.636930855)	2,47E-01	3,75E-01
Acinetobacter	8,61E-01	(.6579332037;1.125644826)	2,73E-01	4,01E-01
Pasteurellaceae_unclassified	1,14E+00	(.897662423;1.456700021)	2,78E-01	3,95E-01
Micrococcaceae_unclassified	9,02E-01	(.7368122056;1.103435988)	3,15E-01	4,36E-01
Parvimonas	1,19E+00	(.82338972;1.708903461)	3,59E-01	4,82E-01
Rothia	1,10E+00	(.8685248438;1.390720739)	4,32E-01	5,64E-01
Veillonellaceae_unclassified	1,13E+00	(.7920243854;1.613825433)	4,99E-01	6,34E-01
Neisseriaceae_unclassified	1,08E+00	(.845438696;1.385900382)	5,30E-01	6,55E-01
Flavobacterium	9,48E-01	(.7873137031;1.142361823)	5,77E-01	6,95E-01
Atopobium	9,49E-01	(.7828080691;1.149404624)	5,90E-01	6,93E-01
Weeksellaceae_unclassified	1,04E+00	(.8988409698;1.20317926)	5,99E-01	6,86E-01
Planococcaceae_unclassified	9,53E-01	(.7620410724;1.190760409)	6,70E-01	7,49E-01
Alloprevotella	1,04E+00	(.8375407329;1.29152695)	7,22E-01	7,89E-01
Oribacterium	9,65E-01	(.7687986255;1.210396573)	7,56E-01	8,07E-01

Catonella	9,92E-01	(.7748146391;1.270050499)	9,49E-01	9,91E-01
Cardiobacterium	1,01E+00	(.7955170157;1.276261243)	9,50E-01	9,70E-01
Enterococcaceae_unclassified	1,00E+00	(.6531098843;1.534089818)	9,96E-01	9,96E-01

OR=odds ratio; 95%-CI=95% confidence interval

p-values from fractional regressions adjusting for group variable, age, bmi, and sex

Table S4D: Associations of CD57 positive CD4 cell counts with genera found in the saliva microbiome

Genus	OR	95%-CI	p-value	FDR
**Alloprevotella**	**1,61E+00**	**(1.226318001;2.114341673)**	**6,08E-04**	**2,98E-02**
**Oxalobacteraceae_unclassified**	**6,72E-01**	**(.5338620197;.8457295436)**	**7,05E-04**	**1,73E-02**
**Selenomonas**	**1,41E+00**	**(1.131063433;1.764371127)**	**2,32E-03**	**3,79E-02**
Absconditabacteriales_(SR1)_ge	1,85E+00	(1.182302253;2.897330645)	7,10E-03	8,69E-02
Parvimonas	1,29E+00	(1.071079739;1.565044512)	7,59E-03	7,44E-02
Actinomyces	1,16E+00	(1.026260139;1.301078338)	1,69E-02	1,38E-01
Veillonella	8,13E-01	(.6675518589;.9912206757)	4,06E-02	2,84E-01
Prevotellaceae_ge	6,33E-01	(.4065905941;.9860060131)	4,31E-02	2,64E-01
Unclassified	1,14E+00	(1.002863026;1.293902408)	4,51E-02	2,45E-01
Pseudomonas	7,18E-01	(.5103174878;1.01113344)	5,79E-02	2,84E-01
Lachnospiraceae_unclassified	1,39E+00	(.9521720616;2.038755723)	8,77E-02	3,91E-01
Corynebacterium	1,15E+00	(.9785083291;1.351696237)	8,98E-02	3,67E-01
Porphyromonas	8,61E-01	(.7158556303;1.036352849)	1,14E-01	4,29E-01
Actinobacillus	8,39E-01	(.6744229017;1.044853611)	1,17E-01	4,10E-01
Neisseria	8,47E-01	(.684908697;1.046645054)	1,24E-01	4,05E-01
Comamonadaceae_unclassified	8,66E-01	(.7182459699;1.043358424)	1,30E-01	3,98E-01
Oribacterium	7,37E-01	(.4883638805;1.11329111)	1,47E-01	4,24E-01
Bradyrhizobium	8,98E-01	(.7566639821;1.065370046)	2,17E-01	5,91E-01
Lautropia	8,67E-01	(.6880544457;1.091377313)	2,24E-01	5,77E-01
Caulobacter	7,10E-01	(.407065819;1.2382177)	2,27E-01	5,57E-01
Prevotellaceae_unclassified	1,19E+00	(.8688956734;1.636023082)	2,76E-01	6,44E-01
Aquabacterium	8,86E-01	(.7078925466;1.110043375)	2,94E-01	6,54E-01
Paucibacter	8,93E-01	(.7165829679;1.111839265)	3,11E-01	6,62E-01
Prevotella	8,91E-01	(.7065368437;1.123724466)	3,30E-01	6,73E-01
Planococcaceae_unclassified	1,05E+00	(.9333829289;1.189097296)	3,99E-01	7,82E-01
Acinetobacter	9,11E-01	(.7237963182;1.145462958)	4,24E-01	7,98E-01
Comamonas	9,23E-01	(.7564281261;1.126775404)	4,32E-01	7,84E-01
Sphingomonas	9,18E-01	(.7393517308;1.139084867)	4,36E-01	7,63E-01
Neisseriaceae_unclassified	8,58E-01	(.578030838;1.272513901)	4,46E-01	7,53E-01
Flavobacterium	9,29E-01	(.7622442094;1.132892796)	4,68E-01	7,64E-01
Methylobacterium-Methylorubrum	9,42E-01	(.7952255298;1.115754746)	4,89E-01	7,73E-01
Fusobacterium	9,51E-01	(.8245719645;1.097262661)	4,92E-01	7,54E-01
Cardiobacterium	1,08E+00	(.8514914695;1.368356165)	5,28E-01	7,84E-01
Micrococcaceae_unclassified	9,46E-01	(.7931689373;1.128406583)	5,37E-01	7,75E-01
Capnocytophaga	9,38E-01	(.7547775366;1.165386644)	5,63E-01	7,88E-01
Xanthobacteraceae_unclassified	9,17E-01	(.67404709;1.246221129)	5,78E-01	7,87E-01
Pasteurellaceae_unclassified	9,47E-01	(.762305841;1.176482302)	6,23E-01	8,25E-01
Afipia	9,46E-01	(.7595180846;1.179460182)	6,24E-01	8,05E-01
Campylobacter	1,04E+00	(.868763084;1.253891323)	6,48E-01	8,14E-01
Enterococcaceae_unclassified	9,42E-01	(.7178179033;1.236182176)	6,67E-01	8,16E-01
Atopobium	1,05E+00	(.8406844844;1.304024786)	6,82E-01	8,15E-01
Fluviicola	9,55E-01	(.7164354845;1.27355214)	7,55E-01	8,81E-01
Weeksellaceae_unclassified	9,58E-01	(.7119196314;1.289376288)	7,78E-01	8,86E-01
Streptobacillus	9,74E-01	(.7922665625;1.198526764)	8,06E-01	8,98E-01
Streptococcus	1,01E+00	(.9041987705;1.134033908)	8,28E-01	9,02E-01
Rothia	9,85E-01	(.8385974671;1.156403954)	8,51E-01	9,07E-01
Catonella	9,85E-01	(.8354361331;1.160366769)	8,53E-01	8,89E-01

Terrisporobacter	1,03E+00	(.766375289;1.378754392)	8,54E-01	8,72E-01
Lachnoanaerobaculum	1,01E+00	(.8341849177;1.223051167)	9,18E-01	9,18E-01

OR=odds ratio; 95%-CI=95% confidence interval

p-values from fractional regressions adjusting for group variable, age, bmi, and sex

Table S4E: Associations of NK cell counts with genera found in the buccal microbiome

	Genus	OR	95%-CI	p-value	NK cell markers	FDR
1	**Oribacterium**	**1.27E-01**	**(.052270834;.3061747502)**	**4.55E-06**	**NK total (log)**	**2.78E-03**
2	**Oribacterium**	**1.52E-01**	**(.0600637668;.3841906236)**	**6.87E-05**	**CD56lo (log)**	**2.10E-02**
3	Oribacterium	1.91E-01	(.0759461138;.4806797019)	4.38E-04	CD69+ CD56hi (log)	8.92E-02
4	Methylobacterium-Methylorubrum	1.34E+01	(2.994917932;60.1556996)	6.91E-04	CD56hi (sqrt)	1.06E-01
5	Solobacterium	1.41E-01	(.0449124852;.4431366546)	7.98E-04	CD56lo (log)	1.06E-01
6	Oribacterium	5.92E-01	(.4331484241;.8078948649)	9.62E-04	NK total (sqrt)	1.06E-01
7	Terrisporobacter	1.30E-01	(.0388863886;.4377563057)	9.75E-04	CD56lo (log)	1.06E-01
8	Solobacterium	1.46E-01	(.044604344;.4767953415)	1.45E-03	NK total (log)	1.10E-01
9	Terrisporobacter	1.15E-01	(.0294543847;.4473585998)	1.81E-03	NK total (log)	1.23E-01
10	Streptobacillus	3.06E+00	(1.488974358;6.290862849)	2.34E-03	CD25+ CD56lo (log)	1.43E-01
11	Terrisporobacter	5.69E-01	(.3948589775;.8197665549)	2.47E-03	CD56lo (sqrt)	1.43E-01
12	Comamonadaceae_unclassified	1.30E+01	(2.460858147;68.67354142)	2.52E-03	CD56hi (sqrt)	1.43E-01
13	Prevotellaceae_unclassified	2.46E-01	(.0954502179;.6336519016)	3.67E-03	CD69+ CD56hi (log)	1.73E-01
14	Acinetobacter	6.52E-02	(.0100871194;.4216564424)	4.15E-03	CD69+ CD56hi (log)	1.81E-01
15	Streptobacillus	2.80E+00	(1.374913776;5.710592869)	4.56E-03	CD25+ (log)	1.86E-01
16	Solobacterium	5.80E-01	(.3974536409;.8460042564)	4.69E-03	CD56lo (sqrt)	1.86E-01
17	Terrisporobacter	5.68E-01	(.383706558;.8419761602)	4.83E-03	NK total (sqrt)	1.86E-01
18	Solobacterium	5.97E-01	(.4157394151;.8571604583)	5.19E-03	NK total (sqrt)	1.86E-01
19	Actinomyces	3.17E-01	(.1410446683;.7126515045)	5.44E-03	CD25+ (log)	1.86E-01
20	Oribacterium	6.30E-01	(.4519749203;.8772948659)	6.26E-03	CD56lo (sqrt)	1.91E-01
21	Methylobacterium-Methylorubrum	1.01E+01	(1.909056061;53.48034054)	6.52E-03	CD56hi (sqrt)	1.91E-01
22	Streptobacillus	3.72E+00	(1.422786296;9.710178649)	7.37E-03	CD56hi (sqrt)	2.05E-01
23	Unclassified	2.97E-01	(.1210601215;.7293092408)	8.07E-03	CD25+ CD56hi (log)	2.14E-01
24	Alysiella	1.41E-01	(.0322429703;.6172165589)	9.30E-03	CD25+ CD56hi (log)	2.37E-01
25	Lachnoanaerobaculum	3.38E+00	(1.338748852;8.541938944)	9.97E-03	CD25+ CD56hi (log)	2.44E-01
26	Campylobacter	2.61E-01	(.0938967931;.7253746838)	1.00E-02	CD69+ CD56hi (log)	2.44E-01
27	Cardiobacterium	1.84E-01	(.0502499565;.672768094)	1.05E-02	CD69+ (log)	2.44E-01
28	Prevotellaceae_ge	3.07E+00	(1.286282426;7.322336527)	1.15E-02	CD56hi (sqrt)	2.51E-01
29	Veillonellaceae_unclassified	5.96E-01	(.3984943821;.8918699375)	1.18E-02	CD56lo (sqrt)	2.51E-01
30	Actinomyces	2.13E-01	(.0640030927;.7116891602)	1.20E-02	CD69+ CD56hi (log)	2.51E-01
31	Fusobacterium	4.01E-01	(.1963575884;.817664104)	1.20E-02	CD69+ CD56hi (log)	2.51E-01
32	Veillonellaceae_unclassified	6.01E-01	(.403718578;.8957131672)	1.24E-02	NK total (sqrt)	2.51E-01
33	Lachnoanaerobaculum	4.74E-01	(.2639741148;.8509958723)	1.24E-02	CD25+ CD56lo (log)	2.51E-01
34	Veillonella	3.38E-01	(.142256401;.801080358)	1.38E-02	CD69+ CD56hi (log)	2.51E-01
35	Streptobacillus	3.05E+00	(1.238945158;7.527901203)	1.53E-02	CD56hi (sqrt)	2.67E-01
36	Veillonellaceae_unclassified	1.66E-01	(.0386987591;.711363109)	1.56E-02	CD56lo (log)	2.67E-01
37	Veillonellaceae_unclassified	1.69E-01	(.0394770681;.72738452)	1.69E-02	NK total (log)	2.80E-01
38	Cardiobacterium	2.10E-01	(.0574183001;.768114751)	1.83E-02	CD69+ CD56lo (log)	2.95E-01
39	Parvimonas	3.68E-01	(.1570617685;.8618714675)	2.13E-02	NKT CD8+ total (log)	3.34E-01
40	Prevotella	2.54E+00	(1.148548548;5.623712881)	2.13E-02	CD25+ CD56lo (log)	3.34E-01
41	Lautropia	2.80E+00	(1.159238257;6.744038382)	2.21E-02	CD25+ (log)	3.34E-01
42	Oxalobacteraceae_unclassified	7.78E+00	(1.341591434;45.0841765)	2.22E-02	CD56hi (sqrt)	3.34E-01
43	Actinobacillus	2.79E-01	(.0933019224;.8341976131)	2.24E-02	CD56hi (sqrt)	3.34E-01
44	Enterococcaceae_unclassified	1.54E-01	(.0303706853;.7765769054)	2.35E-02	CD69+ (log)	3.34E-01

45	Alloprevotella	2.68E+00	(1.138164919;6.30822518)	2.41E-02	CD25+ (log)	3.34E-01
46	Actinobacillus	3.49E+00	(1.177605415;10.31821029)	2.41E-02	CD25+ CD56hi (log)	3.34E-01
47	Bradyrhizobium	1.97E-01	(.0473032402;.8196120758)	2.55E-02	CD69+ CD56hi (log)	3.34E-01
48	Solobacterium	3.16E+00	(1.145006525;8.734277156)	2.63E-02	CD25+ (log)	3.35E-01
49	Flavobacterium	6.79E+00	(1.240218467;37.12942187)	2.72E-02	CD56hi (sqrt)	3.40E-01
50	Sphingomonas	2.87E-01	(.0944733172;.869999209)	2.74E-02	CD69+ CD56hi (log)	3.40E-01
51	Actinobacillus	4.92E-01	(.2622678696;.9245596287)	2.75E-02	NKT CD8+ total (log)	3.40E-01
52	Micrococcaceae_unclassified	2.04E-01	(.0496238151;.8416196343)	2.79E-02	CD56hi (sqrt)	3.40E-01
53	Veillonellaceae_unclassified	2.43E+00	(1.080604413;5.444461879)	3.17E-02	CD25+ CD56lo (log)	3.66E-01
54	Prevotellaceae_unclassified	2.37E-01	(.0632430681;.8918687275)	3.32E-02	NK total (log)	3.76E-01
55	Enterococcaceae_unclassified	1.95E-01	(.04321663;.8791538125)	3.34E-02	CD69+ CD56lo (log)	3.76E-01
56	Planococcaceae_unclassified	1.70E-01	(.0331519194;.8707663493)	3.35E-02	NK total (log)	3.76E-01
57	Streptobacillus	1.97E+00	(1.050729586;3.679800661)	3.45E-02	NKT CD8+ total (log)	3.76E-01
58	Veillonella	4.25E-01	(.191736042;.9402007925)	3.47E-02	CD56lo (log)	3.76E-01
59	Prevotellaceae_unclassified	2.82E-01	(.0862295065;.9242309566)	3.66E-02	CD56lo (log)	3.79E-01
60	Tannerella	2.90E-01	(.0908432011;.926383816)	3.67E-02	CD69+ CD56hi (log)	3.79E-01
61	Planococcaceae_unclassified	2.00E-01	(.0435529126;.9187767499)	3.86E-02	CD56lo (log)	3.86E-01
62	Enterococcaceae_unclassified	2.85E+00	(1.04851791;7.759643233)	4.01E-02	CD56hi (sqrt)	3.95E-01
63	Selenomonas	3.39E-01	(.1207479628;.9538818618)	4.04E-02	CD69+ CD56hi (log)	3.95E-01
64	Oxalobacteraceae_unclassified	6.82E+00	(1.083846767;42.93862105)	4.08E-02	CD56hi (sqrt)	3.95E-01
65	Actinobacillus	2.33E-01	(.0569565168;.9525702919)	4.26E-02	CD56hi (sqrt)	4.00E-01
66	Parvimonas	8.74E+00	(1.072832253;71.17000758)	4.28E-02	CD56hi (sqrt)	4.00E-01
67	Catonella	3.92E-01	(.1573272342;.9790001756)	4.49E-02	CD69+ CD56hi (log)	4.10E-01
68	Capnocytophaga	2.24E+00	(1.01807085;4.918974337)	4.50E-02	NKT CD8+ total (log)	4.10E-01
69	Catonella	3.26E-01	(.1086614212;.9794437397)	4.58E-02	NK total (log)	4.10E-01
70	Prevotellaceae_ge	2.68E+00	(1.01457692;7.09280462)	4.67E-02	CD56hi (sqrt)	4.10E-01
71	Paucibacter	3.08E-01	(.0958847921;.9864334729)	4.74E-02	CD69+ CD56hi (log)	4.10E-01
72	Methylobacterium-Methylorubrum	3.31E+00	(1.011309387;10.85615396)	4.79E-02	CD25+ (log)	4.10E-01
73	Veillonella	7.86E-01	(.6186062183;.99801201)	4.81E-02	CD56lo (sqrt)	4.10E-01
74	Veillonellaceae_unclassified	3.34E+00	(.9996626235;11.12914404)	5.01E-02	CD25+ (log)	4.13E-01
75	Alloprevotella	1.91E+00	(.9976837916;3.673360557)	5.08E-02	NKT CD8+ total (log)	4.14E-01
76	Flavobacterium	6.19E+00	(.9779681502;39.15564627)	5.28E-02	CD56hi (sqrt)	4.25E-01
77	Veillonella	1.78E+00	(.9894638397;3.206800626)	5.43E-02	CD25+ (log)	4.31E-01
78	Planococcaceae_unclassified	6.68E-01	(.4417083485;1.0093511)	5.54E-02	CD56lo (sqrt)	4.34E-01
79	Catonella	3.39E-01	(.1113695659;1.028949236)	5.62E-02	CD56lo (log)	4.34E-01
80	Catonella	7.52E-01	(.5619058309;1.007728465)	5.64E-02	NK total (sqrt)	4.34E-01
81	Veillonella	1.78E+00	(.9839622483;3.227575521)	5.66E-02	NKT CD8+ total (log)	4.34E-01
82	Enterococcaceae_unclassified	3.56E-01	(.1218235927;1.037404274)	5.84E-02	NKT CD8+ total (log)	4.35E-01
83	Prevotellaceae_ge	3.93E-01	(.1495924907;1.034099661)	5.85E-02	CD69+ CD56hi (log)	4.35E-01
84	Paucibacter	3.77E-01	(.1369475089;1.036207893)	5.86E-02	NKT CD8+ total (log)	4.35E-01
85	Oxalobacteraceae_unclassified	9.63E-02	(.0084251648;1.099948696)	5.97E-02	CD25+ CD56hi (log)	4.35E-01
86	Veillonella	4.33E-01	(.181205564;1.036523879)	6.02E-02	NK total (log)	4.35E-01
87	Solobacterium	2.50E+00	(.9583720443;6.528164791)	6.11E-02	CD25+ CD56lo (log)	4.35E-01
88	Planococcaceae_unclassified	6.56E-01	(.4213766911;1.02037993)	6.14E-02	NK total (sqrt)	4.35E-01

89	Solobacterium	4.05E-01	(.1561473067;1.049806561)	6.29E-02	CD69+ CD56hi (log)	4.35E-01
90	Micrococcaceae_unclassified	1.65E-01	(.024447213;1.109111782)	6.38E-02	CD56hi (sqrt)	4.35E-01
91	Parvimonas	1.51E+00	(.9713075235;2.332014079)	6.73E-02	CD56lo (sqrt)	4.52E-01
92	Streptococcus	1.98E+00	(.9465875883;4.139511321)	6.97E-02	CD69+ CD56hi (log)	4.63E-01
93	Actinomyces	2.42E-01	(.0511342215;1.145558884)	7.37E-02	CD25+ CD56lo (log)	4.84E-01
94	Oribacterium	2.89E-01	(.0734592952;1.138583489)	7.60E-02	CD56hi (sqrt)	4.94E-01
95	Lautropia	3.64E+00	(.8658294316;15.26208938)	7.79E-02	CD25+ CD56hi (log)	5.01E-01
96	Treponema	1.58E-01	(.0202046099;1.231549415)	7.82E-02	CD69+ CD56hi (log)	5.01E-01
97	Alloprevotella	4.86E-01	(.2169849161;1.087382801)	7.91E-02	CD56lo (log)	5.01E-01
98	Micrococcaceae_unclassified	2.94E+00	(.8731705094;9.932336932)	8.16E-02	CD69+ (log)	5.09E-01
99	Fusobacterium	4.94E-01	(.2234474655;1.093176969)	8.19E-02	NK total (log)	5.09E-01
100	Capnocytophaga	2.22E+00	(.9032519405;5.458859997)	8.22E-02	CD56hi (sqrt)	5.09E-01
101	Parvimonas	4.45E+00	(.8189446217;24.14073262)	8.39E-02	CD56lo (log)	5.09E-01
102	Prevotellaceae_unclassified	7.22E-01	(.4970642835;1.047353845)	8.60E-02	NK total (sqrt)	5.15E-01
103	Alloprevotella	8.21E-01	(.6544326525;1.02900866)	8.68E-02	CD56lo (sqrt)	5.15E-01
104	Oribacterium	1.92E+00	(.9074946915;4.071571441)	8.79E-02	CD25+ (log)	5.17E-01
105	Catonella	7.69E-01	(.5689887726;1.04056522)	8.88E-02	CD56lo (sqrt)	5.17E-01
106	Veillonella	8.01E-01	(.6204377785;1.034304875)	8.89E-02	NK total (sqrt)	5.17E-01
107	Prevotellaceae_unclassified	7.40E-01	(.5219753172;1.047997312)	8.98E-02	CD56lo (sqrt)	5.17E-01
108	Micrococcaceae_unclassified	2.78E+00	(.8430496316;9.153992636)	9.31E-02	CD69+ CD56lo (log)	5.27E-01
109	Streptococcus	6.34E-01	(.3721196347;1.080753814)	9.40E-02	NKT CD8+ total (log)	5.27E-01
110	Comamonadaceae_unclassified	7.61E+00	(.7067760166;82.02629133)	9.42E-02	CD56hi (sqrt)	5.27E-01
111	Tannerella	1.93E+00	(.8834426665;4.200735057)	9.93E-02	NKT CD8+ total (log)	5.46E-01
112	Parvimonas	1.49E+00	(.9233054419;2.402682998)	1.02E-01	NK total (sqrt)	5.59E-01
113	Acinetobacter	1.67E-01	(.0189881168;1.469111852)	1.07E-01	CD56hi (sqrt)	5.77E-01
114	Capnocytophaga	2.02E+00	(.8578754683;4.768057538)	1.07E-01	CD56hi (sqrt)	5.77E-01
115	Treponema	4.34E-01	(.1563338145;1.203410536)	1.09E-01	CD25+ CD56lo (log)	5.77E-01
116	Corynebacterium	2.73E-01	(.0556530584;1.337834021)	1.09E-01	CD56hi (sqrt)	5.77E-01
117	Tannerella	1.64E+00	(.8940111531;3.022774038)	1.10E-01	CD25+ (log)	5.77E-01
118	Neisseria	3.81E-01	(.1164226465;1.249044764)	1.11E-01	CD25+ CD56hi (log)	5.77E-01
119	Weeksellaceae_unclassified	1.67E+00	(.8881008387;3.136200651)	1.12E-01	CD25+ (log)	5.77E-01
120	Comamonadaceae_unclassified	2.77E-01	(.0564899867;1.354752762)	1.13E-01	CD25+ CD56hi (log)	5.77E-01
121	Capnocytophaga	4.71E-01	(.1851812633;1.196186741)	1.13E-01	CD69+ (log)	5.77E-01
122	Alloprevotella	5.01E-01	(.2115580285;1.184133453)	1.15E-01	NK total (log)	5.77E-01
123	Alloprevotella	2.48E+00	(.7936604863;7.772227644)	1.18E-01	CD25+ CD56hi (log)	5.87E-01
124	Actinobacillus	2.45E+00	(.792275751;7.599536457)	1.20E-01	CD56lo (log)	5.90E-01
125	Parvimonas	4.37E+00	(.6773359757;28.22500237)	1.21E-01	NK total (log)	5.92E-01
126	Fusobacterium	5.41E-01	(.2483470081;1.17792648)	1.22E-01	CD56lo (log)	5.92E-01
127	Neisseria	1.87E+00	(.8448375933;4.140829523)	1.23E-01	NKT CD8+ total (log)	5.92E-01
128	Terrisporobacter	2.36E+00	(.7830114041;7.084340845)	1.27E-01	NKT CD8+ total (log)	6.08E-01
129	Campylobacter	4.20E-01	(.1374359412;1.283019787)	1.28E-01	CD69+ (log)	6.08E-01
130	Actinobacillus	5.08E-01	(.2120696504;1.216554146)	1.28E-01	CD25+ CD56lo (log)	6.08E-01
131	Veillonella	1.94E+00	(.8233658975;4.567135576)	1.30E-01	CD25+ CD56lo (log)	6.08E-01
132	Cardiobacterium	1.34E+00	(.9129721146;1.968697947)	1.35E-01	CD56lo (sqrt)	6.24E-01
133	Corynebacterium	2.15E+00	(.7878504274;5.842413115)	1.35E-01	CD25+ CD56lo (log)	6.24E-01
134	Actinobacillus	2.50E+00	(.7487681965;8.378428699)	1.36E-01	NK total (log)	6.24E-01
135	Enterococcaceae_unclassified	1.94E+00	(.8088336562;4.671984158)	1.37E-01	CD56lo (log)	6.24E-01
136	Enterococcaceae_unclassified	2.38E+00	(.7583056645;7.45466388)	1.37E-01	CD56hi (sqrt)	6.24E-01
137	Cardiobacterium	3.20E+00	(.6870664605;14.93916557)	1.38E-01	CD56lo (log)	6.24E-01

138	Prevotellaceae_ge	1.73E+00	(.8366213948;3.581147631)	1.39E-01	NKT CD8+ total (log)	6.24E-01
139	Fusobacterium	8.50E-01	(.6852635297;1.05481114)	1.40E-01	NK total (sqrt)	6.24E-01
140	Rothia	2.46E+00	(.7348696261;8.25752751)	1.44E-01	CD56hi (sqrt)	6.29E-01
141	Methylobacterium-Methylorubrum	3.09E+00	(.6784192278;14.07200844)	1.45E-01	CD25+ CD56lo (log)	6.29E-01
142	Capnocytophaga	4.97E-01	(.1929864275;1.280891128)	1.48E-01	CD69+ CD56lo (log)	6.36E-01
143	Acinetobacter	3.01E-01	(.0586103446;1.543132053)	1.50E-01	CD25+ (log)	6.40E-01
144	Bradyrhizobium	4.10E+00	(.6002625651;27.97757354)	1.50E-01	CD25+ CD56lo (log)	6.40E-01
145	Lachnospiraceae_unclassified	3.31E-01	(.0729763285;1.499865757)	1.51E-01	CD56hi (sqrt)	6.40E-01
146	Unclassified	1.43E+00	(.8757083347;2.345914781)	1.52E-01	CD25+ (log)	6.40E-01
147	Prevotella	1.60E+00	(.8398922197;3.062981658)	1.52E-01	CD25+ (log)	6.40E-01
148	Actinobacillus	2.12E+00	(.7573660274;5.917790636)	1.53E-01	CD69+ CD56hi (log)	6.40E-01
149	Acinetobacter	3.71E-01	(.0951055625;1.448833055)	1.54E-01	CD69+ (log)	6.40E-01
150	Campylobacter	2.14E+00	(.7339009362;6.228323731)	1.64E-01	CD25+ CD56hi (log)	6.67E-01
151	Prevotellaceae_unclassified	2.43E+00	(.6955291442;8.517560558)	1.64E-01	CD56hi (sqrt)	6.67E-01
152	Corynebacterium	3.91E-01	(.1035288768;1.476581419)	1.66E-01	CD56hi (sqrt)	6.67E-01
153	Actinomyces	1.54E-01	(.0108015119;2.20092487)	1.68E-01	CD25+ CD56hi (log)	6.71E-01
154	Actinobacillus	1.23E+00	(.9150507757;1.65372178)	1.70E-01	CD56lo (sqrt)	6.75E-01
155	Parvimonas	5.22E+00	(.4827906092;56.34101577)	1.74E-01	CD56hi (sqrt)	6.85E-01
156	Parvimonas	3.09E-01	(.055457989;1.720029767)	1.80E-01	CD69+ (log)	7.05E-01
157	Alloprevotella	8.46E-01	(.6611984224;1.081641138)	1.82E-01	NK total (sqrt)	7.08E-01
158	Actinobacillus	1.23E+00	(.9065210396;1.677328238)	1.82E-01	NK total (sqrt)	7.08E-01
159	Tannerella	2.30E+00	(.6722377073;7.858411988)	1.85E-01	CD25+ CD56hi (log)	7.09E-01
160	Streptobacillus	2.10E+00	(.6996529584;6.285924354)	1.86E-01	CD25+ CD56hi (log)	7.11E-01
161	Rothia	1.19E+00	(.9196542159;1.537623678)	1.86E-01	NK total (sqrt)	7.11E-01
162	Cardiobacterium	3.00E+00	(.5862765816;15.30302244)	1.87E-01	NK total (log)	7.11E-01
163	Terrisporobacter	2.93E-01	(.0470683177;1.822197053)	1.88E-01	CD69+ CD56hi (log)	7.11E-01
164	Rothia	1.17E+00	(.9218698935;1.496258745)	1.93E-01	CD56lo (sqrt)	7.19E-01
165	Planococcaceae_unclassified	2.03E+00	(.6892145939;5.967176235)	1.99E-01	CD69+ CD56lo (log)	7.37E-01
166	Weeksellaceae_unclassified	6.79E-01	(.373658056;1.235043768)	2.05E-01	NKT CD8+ total (log)	7.54E-01
167	Treponema	3.07E-01	(.0488794441;1.922123952)	2.07E-01	CD56hi (sqrt)	7.57E-01
168	Alloprevotella	5.15E-01	(.1839035011;1.444554794)	2.07E-01	CD69+ CD56hi (log)	7.57E-01
169	Rothia	1.84E+00	(.712493301;4.731176418)	2.08E-01	CD56lo (log)	7.57E-01
170	Streptobacillus	5.65E-01	(.2321842977;1.375172393)	2.08E-01	CD69+ CD56hi (log)	7.57E-01
171	Rothia	1.95E+00	(.6881073034;5.529732641)	2.09E-01	NK total (log)	7.57E-01
172	Cardiobacterium	1.30E+00	(.8651430577;1.939224386)	2.09E-01	NK total (sqrt)	7.57E-01
173	Parvimonas	3.28E-01	(.0572250144;1.879489359)	2.11E-01	CD69+ CD56hi (log)	7.57E-01
174	Fusobacterium	8.68E-01	(.6940937049;1.084572178)	2.12E-01	CD56lo (sqrt)	7.57E-01
175	Corynebacterium	5.15E-01	(.181087198;1.467250233)	2.14E-01	CD25+ CD56hi (log)	7.57E-01
176	Corynebacterium	5.34E-01	(.1985609056;1.437502719)	2.14E-01	CD69+ CD56hi (log)	7.57E-01
177	Tannerella	4.32E-01	(.1147731999;1.626348042)	2.15E-01	CD56hi (sqrt)	7.57E-01
178	Oribacterium	2.20E+00	(.6329394626;7.626348134)	2.15E-01	CD69+ CD56lo (log)	7.57E-01
179	Streptococcus	5.93E-01	(.2593224486;1.355246201)	2.15E-01	CD25+ CD56hi (log)	7.57E-01
180	Bradyrhizobium	4.53E-01	(.1259742395;1.626748512)	2.25E-01	CD56lo (log)	7.62E-01
181	Parvimonas	4.51E-01	(.1248481143;1.631638522)	2.25E-01	CD25+ (log)	7.62E-01
182	Prevotellaceae_unclassified	2.23E+00	(.6093985962;8.13716908)	2.26E-01	CD69+ CD56lo (log)	7.62E-01
183	Enterococcaceae_unclassified	5.75E-01	(.2341991878;1.412993658)	2.28E-01	CD25+ (log)	7.62E-01
184	Prevotella	5.87E-01	(.245431818;1.401687425)	2.30E-01	CD56lo (log)	7.64E-01
185	Prevotella	5.68E-01	(.225790872;1.431310015)	2.31E-01	NK total (log)	7.64E-01
186	Lautropia	4.65E-01	(.13144541;1.643005194)	2.34E-01	CD56lo (log)	7.70E-01
187	Acinetobacter	2.80E-01	(.033669623;2.325392151)	2.38E-01	NKT CD8+ total (log)	7.79E-01
188	Treponema	4.14E-01	(.0948352851;1.808711506)	2.41E-01	CD56hi (sqrt)	7.84E-01
189	Paucibacter	1.86E-01	(.0111374082;3.105585789)	2.42E-01	CD25+ CD56hi (log)	7.84E-01

190	Planococcaceae_unclassified	5.66E-01	(.2168124751;1.477641332)	2.45E-01	NKT CD8+ total (log)	7.88E-01
191	Planococcaceae_unclassified	4.90E-01	(.146683541;1.63781088)	2.47E-01	CD25+ CD56hi (log)	7.89E-01
192	Prevotella	5.85E-01	(.2331906697;1.468312463)	2.54E-01	CD69+ CD56hi (log)	8.07E-01
193	Porphyromonas	1.78E+00	(.6577481981;4.837280303)	2.56E-01	NKT CD8+ total (log)	8.09E-01
194	Parvimonas	2.39E+00	(.5320895353;10.70747276)	2.56E-01	CD25+ CD56hi (log)	8.09E-01
195	Acinetobacter	2.98E-01	(.0367658712;2.408721129)	2.56E-01	CD25+ CD56lo (log)	8.09E-01
196	Lautropia	8.16E-01	(.5744042857;1.159622725)	2.57E-01	CD56lo (sqrt)	8.09E-01
197	Alloprevotella	2.01E+00	(.5958663698;6.799638724)	2.60E-01	CD56hi (sqrt)	8.09E-01
198	Prevotella	2.02E+00	(.5912640102;6.883806418)	2.62E-01	CD25+ CD56hi (log)	8.10E-01
199	Cardiobacterium	4.08E-01	(.0847692841;1.966645511)	2.64E-01	CD69+ CD56hi (log)	8.11E-01
200	Campylobacter	5.33E-01	(.1754090144;1.617973061)	2.67E-01	CD69+ CD56lo (log)	8.14E-01
201	Bradyrhizobium	4.52E-01	(.1113554928;1.838352141)	2.68E-01	NK total (log)	8.14E-01
202	Alloprevotella	1.88E+00	(.6155028542;5.739941332)	2.68E-01	CD25+ CD56lo (log)	8.14E-01
203	Veillonellaceae_unclassified	2.46E+00	(.4994269474;12.07975203)	2.69E-01	CD69+ CD56lo (log)	8.14E-01
204	Neisseria	1.95E+00	(.5916403166;6.439172063)	2.72E-01	CD56hi (sqrt)	8.15E-01
205	Catonella	4.36E-01	(.0980217293;1.935965469)	2.75E-01	CD25+ CD56hi (log)	8.19E-01
206	Bradyrhizobium	8.16E-01	(.5663994565;1.176155581)	2.76E-01	CD56lo (sqrt)	8.19E-01
207	Neisseriaceae_unclassified	1.84E+00	(.611575622;5.542382745)	2.78E-01	CD25+ CD56hi (log)	8.20E-01
208	Selenomonas	4.93E-01	(.1376213073;1.767949308)	2.78E-01	CD25+ CD56hi (log)	8.20E-01
209	Lachnospiraceae_unclassified	3.54E-01	(.0541836232;2.31571256)	2.79E-01	CD56hi (sqrt)	8.20E-01
210	Sphingomonas	1.70E+00	(.6518155388;4.414124865)	2.79E-01	CD25+ (log)	8.20E-01
211	Tannerella	4.39E-01	(.0981130996;1.965572767)	2.82E-01	CD69+ (log)	8.20E-01
212	Veillonellaceae_unclassified	2.55E+00	(.4639343257;13.98650821)	2.82E-01	CD69+ (log)	8.20E-01
213	Parvimonas	4.20E-01	(.0863233822;2.041494013)	2.82E-01	CD69+ CD56lo (log)	8.20E-01
214	Solobacterium	1.67E+00	(.6529932027;4.294182372)	2.83E-01	NKT CD8+ total (log)	8.20E-01
215	Prevotellaceae_ge	5.79E-01	(.2137312139;1.570377702)	2.83E-01	CD69+ (log)	8.20E-01
216	Pasteurellaceae_unclassified	5.45E-01	(.1794553964;1.654746716)	2.84E-01	CD25+ CD56lo (log)	8.20E-01
217	Lautropia	2.06E+00	(.5410357443;7.836105203)	2.90E-01	CD25+ CD56lo (log)	8.20E-01
218	Lachnospiraceae_unclassified	1.95E+00	(.5652939897;6.702932692)	2.91E-01	CD25+ CD56hi (log)	8.20E-01
219	Atopobium	2.96E+00	(.3944102857;22.16737811)	2.92E-01	CD56hi (sqrt)	8.20E-01
220	Enterococcaceae_unclassified	1.72E+00	(.6248759957;4.742134932)	2.93E-01	NK total (log)	8.20E-01
221	Lautropia	4.22E-01	(.084231699;2.111948275)	2.94E-01	CD56hi (sqrt)	8.20E-01
222	Lautropia	1.54E+00	(.6841357496;3.485534774)	2.96E-01	NKT CD8+ total (log)	8.20E-01
223	Unclassified	6.57E-01	(.2979496779;1.44663147)	2.97E-01	NK total (log)	8.20E-01
224	Unclassified	6.85E-01	(.3356449565;1.396115723)	2.97E-01	CD56lo (log)	8.20E-01
225	Lautropia	4.85E-01	(.1232228972;1.906112305)	3.00E-01	NK total (log)	8.20E-01
226	Neisseria	6.52E-01	(.2903000853;1.465791658)	3.01E-01	CD69+ CD56hi (log)	8.20E-01
227	Pasteurellaceae_unclassified	1.63E-01	(.0051772149;5.145269761)	3.03E-01	CD56hi (sqrt)	8.20E-01
228	Unclassified	7.35E-01	(.4087620364;1.322744977)	3.05E-01	NKT CD8+ total (log)	8.20E-01
229	Atopobium	2.10E+00	(.5063418429;8.690418767)	3.07E-01	CD69+ (log)	8.20E-01
230	Neisseria	1.98E+00	(.5261062358;7.438070308)	3.13E-01	CD56hi (sqrt)	8.31E-01
231	Corynebacterium	5.98E-01	(.2181973278;1.639473644)	3.18E-01	NK total (log)	8.41E-01
232	Alysiella	5.41E-01	(.1621455756;1.808061198)	3.19E-01	CD69+ CD56hi (log)	8.41E-01
233	Atopobium	2.25E+00	(.4511407759;11.18537693)	3.23E-01	CD25+ CD56hi (log)	8.47E-01
234	Veillonella	1.86E+00	(.5428776919;6.344284462)	3.24E-01	CD56hi (sqrt)	8.47E-01
235	Corynebacterium	8.76E-01	(.6722569517;1.142024261)	3.28E-01	NK total (sqrt)	8.54E-01
236	Acinetobacter	5.24E-01	(.1415065716;1.937762895)	3.33E-01	CD69+ CD56lo (log)	8.61E-01
237	Lautropia	8.28E-01	(.5649459687;1.213504086)	3.33E-01	NK total (sqrt)	8.61E-01
238	Sphingomonas	5.47E-01	(.1612509728;1.858615328)	3.34E-01	NK total (log)	8.61E-01

239	Alloprevotella	1.86E+00	(.5280071505;6.519361553)	3.35E-01	CD56hi (sqrt)	8.61E-01
240	Atopobium	1.93E+00	(.5059176624;7.368202076)	3.36E-01	CD69+ CD56lo (log)	8.61E-01
241	Terrisporobacter	4.03E-01	(.0631708496;2.573811513)	3.37E-01	CD56hi (sqrt)	8.61E-01
242	Flavobacterium	6.11E-01	(.2229833313;1.674318582)	3.38E-01	NKT CD8+ total (log)	8.61E-01
243	Weeksellaceae_unclassified	6.24E-01	(.2364116061;1.647471567)	3.41E-01	CD69+ (log)	8.61E-01
244	Prevotellaceae_ge	5.70E-01	(.1790412452;1.816005333)	3.42E-01	CD25+ CD56hi (log)	8.61E-01
245	Veillonella	1.77E+00	(.5456818376;5.716098568)	3.43E-01	CD56hi (sqrt)	8.61E-01
246	Flavobacterium	4.13E-01	(.066005479;2.586417054)	3.45E-01	CD25+ CD56hi (log)	8.61E-01
247	Terrisporobacter	2.49E+00	(.3721842469;16.70771276)	3.46E-01	CD69+ CD56lo (log)	8.61E-01
248	Haemophilus	4.76E-01	(.1011643517;2.236130751)	3.47E-01	CD69+ (log)	8.61E-01
249	Solobacterium	2.21E+00	(.4239378039;11.4818622)	3.47E-01	CD69+ CD56lo (log)	8.61E-01
250	Oxalobacteraceae_unclassified	2.13E+00	(.4400689921;10.27701051)	3.48E-01	CD25+ (log)	8.61E-01
251	Sphingomonas	5.85E-01	(.1907372235;1.7940191)	3.48E-01	CD56lo (log)	8.61E-01
252	Prevotellaceae_unclassified	1.56E+00	(.6119904714;3.995553287)	3.50E-01	NKT CD8+ total (log)	8.61E-01
253	Prevotella	1.77E+00	(.5346022806;5.828389295)	3.51E-01	CD69+ CD56lo (log)	8.61E-01
254	Enterococcaceae_unclassified	4.29E-01	(.0713437385;2.575373987)	3.54E-01	CD69+ CD56hi (log)	8.61E-01
255	Catonella	1.49E+00	(.6381262201;3.499931924)	3.55E-01	NKT CD8+ total (log)	8.61E-01
256	Paucibacter	5.84E-01	(.1864533348;1.827966768)	3.55E-01	NK total (log)	8.61E-01
257	Neisseriaceae_unclassified	5.54E-01	(.1582269384;1.942676946)	3.57E-01	CD69+ (log)	8.61E-01
258	Bradyrhizobium	8.27E-01	(.5525606149;1.238734721)	3.57E-01	NK total (sqrt)	8.61E-01
259	Oribacterium	1.88E+00	(.4871818729;7.281764634)	3.59E-01	CD69+ (log)	8.61E-01
260	Tannerella	5.12E-01	(.1216163898;2.156460511)	3.62E-01	CD69+ CD56lo (log)	8.61E-01
261	Cardiobacterium	5.76E-01	(.1750882654;1.895456429)	3.64E-01	CD25+ CD56lo (log)	8.61E-01
262	Rothia	6.67E-01	(.2773792475;1.606048516)	3.67E-01	CD69+ CD56hi (log)	8.61E-01
263	Paucibacter	5.93E-01	(.1906252329;1.845690474)	3.67E-01	CD69+ (log)	8.61E-01
264	Neisseriaceae_unclassified	1.55E+00	(.5983559233;4.009476124)	3.67E-01	CD25+ (log)	8.61E-01
265	Alysiella	1.69E+00	(.5370527991;5.336592009)	3.69E-01	NKT CD8+ total (log)	8.61E-01
266	Neisseriaceae_unclassified	5.66E-01	(.1635549041;1.958478706)	3.69E-01	CD69+ CD56lo (log)	8.61E-01
267	Prevotellaceae_unclassified	1.82E+00	(.4928726551;6.698239971)	3.70E-01	CD25+ CD56lo (log)	8.61E-01
268	Unclassified	1.69E+00	(.5370917254;5.290968854)	3.71E-01	CD56hi (sqrt)	8.61E-01
269	Neisseriaceae_unclassified	5.68E-01	(.1641427334;1.962550129)	3.71E-01	NK total (log)	8.61E-01
270	Prevotella	8.91E-01	(.6918913138;1.147386842)	3.71E-01	CD56lo (sqrt)	8.61E-01
271	Oxalobacteraceae_unclassified	5.49E-01	(.1472525167;2.046309179)	3.72E-01	NKT CD8+ total (log)	8.61E-01
272	Pasteurellaceae_unclassified	6.39E-01	(.2379894561;1.714170322)	3.73E-01	CD69+ CD56hi (log)	8.61E-01
273	Planococcaceae_unclassified	4.85E-01	(.0984577264;2.387983274)	3.74E-01	CD69+ CD56hi (log)	8.61E-01
274	Treponema	2.94E-01	(.0197199563;4.373377057)	3.74E-01	NK total (log)	8.61E-01
275	Prevotella	8.89E-01	(.6856376887;1.153083412)	3.76E-01	NK total (sqrt)	8.61E-01
276	Neisseriaceae_unclassified	5.00E-01	(.1057651408;2.360171542)	3.81E-01	CD56hi (sqrt)	8.61E-01
277	Porphyromonas	5.81E-01	(.1723185372;1.959399255)	3.81E-01	CD69+ (log)	8.61E-01
278	Selenomonas	6.74E-01	(.2783852943;1.632749715)	3.82E-01	CD25+ (log)	8.61E-01
279	Paucibacter	8.57E-01	(.6057881381;1.212673644)	3.84E-01	NK total (sqrt)	8.61E-01
280	Paucibacter	6.23E-01	(.2134798889;1.82056445)	3.87E-01	CD56lo (log)	8.61E-01
281	Neisseria	1.27E+00	(.7377222445;2.189241129)	3.88E-01	CD25+ (log)	8.61E-01
282	Solobacterium	1.92E+00	(.4339434682;8.529170914)	3.89E-01	CD56hi (sqrt)	8.61E-01
283	Sphingomonas	8.68E-01	(.6275471445;1.201802979)	3.95E-01	CD56lo (sqrt)	8.61E-01
284	Capnocytophaga	1.43E+00	(.6267337863;3.250918615)	3.97E-01	CD25+ (log)	8.61E-01

285	Sphingomonas	8.58E-01	(.6003383055;1.225439315)	3.99E-01	NK total (sqrt)	8.61E-01
286	Prevotellaceae_ge	9.05E-01	(.718043193;1.141522343)	4.00E-01	CD56lo (sqrt)	8.61E-01
287	Corynebacterium	8.95E-01	(.6889597861;1.161979617)	4.04E-01	CD56lo (sqrt)	8.61E-01
288	Prevotellaceae_ge	7.00E-01	(.301443696;1.623527169)	4.06E-01	CD56lo (log)	8.61E-01
289	Corynebacterium	6.67E-01	(.2561380006;1.734596121)	4.06E-01	CD56lo (log)	8.61E-01
290	Selenomonas	1.67E+00	(.4963300138;5.600886635)	4.08E-01	CD25+ CD56lo (log)	8.61E-01
291	Paucibacter	8.74E-01	(.6344800613;1.204672213)	4.11E-01	CD56lo (sqrt)	8.64E-01
292	Lachnospiraceae_unclassified	1.32E+00	(.6812139267;2.550547822)	4.12E-01	CD25+ (log)	8.64E-01
293	Neisseriaceae_unclassified	6.64E-01	(.250019628;1.76610072)	4.12E-01	NKT CD8+ total (log)	8.64E-01
294	Porphyromonas	6.58E-01	(.2399535947;1.804927262)	4.16E-01	NK total (log)	8.65E-01
295	Haemophilus	5.91E-01	(.1653052232;2.110679491)	4.18E-01	NKT CD8+ total (log)	8.65E-01
296	Comamonadaceae_unclassified	1.22E+00	(.7497658906;1.998183975)	4.19E-01	CD56lo (sqrt)	8.65E-01
297	Lachnoanaerobaculum	1.28E+00	(.7057008369;2.306825627)	4.20E-01	NKT CD8+ total (log)	8.65E-01
298	Neisseriaceae_unclassified	6.19E-01	(.1918673118;1.994488934)	4.21E-01	CD56lo (log)	8.65E-01
299	Actinomyces	6.50E-01	(.2271322803;1.861979063)	4.23E-01	CD69+ (log)	8.65E-01
300	Weeksellaceae_unclassified	1.37E+00	(.6320122899;2.96647981)	4.26E-01	CD56lo (log)	8.67E-01
301	Prevotellaceae_ge	6.92E-01	(.2802294889;1.711266255)	4.26E-01	NK total (log)	8.67E-01
302	Oribacterium	4.99E-01	(.0893093154;2.787007977)	4.28E-01	CD25+ CD56hi (log)	8.67E-01
303	Comamonadaceae_unclassified	6.66E-01	(.2434209598;1.823122527)	4.29E-01	CD25+ (log)	8.67E-01
304	Terrisporobacter	1.98E+00	(.3626455724;10.82498059)	4.30E-01	CD25+ CD56hi (log)	8.67E-01
305	Corynebacterium	1.55E+00	(.5113362183;4.709594166)	4.38E-01	NKT CD8+ total (log)	8.77E-01
306	Prevotellaceae_unclassified	1.62E+00	(.4779666333;5.494008251)	4.38E-01	CD69+ (log)	8.77E-01
307	Haemophilus	5.44E-01	(.1160512101;2.552439758)	4.40E-01	CD69+ CD56lo (log)	8.77E-01
308	Selenomonas	9.11E-01	(.7176277381;1.155830231)	4.42E-01	CD56lo (sqrt)	8.77E-01
309	Lachnoanaerobaculum	7.11E-01	(.2967490593;1.702827559)	4.44E-01	CD69+ CD56lo (log)	8.78E-01
310	Actinomyces	4.67E-01	(.0657694307;3.310649916)	4.46E-01	CD56hi (sqrt)	8.79E-01
311	Catonella	1.63E+00	(.4620081125;5.781426387)	4.46E-01	CD56hi (sqrt)	8.79E-01
312	Solobacterium	2.02E+00	(.3273345757;12.48824398)	4.49E-01	CD69+ (log)	8.79E-01
313	Planococcaceae_unclassified	1.37E+00	(.6075887934;3.079416178)	4.49E-01	CD25+ (log)	8.79E-01
314	Comamonadaceae_unclassified	1.22E+00	(.7295470047;2.036139371)	4.50E-01	NK total (sqrt)	8.79E-01
315	Comamonadaceae_unclassified	3.75E-01	(.0292549934;4.817219867)	4.52E-01	CD25+ CD56lo (log)	8.79E-01
316	Prevotellaceae_ge	9.09E-01	(.7097250182;1.165010385)	4.52E-01	NK total (sqrt)	8.79E-01
317	Unclassified	9.25E-01	(.755596569;1.133211702)	4.53E-01	CD56lo (sqrt)	8.79E-01
318	Prevotella	1.62E+00	(.457242877;5.742606684)	4.55E-01	CD69+ (log)	8.79E-01
319	Neisseria	9.11E-01	(.7141789608;1.162674478)	4.55E-01	CD56lo (sqrt)	8.79E-01
320	Neisseriaceae_unclassified	1.37E+00	(.59805552;3.150386433)	4.55E-01	CD69+ CD56hi (log)	8.79E-01
321	Oxalobacteraceae_unclassified	1.95E+00	(.3321998931;11.49674989)	4.59E-01	CD25+ CD56lo (log)	8.79E-01
322	Terrisporobacter	6.73E-01	(.2356626257;1.921988416)	4.60E-01	CD25+ CD56lo (log)	8.79E-01
323	Tannerella	5.87E-01	(.1427076124;2.417720786)	4.61E-01	CD56hi (sqrt)	8.79E-01
324	Unclassified	9.22E-01	(.741334641;1.146016885)	4.63E-01	NK total (sqrt)	8.79E-01
325	Fusobacterium	1.26E+00	(.6793946591;2.333436032)	4.64E-01	NKT CD8+ total (log)	8.79E-01
326	Treponema	4.20E-01	(.0403719741;4.371446367)	4.68E-01	CD56lo (log)	8.79E-01
327	Paucibacter	1.94E+00	(.3222620721;11.71559794)	4.69E-01	CD56hi (sqrt)	8.79E-01
328	Enterococcaceae_unclassified	2.53E+00	(.2037621952;31.49566092)	4.70E-01	CD25+ CD56hi (log)	8.79E-01

329	Pasteurellaceae_unclassified	3.81E-01	(.0274310755;5.278754576)	4.71E-01	CD56hi (sqrt)	8.79E-01
330	Porphyromonas	1.28E+00	(.6483594588;2.5233642)	4.78E-01	CD25+ CD56lo (log)	8.84E-01
331	Weeksellaceae_unclassified	7.23E-01	(.2953003955;1.772523226)	4.79E-01	CD69+ CD56lo (log)	8.84E-01
332	Bradyrhizobium	2.04E+00	(.2837610083;14.6421308)	4.79E-01	CD56hi (sqrt)	8.84E-01
333	Terrisporobacter	5.53E-01	(.1067806515;2.8610362)	4.80E-01	CD56hi (sqrt)	8.84E-01
334	Haemophilus	1.38E+00	(.5643840387;3.376393323)	4.80E-01	CD69+ CD56hi (log)	8.84E-01
335	Alysiella	7.14E-01	(.2801118875;1.819154769)	4.80E-01	CD25+ (log)	8.84E-01
336	Atopobium	2.16E+00	(.2532111641;18.41742429)	4.81E-01	CD56hi (sqrt)	8.84E-01
337	Weeksellaceae_unclassified	5.22E-01	(.0851273569;3.195102868)	4.81E-01	CD25+ CD56hi (log)	8.84E-01
338	Terrisporobacter	2.11E+00	(.2644520646;16.78516351)	4.82E-01	CD69+ (log)	8.84E-01
339	Oribacterium	1.41E+00	(.5391356351;3.6769969)	4.85E-01	NKT CD8+ total (log)	8.84E-01
340	Porphyromonas	1.24E+00	(.6757791157;2.275433738)	4.87E-01	CD25+ (log)	8.84E-01
341	Prevotellaceae_ge	6.96E-01	(.249139902;1.94418768)	4.89E-01	CD69+ CD56lo (log)	8.84E-01
342	Atopobium	1.28E+00	(.6335447713;2.594011928)	4.90E-01	CD25+ (log)	8.84E-01
343	Lachnoanaerobaculum	2.10E+00	(.2548888138;17.27817883)	4.91E-01	CD56hi (sqrt)	8.84E-01
344	Rothia	7.18E-01	(.2793240553;1.845244212)	4.91E-01	NKT CD8+ total (log)	8.84E-01
345	Neisseriaceae_unclassified	5.34E-01	(.0880492503;3.240259379)	4.95E-01	CD56hi (sqrt)	8.84E-01
346	Haemophilus	1.60E+00	(.4085148999;6.285146981)	4.99E-01	CD56hi (sqrt)	8.84E-01
347	Planococcaceae_unclassified	1.54E+00	(.4308241017;5.533637271)	5.05E-01	CD69+ (log)	8.89E-01
348	Methylobacterium-Methylorubrum	3.80E-01	(.0220028772;6.574033176)	5.06E-01	CD25+ CD56hi (log)	8.89E-01
349	Tannerella	6.55E-01	(.1878565858;2.285935681)	5.07E-01	NK total (log)	8.89E-01
350	Neisseria	7.22E-01	(.2757256811;1.891405876)	5.08E-01	CD56lo (log)	8.89E-01
351	Parvimonas	3.56E-01	(.0166172943;7.60542333)	5.08E-01	CD25+ CD56lo (log)	8.89E-01
352	Fusobacterium	1.45E+00	(.4842881174;4.319167637)	5.09E-01	CD25+ CD56hi (log)	8.89E-01
353	Neisseriaceae_unclassified	8.94E-01	(.639018957;1.25115188)	5.14E-01	NK total (sqrt)	8.90E-01
354	Rothia	7.80E-01	(.3690282424;1.648061952)	5.15E-01	CD25+ (log)	8.90E-01
355	Prevotellaceae_unclassified	1.34E+00	(.5509014917;3.283612494)	5.15E-01	CD25+ (log)	8.90E-01
356	Porphyromonas	9.16E-01	(.7026980488;1.194182276)	5.17E-01	NK total (sqrt)	8.90E-01
357	Selenomonas	6.37E-01	(.1623643542;2.496645335)	5.17E-01	CD69+ (log)	8.90E-01
358	Porphyromonas	9.23E-01	(.7212010112;1.180584099)	5.22E-01	CD56lo (sqrt)	8.92E-01
359	Solobacterium	1.70E+00	(.3333913032;8.69140006)	5.23E-01	CD56hi (sqrt)	8.92E-01
360	Tannerella	7.33E-01	(.2829964757;1.900175441)	5.23E-01	CD25+ CD56lo (log)	8.92E-01
361	Treponema	7.81E-01	(.3642276111;1.673115378)	5.24E-01	NK total (sqrt)	8.92E-01
362	Paucibacter	7.07E-01	(.2421753326;2.063253983)	5.26E-01	CD69+ CD56lo (log)	8.92E-01
363	Porphyromonas	7.45E-01	(.2971592123;1.869357112)	5.31E-01	CD56lo (log)	8.94E-01
364	Micrococcaceae_unclassified	1.39E+00	(.49364882;3.928064029)	5.31E-01	NKT CD8+ total (log)	8.94E-01
365	Pasteurellaceae_unclassified	8.63E-01	(.5442956666;1.369277994)	5.32E-01	NK total (sqrt)	8.94E-01
366	Tannerella	6.99E-01	(.2259356256;2.163583843)	5.35E-01	CD56lo (log)	8.94E-01
367	Selenomonas	1.59E+00	(.3664124797;6.902518586)	5.36E-01	CD56hi (sqrt)	8.94E-01
368	Unclassified	1.50E+00	(.414020868;5.434602526)	5.37E-01	CD56hi (sqrt)	8.94E-01
369	Bradyrhizobium	6.07E-01	(.1235082921;2.978358387)	5.38E-01	CD56hi (sqrt)	8.94E-01
370	Catonella	1.26E+00	(.6028482679;2.636962495)	5.38E-01	CD25+ (log)	8.94E-01
371	Porphyromonas	6.89E-01	(.2089056689;2.27038254)	5.40E-01	CD69+ CD56lo (log)	8.94E-01
372	Prevotellaceae_unclassified	1.48E+00	(.422673253;5.16361634)	5.41E-01	CD56hi (sqrt)	8.94E-01
373	Micrococcaceae_unclassified	9.02E-01	(.647914598;1.256695213)	5.43E-01	NK total (sqrt)	8.94E-01

374	Pasteurellaceae_unclassified	8.77E-01	(.5732255322;1.341211795)	5.44E-01	CD56lo (sqrt)	8.94E-01
375	Weeksellaceae_unclassified	1.30E+00	(.5531118308;3.069373387)	5.45E-01	NK total (log)	8.94E-01
376	Neisseriaceae_unclassified	1.46E+00	(.4227940928;5.059723409)	5.48E-01	CD25+ CD56lo (log)	8.94E-01
377	Enterococcaceae_unclassified	1.08E+00	(.8410893247;1.383958455)	5.50E-01	CD56lo (sqrt)	8.94E-01
378	Selenomonas	9.29E-01	(.7273382646;1.187379053)	5.58E-01	NK total (sqrt)	9.01E-01
379	Weeksellaceae_unclassified	1.07E+00	(.8575605487;1.326546007)	5.62E-01	CD56lo (sqrt)	9.07E-01
380	Enterococcaceae_unclassified	1.08E+00	(.8298078601;1.408518897)	5.63E-01	NK total (sqrt)	9.07E-01
381	Pasteurellaceae_unclassified	5.86E-01	(.0953677321;3.596703425)	5.63E-01	NK total (log)	9.07E-01
382	Atopobium	6.83E-01	(.1867224065;2.500884848)	5.65E-01	CD69+ CD56hi (log)	9.07E-01
383	Flavobacterium	1.38E+00	(.4543388845;4.203854872)	5.69E-01	CD25+ (log)	9.07E-01
384	Sphingomonas	6.02E-01	(.1052353297;3.448216226)	5.69E-01	CD56hi (sqrt)	9.07E-01
385	Haemophilus	9.07E-01	(.6472589276;1.270289969)	5.69E-01	CD56lo (sqrt)	9.07E-01
386	Haemophilus	1.64E+00	(.2977878087;8.997092218)	5.71E-01	CD56hi (sqrt)	9.07E-01
387	Lachnoanaerobaculum	1.25E+00	(.5808994063;2.675249773)	5.72E-01	CD25+ (log)	9.07E-01
388	Comamonadaceae_unclassified	1.76E+00	(.2446030077;12.67041532)	5.74E-01	NK total (log)	9.07E-01
389	Bradyrhizobium	1.43E+00	(.4090829956;4.964609825)	5.78E-01	CD69+ CD56lo (log)	9.08E-01
390	Streptobacillus	7.41E-01	(.2558127744;2.147319196)	5.81E-01	CD69+ (log)	9.10E-01
391	Pasteurellaceae_unclassified	1.54E+00	(.3291057743;7.213351953)	5.83E-01	NKT CD8+ total (log)	9.11E-01
392	Atopobium	6.94E-01	(.1862062724;2.58314051)	5.85E-01	CD56lo (log)	9.13E-01
393	Streptococcus	1.18E+00	(.6467280028;2.160917202)	5.87E-01	CD25+ CD56lo (log)	9.13E-01
394	Neisseria	1.21E+00	(.6129630807;2.369669677)	5.88E-01	CD25+ CD56lo (log)	9.13E-01
395	Comamonadaceae_unclassified	1.65E+00	(.2685667656;10.11601411)	5.89E-01	CD56lo (log)	9.13E-01
396	Micrococcaceae_unclassified	1.42E+00	(.3985004354;5.034885504)	5.91E-01	CD25+ CD56hi (log)	9.13E-01
397	Unclassified	1.32E+00	(.4784311942;3.649900814)	5.91E-01	CD69+ CD56lo (log)	9.13E-01
398	Micrococcaceae_unclassified	9.16E-01	(.663818104;1.264012232)	5.93E-01	CD56lo (sqrt)	9.13E-01
399	Actinomyces	6.26E-01	(.1104304327;3.544888306)	5.96E-01	CD56hi (sqrt)	9.13E-01
400	Oribacterium	6.35E-01	(.117105655;3.44562843)	5.99E-01	CD56hi (sqrt)	9.15E-01
401	Lachnospiraceae_unclassified	7.49E-01	(.2492854362;2.248452653)	6.06E-01	CD56lo (log)	9.23E-01
402	Campylobacter	1.18E+00	(.6259765788;2.23144576)	6.06E-01	NKT CD8+ total (log)	9.23E-01
403	Pasteurellaceae_unclassified	6.44E-01	(.1197631994;3.459528693)	6.08E-01	CD56lo (log)	9.23E-01
404	Lautropia	7.27E-01	(.2139176318;2.470628075)	6.09E-01	CD69+ CD56hi (log)	9.23E-01
405	Acinetobacter	1.63E+00	(.2507149701;10.57743744)	6.09E-01	NK total (log)	9.23E-01
406	Lachnospiraceae_unclassified	6.69E-01	(.1428563205;3.135686687)	6.10E-01	CD69+ CD56lo (log)	9.23E-01
407	Selenomonas	7.03E-01	(.1799965188;2.743023875)	6.12E-01	CD69+ CD56lo (log)	9.23E-01
408	Acinetobacter	1.54E+00	(.2925735863;8.073931278)	6.12E-01	CD56lo (log)	9.23E-01
409	Lachnospiraceae_unclassified	7.40E-01	(.2316964482;2.366607262)	6.12E-01	NK total (log)	9.23E-01
410	Acinetobacter	1.13E+00	(.7040725946;1.813254118)	6.13E-01	NK total (sqrt)	9.23E-01
411	Sphingomonas	7.80E-01	(.2984847992;2.040723946)	6.13E-01	NKT CD8+ total (log)	9.23E-01
412	Neisseriaceae_unclassified	9.22E-01	(.6722488255;1.26420382)	6.14E-01	CD56lo (sqrt)	9.23E-01
413	Lachnospiraceae_unclassified	7.92E-01	(.3194061253;1.961974994)	6.14E-01	CD69+ CD56hi (log)	9.23E-01

414	Micrococcaceae_unclassified	1.35E+00	(.4149427915;4.394697093)	6.18E-01	CD69+ CD56hi (log)	9.23E-01
415	Veillonellaceae_unclassified	1.64E+00	(.2344698356;11.43729562)	6.19E-01	CD56hi (sqrt)	9.23E-01
416	Campylobacter	7.61E-01	(.2541052785;2.2795341)	6.26E-01	CD56hi (sqrt)	9.23E-01
417	Comamonadaceae_unclassified	1.29E+00	(.4588458606;3.626260062)	6.29E-01	CD69+ CD56lo (log)	9.23E-01
418	Treponema	8.47E-01	(.4319738183;1.662597533)	6.30E-01	CD56lo (sqrt)	9.23E-01
419	Treponema	6.56E-01	(.1161478728;3.702666243)	6.33E-01	CD69+ (log)	9.23E-01
420	Lautropia	6.72E-01	(.1293774798;3.490574141)	6.36E-01	CD56hi (sqrt)	9.26E-01
421	Atopobium	7.04E-01	(.1640393794;3.024715751)	6.37E-01	NK total (log)	9.26E-01
422	Paucibacter	1.36E+00	(.3776062942;4.897071222)	6.38E-01	CD25+ (log)	9.26E-01
423	Capnocytophaga	1.31E+00	(.4258735845;4.017604825)	6.39E-01	CD25+ CD56hi (log)	9.26E-01
424	Haemophilus	7.37E-01	(.2056574477;2.640024995)	6.39E-01	NK total (log)	9.26E-01
425	Neisseria	7.81E-01	(.2778852996;2.194793117)	6.39E-01	NK total (log)	9.26E-01
426	Lachnoanaerobaculum	1.29E+00	(.4451894689;3.725544427)	6.41E-01	NK total (log)	9.26E-01
427	Prevotella	1.36E+00	(.3705764854;4.992622631)	6.43E-01	CD56hi (sqrt)	9.26E-01
428	Acinetobacter	1.11E+00	(.7182778139;1.706562431)	6.45E-01	CD56lo (sqrt)	9.26E-01
429	Haemophilus	7.57E-01	(.2320146491;2.473064088)	6.45E-01	CD56lo (log)	9.26E-01
430	Neisseria	9.41E-01	(.7259750426;1.21974595)	6.46E-01	NK total (sqrt)	9.26E-01
431	Fusobacterium	1.19E+00	(.5616626751;2.516978141)	6.51E-01	CD25+ (log)	9.26E-01
432	Methylobacterium-Methylorubrum	6.41E-01	(.092565619;4.441548432)	6.53E-01	CD69+ CD56hi (log)	9.26E-01
433	Lachnoanaerobaculum	1.63E+00	(.1934227861;13.69620807)	6.54E-01	CD56hi (sqrt)	9.26E-01
434	Haemophilus	1.30E+00	(.4060506247;4.183025424)	6.56E-01	CD25+ (log)	9.26E-01
435	Capnocytophaga	7.92E-01	(.2825104822;2.221221283)	6.58E-01	CD69+ CD56hi (log)	9.26E-01
436	Rothia	1.45E+00	(.2805632067;7.454383864)	6.59E-01	CD56hi (sqrt)	9.26E-01
437	Corynebacterium	7.14E-01	(.158542354;3.212198199)	6.60E-01	CD69+ (log)	9.26E-01
438	Actinomyces	1.09E+00	(.7415823304;1.59949176)	6.63E-01	CD56lo (sqrt)	9.26E-01
439	Solobacterium	1.36E+00	(.3398595288;5.410114843)	6.66E-01	CD25+ CD56hi (log)	9.27E-01
440	Prevotellaceae_ge	1.28E+00	(.4134674883;3.956441659)	6.69E-01	CD25+ CD56lo (log)	9.29E-01
441	Weeksellaceae_unclassified	1.05E+00	(.8291004486;1.334107381)	6.78E-01	NK total (sqrt)	9.39E-01
442	Haemophilus	1.28E+00	(.3979920395;4.086076981)	6.82E-01	CD25+ CD56lo (log)	9.43E-01
443	Lachnospiraceae_unclassified	7.12E-01	(.1393121024;3.641790443)	6.84E-01	CD69+ (log)	9.43E-01
444	Lachnoanaerobaculum	8.27E-01	(.3309698609;2.065069089)	6.84E-01	CD69+ (log)	9.43E-01
445	Streptobacillus	8.20E-01	(.3147411231;2.137429695)	6.85E-01	CD56lo (log)	9.43E-01
446	Alloprevotella	1.27E+00	(.3976176254;4.067553847)	6.85E-01	CD69+ CD56lo (log)	9.43E-01
447	Corynebacterium	7.30E-01	(.1594316974;3.345000633)	6.86E-01	CD69+ CD56lo (log)	9.43E-01
448	Veillonellaceae_unclassified	1.41E+00	(.2649130612;7.520070631)	6.86E-01	CD56hi (sqrt)	9.43E-01
449	Haemophilus	9.30E-01	(.6494331098;1.330665066)	6.90E-01	NK total (sqrt)	9.43E-01
450	Oribacterium	8.42E-01	(.360033568;1.968419824)	6.91E-01	CD25+ CD56lo (log)	9.43E-01
451	Streptobacillus	8.06E-01	(.2781879211;2.336337378)	6.91E-01	CD69+ CD56lo (log)	9.43E-01
452	Alysiella	1.26E+00	(.4035061176;3.920604313)	6.93E-01	NK total (log)	9.43E-01
453	Actinobacillus	8.43E-01	(.3599574063;1.972556171)	6.93E-01	CD25+ (log)	9.43E-01
454	Paucibacter	1.40E+00	(.2582119594;7.605505374)	6.96E-01	CD25+ CD56lo (log)	9.43E-01
455	Porphyromonas	7.86E-01	(.2352024282;2.629306619)	6.96E-01	CD25+ CD56hi (log)	9.43E-01
456	Lachnoanaerobaculum	1.06E+00	(.7877402503;1.42891938)	6.97E-01	CD56lo (sqrt)	9.43E-01
457	Selenomonas	1.22E+00	(.4522448676;3.267892697)	6.99E-01	NKT CD8+ total (log)	9.43E-01
458	Campylobacter	7.95E-01	(.2473329836;2.557537797)	7.01E-01	NK total (log)	9.43E-01
459	Pasteurellaceae_unclassified	1.22E+00	(.4367593469;3.424431365)	7.02E-01	CD25+ (log)	9.43E-01
460	Rothia	8.56E-01	(.3840931822;1.909783861)	7.05E-01	CD25+ CD56lo (log)	9.43E-01

461	Tannerella	9.34E-01	(.6557359966;1.330701407)	7.06E-01	NK total (sqrt)	9.43E-01
462	Catonella	1.29E+00	(.3392843676;4.925027341)	7.07E-01	CD69+ CD56lo (log)	9.43E-01
463	Selenomonas	8.48E-01	(.3557479826;2.01924238)	7.09E-01	CD56lo (log)	9.43E-01
464	Alysiella	1.32E+00	(.3078714631;5.642464908)	7.10E-01	CD25+ CD56lo (log)	9.43E-01
465	Actinomyces	8.13E-01	(.2715835018;2.435980346)	7.12E-01	CD69+ CD56lo (log)	9.43E-01
466	Prevotellaceae_unclassified	7.93E-01	(.2303858578;2.730333473)	7.13E-01	CD25+ CD56hi (log)	9.43E-01
467	Treponema	8.65E-01	(.3989840017;1.876183458)	7.14E-01	CD25+ (log)	9.43E-01
468	Veillonellaceae_unclassified	7.81E-01	(.207432404;2.938960623)	7.14E-01	CD69+ CD56hi (log)	9.43E-01
469	Prevotella	1.14E+00	(.5740783656;2.244686026)	7.15E-01	NKT CD8+ total (log)	9.43E-01
470	Atopobium	8.40E-01	(.3263668726;2.163194371)	7.18E-01	CD25+ CD56lo (log)	9.43E-01
471	Streptococcus	1.16E+00	(.5060447828;2.678261957)	7.21E-01	CD69+ (log)	9.43E-01
472	Tannerella	9.44E-01	(.6838130697;1.303449597)	7.27E-01	CD56lo (sqrt)	9.43E-01
473	Catonella	1.23E+00	(.3724089256;4.079183993)	7.32E-01	CD56hi (sqrt)	9.46E-01
474	Rothia	1.32E+00	(.2644064712;6.633517056)	7.33E-01	CD25+ CD56hi (log)	9.46E-01
475	Micrococcaceae_unclassified	8.63E-01	(.3679230543;2.023546187)	7.34E-01	CD25+ CD56lo (log)	9.46E-01
476	Treponema	1.70E+00	(.0775559758;37.20343016)	7.37E-01	CD25+ CD56hi (log)	9.46E-01
477	Catonella	1.27E+00	(.3037929097;5.317193968)	7.43E-01	CD69+ (log)	9.51E-01
478	Streptococcus	8.60E-01	(.3500668877;2.113784805)	7.43E-01	CD56hi (sqrt)	9.51E-01
479	Campylobacter	8.48E-01	(.3159080234;2.278517945)	7.44E-01	CD56hi (sqrt)	9.51E-01
480	Lachnoanaerobaculum	1.18E+00	(.4367676852;3.18329533)	7.45E-01	CD56lo (log)	9.51E-01
481	Prevotella	1.26E+00	(.3034860317;5.248110061)	7.49E-01	CD56hi (sqrt)	9.51E-01
482	Veillonellaceae_unclassified	1.15E+00	(.4883935809;2.707094313)	7.49E-01	NKT CD8+ total (log)	9.51E-01
483	Actinomyces	1.26E+00	(.3055017532;5.191766246)	7.50E-01	CD56lo (log)	9.51E-01
484	Lachnoanaerobaculum	1.05E+00	(.7704748774;1.435941127)	7.50E-01	NK total (sqrt)	9.51E-01
485	Campylobacter	8.50E-01	(.2962607912;2.440003715)	7.63E-01	CD56lo (log)	9.61E-01
486	Haemophilus	1.17E+00	(.4240725725;3.218234821)	7.64E-01	CD25+ CD56hi (log)	9.61E-01
487	Fusobacterium	8.58E-01	(.3144091649;2.343827478)	7.66E-01	CD69+ (log)	9.61E-01
488	Lachnoanaerobaculum	1.16E+00	(.4234360268;3.17456656)	7.73E-01	CD69+ CD56hi (log)	9.68E-01
489	Atopobium	9.42E-01	(.6288928169;1.412315573)	7.74E-01	NK total (sqrt)	9.68E-01
490	Catonella	8.98E-01	(.4288471305;1.881431188)	7.76E-01	CD25+ CD56lo (log)	9.68E-01
491	Sphingomonas	1.33E+00	(.1875304156;9.398012328)	7.77E-01	CD56hi (sqrt)	9.68E-01
492	Porphyromonas	1.18E+00	(.3632174906;3.849724787)	7.81E-01	CD56hi (sqrt)	9.70E-01
493	Cardiobacterium	6.73E-01	(.0411808563;10.9858832)	7.81E-01	CD25+ CD56hi (log)	9.70E-01
494	Comamonadaceae_unclassified	1.18E+00	(.3701929958;3.751104658)	7.81E-01	CD69+ (log)	9.70E-01
495	Pasteurellaceae_unclassified	1.39E+00	(.1342135459;14.44287312)	7.82E-01	CD25+ CD56hi (log)	9.70E-01
496	Bradyrhizobium	1.15E+00	(.4278394145;3.083562256)	7.83E-01	CD25+ (log)	9.70E-01
497	Selenomonas	8.77E-01	(.3427937141;2.242161606)	7.84E-01	NK total (log)	9.70E-01
498	Sphingomonas	1.32E+00	(.1851861063;9.340710582)	7.84E-01	CD25+ CD56lo (log)	9.70E-01
499	Fusobacterium	8.42E-01	(.2432275086;2.913008767)	7.86E-01	CD56hi (sqrt)	9.70E-01
500	Campylobacter	8.42E-01	(.23810541;2.97404937)	7.89E-01	CD25+ CD56lo (log)	9.70E-01
501	Alysiella	7.73E-01	(.1175927304;5.084633382)	7.89E-01	CD69+ (log)	9.70E-01
502	Weeksellaceae_unclassified	8.88E-01	(.3717378937;2.121510554)	7.89E-01	CD25+ CD56lo (log)	9.70E-01
503	Cardiobacterium	7.94E-01	(.1459211971;4.323015976)	7.90E-01	CD56hi (sqrt)	9.70E-01
504	Comamonadaceae_unclassified	7.70E-01	(.1087266459;5.450126034)	7.93E-01	CD69+ CD56hi (log)	9.70E-01
505	Acinetobacter	1.34E+00	(.1423745228;12.69631494)	7.96E-01	CD25+ CD56hi (log)	9.70E-01
506	Unclassified	1.15E+00	(.3959696032;3.340209821)	7.97E-01	CD69+ (log)	9.70E-01
507	Porphyromonas	8.93E-01	(.3749119918;2.12475978)	7.97E-01	CD69+ CD56hi (log)	9.70E-01
508	Cardiobacterium	8.79E-01	(.3189088353;2.424081678)	8.04E-01	CD25+ (log)	9.70E-01

509	Streptobacillus	8.79E-01	(.3167859629;2.439908422)	8.05E-01	NK total (log)	9.70E-01
510	Micrococcaceae_unclassified	9.24E-01	(.4886228507;1.748373851)	8.09E-01	CD25+ (log)	9.70E-01
511	Alysiella	9.62E-01	(.699216666;1.323437267)	8.12E-01	CD56lo (sqrt)	9.71E-01
512	Veillonella	8.60E-01	(.2475771194;2.987149194)	8.12E-01	CD69+ (log)	9.71E-01
513	Flavobacterium	1.12E+00	(.4402630617;2.847719244)	8.12E-01	CD69+ CD56lo (log)	9.71E-01
514	Lachnospiraceae_unclassified	9.64E-01	(.7084026666;1.31158539)	8.15E-01	NK total (sqrt)	9.71E-01
515	Weeksellaceae_unclassified	1.11E+00	(.4650364593;2.633558915)	8.19E-01	CD69+ CD56hi (log)	9.71E-01
516	Comamonadaceae_unclassified	8.86E-01	(.3086862985;2.540637856)	8.21E-01	NKT CD8+ total (log)	9.72E-01
517	Campylobacter	9.66E-01	(.7099689659;1.313115735)	8.23E-01	NK total (sqrt)	9.73E-01
518	Alysiella	8.19E-01	(.141963115;4.723793918)	8.23E-01	CD69+ CD56lo (log)	9.73E-01
519	Porphyromonas	8.59E-01	(.2253160888;3.273847741)	8.24E-01	CD56hi (sqrt)	9.73E-01
520	Unclassified	9.11E-01	(.4011925177;2.070299476)	8.25E-01	CD69+ CD56hi (log)	9.73E-01
521	Streptobacillus	9.72E-01	(.7539243699;1.252662404)	8.25E-01	CD56lo (sqrt)	9.73E-01
522	Flavobacterium	8.45E-01	(.186236559;3.834305599)	8.27E-01	CD56lo (log)	9.73E-01
523	Atopobium	9.60E-01	(.6521357111;1.413931508)	8.37E-01	CD56lo (sqrt)	9.78E-01
524	Cardiobacterium	8.29E-01	(.1279720403;5.368545322)	8.44E-01	CD56hi (sqrt)	9.84E-01
525	Bradyrhizobium	9.16E-01	(.3777053905;2.222919176)	8.47E-01	NKT CD8+ total (log)	9.85E-01
526	Methylobacterium-Methylorubrum	1.06E+00	(.6008153053;1.860206771)	8.47E-01	NK total (sqrt)	9.85E-01
527	Flavobacterium	1.12E+00	(.3318055565;3.772075735)	8.56E-01	CD25+ CD56lo (log)	9.93E-01
528	Capnocytophaga	1.12E+00	(.338621162;3.680273369)	8.57E-01	CD25+ CD56lo (log)	9.93E-01
529	Campylobacter	9.74E-01	(.7281251976;1.303087493)	8.60E-01	CD56lo (sqrt)	9.93E-01
530	Sphingomonas	8.88E-01	(.2360797613;3.337622013)	8.60E-01	CD69+ (log)	9.93E-01
531	Rothia	9.02E-01	(.2786886521;2.919887852)	8.63E-01	CD69+ (log)	9.94E-01
532	Streptococcus	9.81E-01	(.7876460995;1.22215249)	8.65E-01	NK total (sqrt)	9.94E-01
533	Planococcaceae_unclassified	7.91E-01	(.0519293915;12.05167472)	8.66E-01	CD56hi (sqrt)	9.94E-01
534	Flavobacterium	1.09E+00	(.3927669222;3.030662241)	8.67E-01	CD69+ (log)	9.94E-01
535	Streptococcus	1.07E+00	(.4823285965;2.373326824)	8.68E-01	CD69+ CD56lo (log)	9.94E-01
536	Bradyrhizobium	1.11E+00	(.3168583069;3.888897423)	8.70E-01	CD69+ (log)	9.94E-01
537	Veillonella	9.20E-01	(.3383050604;2.503502467)	8.71E-01	CD25+ CD56hi (log)	9.94E-01
538	Oxalobacteraceae_unclassified	1.04E+00	(.6198429342;1.757349834)	8.72E-01	CD56lo (sqrt)	9.94E-01
539	Streptococcus	9.83E-01	(.7922810158;1.219377726)	8.75E-01	CD56lo (sqrt)	9.94E-01
540	Alysiella	1.09E+00	(.3769051008;3.142149149)	8.76E-01	CD56lo (log)	9.94E-01
541	Fusobacterium	9.18E-01	(.2961324227;2.846486916)	8.82E-01	CD25+ CD56lo (log)	9.97E-01
542	Alloprevotella	1.09E+00	(.345081858;3.443617776)	8.83E-01	CD69+ (log)	9.97E-01
543	Lachnospiraceae_unclassified	9.78E-01	(.7253704031;1.318401975)	8.84E-01	CD56lo (sqrt)	9.97E-01
544	Flavobacterium	8.87E-01	(.1730511833;4.542625824)	8.85E-01	NK total (log)	9.97E-01
545	Methylobacterium-Methylorubrum	1.04E+00	(.6102025597;1.771733052)	8.86E-01	CD56lo (sqrt)	9.97E-01
546	Oxalobacteraceae_unclassified	8.91E-01	(.1822762228;4.35985644)	8.87E-01	CD69+ CD56hi (log)	9.97E-01
547	Prevotellaceae_ge	1.05E+00	(.563582943;1.939977277)	8.87E-01	CD25+ (log)	9.97E-01
548	Neisseria	9.20E-01	(.285968461;2.962584109)	8.89E-01	CD69+ (log)	9.97E-01
549	Atopobium	1.05E+00	(.4644469619;2.388910085)	9.01E-01	NKT CD8+ total (log)	1.00E+00
550	Alysiella	9.30E-01	(.2735846942;3.159609491)	9.07E-01	CD56hi (sqrt)	1.00E+00
551	Methylobacterium-Methylorubrum	8.97E-01	(.143722463;5.600790144)	9.08E-01	CD56lo (log)	1.00E+00
552	Unclassified	1.05E+00	(.4466176356;2.474804758)	9.09E-01	CD25+ CD56lo (log)	1.00E+00
553	Micrococcaceae_unclassified	1.05E+00	(.4139067226;2.686451666)	9.11E-01	NK total (log)	1.00E+00
554	Fusobacterium	1.07E+00	(.3368605507;3.37679671)	9.13E-01	CD56hi (sqrt)	1.00E+00
555	Veillonella	9.36E-01	(.2729654213;3.208745081)	9.16E-01	CD69+ CD56lo (log)	1.00E+00

556	Streptobacillus	9.86E-01	(.7584187237;1.282446226)	9.18E-01	NK total (sqrt)	1.00E+00
557	Methylobacterium-Methylorubrum	9.41E-01	(.2829869502;3.127800021)	9.21E-01	CD69+ (log)	1.00E+00
558	Methylobacterium-Methylorubrum	9.37E-01	(.2559070045;3.429089423)	9.21E-01	NKT CD8+ total (log)	1.00E+00
559	Campylobacter	9.61E-01	(.4271016671;2.162460924)	9.23E-01	CD25+ (log)	1.00E+00
560	Capnocytophaga	9.45E-01	(.288690389;3.094483906)	9.26E-01	CD56lo (log)	1.00E+00
561	Lachnospiraceae_unclassified	1.04E+00	(.4281898121;2.533606624)	9.28E-01	CD25+ CD56lo (log)	1.00E+00
562	Methylobacterium-Methylorubrum	9.51E-01	(.302166046;2.993394417)	9.32E-01	CD69+ CD56lo (log)	1.00E+00
563	Actinomyces	1.05E+00	(.3676806793;2.975594051)	9.33E-01	NKT CD8+ total (log)	1.00E+00
564	Rothia	9.54E-01	(.3173564;2.866745754)	9.33E-01	CD69+ CD56lo (log)	1.00E+00
565	Oxalobacteraceae_unclassified	9.22E-01	(.1360328574;6.248287247)	9.34E-01	NK total (log)	1.00E+00
566	Actinomyces	1.01E+00	(.7041840834;1.457332129)	9.44E-01	NK total (sqrt)	1.00E+00
567	Planococcaceae_unclassified	9.28E-01	(.1130307157;7.623534431)	9.45E-01	CD56hi (sqrt)	1.00E+00
568	Oxalobacteraceae_unclassified	1.02E+00	(.5867049834;1.772140257)	9.45E-01	NK total (sqrt)	1.00E+00
569	Lautropia	9.46E-01	(.1752336482;5.103081759)	9.48E-01	CD69+ CD56lo (log)	1.00E+00
570	Paucibacter	9.46E-01	(.1763723186;5.073678841)	9.48E-01	CD56hi (sqrt)	1.00E+00
571	Capnocytophaga	9.89E-01	(.7183696391;1.362938066)	9.48E-01	CD56lo (sqrt)	1.00E+00
572	Oxalobacteraceae_unclassified	9.62E-01	(.2784957386;3.319660753)	9.51E-01	CD69+ (log)	1.00E+00
573	Flavobacterium	9.85E-01	(.6122506479;1.585507587)	9.51E-01	NK total (sqrt)	1.00E+00
574	Bradyrhizobium	9.47E-01	(.1627201265;5.512887045)	9.52E-01	CD25+ CD56hi (log)	1.00E+00
575	Oxalobacteraceae_unclassified	9.48E-01	(.1667331861;5.391043562)	9.52E-01	CD56lo (log)	1.00E+00
576	Streptococcus	1.03E+00	(.3769314491;2.805464199)	9.56E-01	CD56hi (sqrt)	1.00E+00
577	Corynebacterium	9.77E-01	(.4171408959;2.286865902)	9.57E-01	CD25+ (log)	1.00E+00
578	Sphingomonas	9.55E-01	(.1592944219;5.72541531)	9.60E-01	CD25+ CD56hi (log)	1.00E+00
579	Alysiella	1.01E+00	(.726572316;1.399080865)	9.61E-01	NK total (sqrt)	1.00E+00
580	Micrococcaceae_unclassified	9.77E-01	(.3843833643;2.483572743)	9.61E-01	CD56lo (log)	1.00E+00
581	Actinobacillus	1.03E+00	(.3376178868;3.126901526)	9.62E-01	CD69+ (log)	1.00E+00
582	Streptococcus	1.02E+00	(.4733812766;2.191648427)	9.62E-01	CD56lo (log)	1.00E+00
583	Streptococcus	1.02E+00	(.4453380523;2.336164796)	9.63E-01	NK total (log)	1.00E+00
584	Pasteurellaceae_unclassified	9.34E-01	(.0534703645;16.33007681)	9.63E-01	CD69+ (log)	1.00E+00
585	Capnocytophaga	1.01E+00	(.7272146529;1.395578429)	9.65E-01	NK total (sqrt)	1.00E+00
586	Weeksellaceae_unclassified	1.02E+00	(.3196346855;3.286125147)	9.67E-01	CD56hi (sqrt)	1.00E+00
587	Actinobacillus	1.02E+00	(.3435470846;3.040137676)	9.69E-01	CD69+ CD56lo (log)	1.00E+00
588	Fusobacterium	9.81E-01	(.366606942;2.624532955)	9.69E-01	CD69+ CD56lo (log)	1.00E+00
589	Lautropia	9.66E-01	(.1653039203;5.648423553)	9.70E-01	CD69+ (log)	1.00E+00
590	Streptococcus	1.01E+00	(.6458433512;1.575344439)	9.70E-01	CD25+ (log)	1.00E+00
591	Neisseria	9.79E-01	(.298111566;3.216726755)	9.72E-01	CD69+ CD56lo (log)	1.00E+00
592	Sphingomonas	1.02E+00	(.2872098226;3.618849822)	9.76E-01	CD69+ CD56lo (log)	1.00E+00
593	Planococcaceae_unclassified	1.02E+00	(.3575568064;2.886316212)	9.76E-01	CD25+ CD56lo (log)	1.00E+00
594	Alysiella	9.79E-01	(.2229268658;4.297548887)	9.77E-01	CD56hi (sqrt)	1.00E+00
595	Enterococcaceae_unclassified	1.02E+00	(.2819493917;3.672740434)	9.79E-01	CD25+ CD56lo (log)	1.00E+00
596	Flavobacterium	1.01E+00	(.6368859272;1.587626811)	9.81E-01	CD56lo (sqrt)	1.00E+00
597	Acinetobacter	9.75E-01	(.1027594227;9.250307336)	9.82E-01	CD56hi (sqrt)	1.00E+00
598	Cardiobacterium	1.01E+00	(.3298943796;3.107090534)	9.83E-01	NKT CD8+ total (log)	1.00E+00
599	Oxalobacteraceae_unclassified	1.01E+00	(.3116524036;3.291154087)	9.83E-01	CD69+ CD56lo (log)	1.00E+00

600	Selenomonas	1.01E+00	(.2693286187;3.797864434)	9.87E-01	CD56hi (sqrt)	1.00E+00
601	Flavobacterium	9.89E-01	(.252329212;3.875668625)	9.87E-01	CD69+ CD56hi (log)	1.00E+00
602	Treponema	9.85E-01	(.1484931879;6.540088014)	9.88E-01	CD69+ CD56lo (log)	1.00E+00
603	Veillonellaceae_unclassified	9.92E-01	(.2457998196;4.003109874)	9.91E-01	CD25+ CD56hi (log)	1.00E+00
604	Lachnospiraceae_unclassified	9.95E-01	(.3793466628;2.60763006)	9.91E-01	NKT CD8+ total (log)	1.00E+00
605	Weeksellaceae_unclassified	1.01E+00	(.3545352336;2.849091219)	9.92E-01	CD56hi (sqrt)	1.00E+00
606	Capnocytophaga	1.01E+00	(.2893184124;3.495305493)	9.93E-01	NK total (log)	1.00E+00
607	Methylobacterium-Methylorubrum	1.01E+00	(.1308010046;7.743368211)	9.95E-01	NK total (log)	1.00E+00
608	Pasteurellaceae_unclassified	9.94E-01	(.0673145157;14.67576987)	9.96E-01	CD69+ CD56lo (log)	1.00E+00
609	Treponema	1.00E+00	(.3470373689;2.895004772)	9.97E-01	NKT CD8+ total (log)	1.00E+00
610	Terrisporobacter	1.00E+00	(.4093232837;2.447285816)	9.98E-01	CD25+ (log)	1.00E+00
611	Actinomyces	1.00E+00	(.2392429997;4.185046787)	9.99E-01	NK total (log)	1.00E+00

OR=odds ratio; 95%-CI=95% confidence interval

p-values from fractional regressions adjusting for group variable, age, bmi, and sex

Table S4F: Associations of NK cell counts with genera found in the salivary microbiome

	Genus	OR	95%-CI	p-value	NK cell markers	FDR
1	**Unclassified**	**2.09E+00**	**(1.513990684;2.889056763)**	**7.61E-06**	**CD25+ CD56lo (log)**	**4.85E-03**
2	**Pseudomonas**	**9.79E+00**	**(3.238021924;29.5999191)**	**5.31E-05**	**CD25+ (log)**	**1.69E-02**
3	**Alloprevotella**	**9.37E+01**	**(10.01364552;877.2148598)**	**6.92E-05**	**CD25+ CD56hi (log)**	**1.69E-02**
4	Alloprevotella	4.55E-01	(.295103607;.7019074501)	3.69E-04	NK total (sqrt)	5.88E-02
5	Comamonadaceae_ unclassified	3.85E+00	(1.82762027;8.112348007)	3.91E-04	CD69+ CD56lo (log)	5.88E-02
6	Oxalobacteraceae_ unclassified	6.10E+00	(2.212058691;16.82724734)	4.76E-04	CD56hi (sqrt)	5.88E-02
7	Fluviicola	3.96E-02	(.006283124;.2500804293)	5.93E-04	CD25+ CD56hi (log)	5.88E-02
8	Methylobacterium-Methylorubrum	7.21E-01	(.5984271327;.8695201016)	6.11E-04	NK total (sqrt)	5.88E-02
9	Streptobacillus	2.40E-02	(.0027163268;.2116362755)	7.86E-04	CD25+ CD56hi (log)	5.88E-02
10	Veillonella	2.09E-01	(.0834132571;.524583484)	8.52E-04	CD69+ (log)	5.88E-02
11	Comamonadaceae_ unclassified	3.69E+00	(1.680120975;8.090177929)	1.14E-03	CD69+ (log)	6.59E-02
12	Actinomyces	4.07E-01	(.235123826;.7052838706)	1.35E-03	CD25+ CD56lo (log)	7.15E-02
13	Pseudomonas	1.88E-01	(.0654750094;.5392253372)	1.88E-03	CD56lo (log)	9.22E-02
14	Alloprevotella	8.93E-02	(.019349499;.412328931)	1.97E-03	NK total (log)	9.22E-02
15	Alloprevotella	5.02E-01	(.3243899226;.778178837)	2.05E-03	CD56lo (sqrt)	9.22E-02
16	Veillonella	2.38E-01	(.0953187786;.5931238107)	2.07E-03	CD69+ CD56lo (log)	9.22E-02
17	Methylobacterium-Methylorubrum	7.55E-01	(.6316572824;.9034153556)	2.12E-03	CD56lo (sqrt)	9.22E-02
18	Pseudomonas	1.78E-01	(.0571882877;.5524161988)	2.83E-03	NK total (log)	1.00E-01
19	Micrococcaceae_un classified	1.07E-01	(.0234014459;.4851579638)	3.79E-03	CD56hi (sqrt)	1.27E-01
20	Prevotellaceae_uncl assified	2.16E-01	(.0758438124;.6169216623)	4.19E-03	CD25+ (log)	1.34E-01
21	Fusobacterium	9.10E-02	(.0168817463;.4907270263)	5.30E-03	CD25+ CD56hi (log)	1.61E-01
22	Caulobacter	2.43E-01	(.0873091902;.6745969144)	6.63E-03	CD25+ (log)	1.92E-01
23	Comamonas	3.13E+00	(1.369909856;7.133520858)	6.78E-03	CD69+ CD56lo (log)	1.92E-01
24	Micrococcaceae_un classified	6.52E-02	(.0086372628;.4920469417)	8.11E-03	CD56hi (sqrt)	2.15E-01
25	Oxalobacteraceae_ unclassified	4.71E+00	(1.49318256;14.86837537)	8.20E-03	CD56hi (sqrt)	2.15E-01
26	Alloprevotella	1.27E-01	(.0268224991;.602190579)	9.35E-03	CD56lo (log)	2.29E-01
27	Atopobium	4.90E+00	(1.469330241;16.31182976)	9.69E-03	NKT CD8+ total (log)	2.29E-01
28	Pseudomonas	6.41E-01	(.4539125303;.9061422077)	1.18E-02	NK total (sqrt)	2.68E-01
29	Porphyromonas	1.97E-01	(.0554022683;.699143899)	1.20E-02	CD25+ CD56hi (log)	2.68E-01
30	Pseudomonas	6.68E-01	(.4868317676;.9164387145)	1.24E-02	CD56lo (sqrt)	2.68E-01
31	Acinetobacter	3.96E+00	(1.330652855;11.7923763)	1.34E-02	CD69+ CD56lo (log)	2.75E-01
32	Xanthobacteraceae _unclassified	1.30E-01	(.0256971212;.6582408383)	1.37E-02	CD25+ CD56lo (log)	2.75E-01
33	Acinetobacter	3.80E+00	(1.312926326;11.00299097)	1.38E-02	CD69+ (log)	2.75E-01
34	Comamonas	2.91E+00	(1.215724777;6.959660585)	1.64E-02	CD69+ (log)	3.08E-01
35	Comamonadaceae_ unclassified	2.98E+00	(1.202886581;7.360350739)	1.83E-02	CD56hi (sqrt)	3.33E-01
36	Actinomyces	1.89E+00	(1.109252682;3.204012106)	1.91E-02	NKT CD8+ total (log)	3.38E-01
37	Capnocytophaga	1.01E-01	(.0149080323;.6888132279)	1.92E-02	CD25+ CD56hi (log)	3.38E-01
38	Methylobacterium-Methylorubrum	4.08E-01	(.1917947674;.866415654)	1.97E-02	NK total (log)	3.38E-01
39	Capnocytophaga	3.59E-01	(.1515151886;.8505488969)	1.99E-02	NKT CD8+ total (log)	3.38E-01
40	Aquabacterium	2.81E+00	(1.165006234;6.792631667)	2.15E-02	CD69+ CD56lo (log)	3.42E-01
41	Pseudomonas	3.52E+00	(1.203413565;10.29458367)	2.16E-02	CD25+ CD56lo (log)	3.42E-01
42	Aquabacterium	2.84E+00	(1.16533125;6.941856155)	2.17E-02	CD69+ (log)	3.42E-01
43	Unclassified	1.96E+00	(1.079612385;3.57638517)	2.71E-02	CD69+ CD56hi (log)	4.01E-01

44	Comamonadaceae_unclassified	3.22E+00	(1.122943842;9.233659966)	2.96E-02	CD56hi (sqrt)	4.28E-01
45	Capnocytophaga	3.21E-01	(.113565079;.9095704484)	3.25E-02	CD69+ (log)	4.60E-01
46	Methylobacterium-Methylorubrum	4.59E-01	(.2224521502;.9459573351)	3.48E-02	CD56lo (log)	4.82E-01
47	Absconditabacteriales_(SR1)_ge	2.71E+00	(1.059932618;6.920168227)	3.74E-02	CD25+ CD56lo (log)	5.07E-01
48	Comamonas	2.72E+00	(1.056431141;7.027233669)	3.81E-02	CD56hi (sqrt)	5.07E-01
49	Cardiobacterium	3.01E-01	(.0964545262;.941378238)	3.90E-02	CD25+ CD56lo (log)	5.07E-01
50	Streptobacillus	3.09E-01	(.101507994;.9430348526)	3.91E-02	CD69+ CD56lo (log)	5.07E-01
51	Alloprevotella	2.91E-01	(.0896283732;.9422792865)	3.95E-02	NKT CD8+ total (log)	5.07E-01
52	Streptobacillus	2.99E-01	(.0933837082;.9548101936)	4.16E-02	CD69+ (log)	5.09E-01
53	Selenomonas	5.83E-02	(.0035706914;.9534256379)	4.62E-02	CD25+ CD56hi (log)	5.55E-01
54	Capnocytophaga	3.50E-01	(.1245100775;.9858822358)	4.69E-02	CD69+ CD56lo (log)	5.55E-01
55	Parvimonas	3.58E+00	(1.00854608;12.67248087)	4.85E-02	CD25+ (log)	5.61E-01
56	Campylobacter	1.10E-01	(.0120428202;1.010181004)	5.11E-02	CD25+ CD56hi (log)	5.81E-01
57	Catonella	1.92E+00	(.988103601;3.745165136)	5.43E-02	NKT CD8+ total (log)	6.06E-01
58	Micrococcaceae_unclassified	8.75E+00	(.9302777443;82.29611178)	5.79E-02	CD25+ CD56hi (log)	6.35E-01
59	Sphingomonas	3.22E-01	(.0998352846;1.039238345)	5.80E-02	CD25+ CD56hi (log)	6.35E-01
60	Acinetobacter	4.16E+00	(.9420879971;18.39369344)	5.99E-02	CD25+ CD56lo (log)	6.36E-01
61	Enterococcaceae_unclassified	3.45E+00	(.9483070928;12.54694053)	6.02E-02	CD69+ CD56hi (log)	6.36E-01
62	Bradyrhizobium	2.19E+00	(.9655370947;4.952891334)	6.07E-02	CD69+ (log)	6.36E-01
63	Methylobacterium-Methylorubrum	3.78E-01	(.1362791751;1.048087769)	6.15E-02	CD56hi (sqrt)	6.36E-01
64	Prevotella	1.79E-01	(.0294093133;1.090836077)	6.21E-02	CD56hi (sqrt)	6.36E-01
65	Planococcaceae_unclassified	1.86E+00	(.9688515769;3.576972766)	6.22E-02	CD25+ CD56lo (log)	6.36E-01
66	Comamonadaceae_unclassified	3.93E-01	(.1472515263;1.051384747)	6.29E-02	NK total (log)	6.36E-01
67	Flavobacterium	4.70E+00	(.9201395916;24.01777781)	6.29E-02	CD56hi (sqrt)	6.36E-01
68	Planococcaceae_unclassified	1.86E+00	(.9670775239;3.583308549)	6.29E-02	NKT CD8+ total (log)	6.36E-01
69	Streptobacillus	3.63E-01	(.1242188952;1.059006557)	6.36E-02	CD25+ CD56lo (log)	6.36E-01
70	Enterococcaceae_unclassified	7.12E-01	(.4973490038;1.020407724)	6.43E-02	CD56lo (sqrt)	6.36E-01
71	Paucibacter	2.25E+00	(.9466687677;5.32433227)	6.64E-02	CD69+ (log)	6.36E-01
72	Campylobacter	3.10E-01	(.0883642236;1.086885251)	6.73E-02	CD69+ (log)	6.36E-01
73	Oxalobacteraceae_unclassified	1.64E-01	(.0234989897;1.140636321)	6.77E-02	CD25+ CD56hi (log)	6.36E-01
74	Flavobacterium	4.96E+00	(.8815202959;27.86655347)	6.92E-02	CD56hi (sqrt)	6.36E-01
75	Prevotella	3.97E-01	(.1456643152;1.08134182)	7.08E-02	NKT CD8+ total (log)	6.36E-01
76	Prevotella	2.47E-01	(.0532757537;1.146180447)	7.42E-02	CD56hi (sqrt)	6.36E-01
77	Aquabacterium	2.47E+00	(.9155431511;6.658119471)	7.42E-02	CD56hi (sqrt)	6.36E-01
78	Catonella	3.76E-01	(.1284655507;1.100455631)	7.42E-02	NK total (log)	6.36E-01
79	Prevotellaceae_ge	1.53E-01	(.019377566;1.205761238)	7.47E-02	CD69+ CD56lo (log)	6.36E-01
80	Paucibacter	2.57E+00	(.9082045252;7.253588067)	7.54E-02	CD25+ CD56hi (log)	6.36E-01
81	Acinetobacter	2.39E+00	(.9099201954;6.259348087)	7.70E-02	CD25+ (log)	6.36E-01
82	Alloprevotella	1.42E-01	(.0161227219;1.243723059)	7.79E-02	CD56hi (sqrt)	6.36E-01
83	Acinetobacter	3.74E+00	(.8616066977;16.19225585)	7.83E-02	CD56hi (sqrt)	6.36E-01
84	Comamonas	2.59E+00	(.8863888727;7.562490518)	8.20E-02	CD56hi (sqrt)	6.36E-01
85	Comamonadaceae_unclassified	4.28E-01	(.1619247777;1.128895524)	8.63E-02	CD56lo (log)	6.47E-01
86	Parvimonas	2.86E+00	(.8521193943;9.623581107)	8.89E-02	CD25+ CD56lo (log)	6.59E-01
87	Unclassified	1.41E+00	(.9453716807;2.091427569)	9.24E-02	CD25+ (log)	6.77E-01
88	Prevotellaceae_ge	1.68E-01	(.0211137939;1.343645598)	9.27E-02	CD69+ (log)	6.77E-01
89	Lachnospiraceae_unclassified	3.78E-01	(.121223547;1.179777253)	9.39E-02	NKT CD8+ total (log)	6.77E-01

90	Catonella	4.03E-01	(.139394029;1.167221059)	9.40E-02	CD56lo (log)	6.77E-01
91	Comamonadaceae_ unclassified	7.96E-01	(.6102048302;1.039639939)	9.41E-02	NK total (sqrt)	6.77E-01
92	Prevotellaceae_ge	3.97E-01	(.1343371685;1.172328352)	9.45E-02	NKT CD8+ total (log)	6.77E-01
93	Selenomonas	4.64E-01	(.1885655229;1.141562951)	9.46E-02	CD25+ (log)	6.77E-01
94	Veillonella	4.59E-01	(.1841979968;1.145033641)	9.50E-02	NKT CD8+ total (log)	6.77E-01
95	Actinobacillus	1.56E-01	(.017471047;1.396067855)	9.66E-02	CD56hi (sqrt)	6.77E-01
96	Campylobacter	3.52E-01	(.1027157953;1.20843323)	9.71E-02	CD69+ CD56lo (log)	6.77E-01
97	Enterococcaceae_u nclassified	7.17E-01	(.4839952211;1.063263987)	9.80E-02	NK total (sqrt)	6.77E-01
98	Acinetobacter	4.20E+00	(.7643628635;23.05460582)	9.88E-02	CD56hi (sqrt)	6.77E-01
99	Planococcaceae_un classified	1.75E+00	(.8999380612;3.391124893)	9.93E-02	CD69+ CD56lo (log)	6.77E-01
100	Weeksellaceae_uncl assified	6.54E-01	(.3930694259;1.089117002)	1.03E-01	CD25+ (log)	6.77E-01
101	Veillonella	2.30E-01	(.0385933078;1.367275627)	1.06E-01	CD25+ CD56hi (log)	6.77E-01
102	Planococcaceae_un classified	1.68E+00	(.8878053444;3.186457701)	1.11E-01	CD25+ (log)	6.92E-01
103	Enterococcaceae_u nclassified	3.39E-01	(.0898051163;1.28161409)	1.11E-01	CD56lo (log)	6.92E-01
104	Prevotellaceae_uncl assified	2.66E-01	(.0521126381;1.35637658)	1.11E-01	CD69+ (log)	6.92E-01
105	Enterococcaceae_u nclassified	2.26E+00	(.8281216117;6.183253906)	1.11E-01	CD25+ CD56lo (log)	6.92E-01
106	Cardiobacterium	3.76E-01	(.1115829017;1.265236223)	1.14E-01	CD69+ (log)	6.92E-01
107	Methylobacterium- Methylorubrum	3.62E-01	(.1029051704;1.276841825)	1.14E-01	CD56hi (sqrt)	6.92E-01
108	Cardiobacterium	4.00E-01	(.1256605006;1.274872205)	1.21E-01	CD69+ CD56lo (log)	7.16E-01
109	Bradyrhizobium	1.97E+00	(.8318281071;4.685551761)	1.23E-01	CD69+ CD56lo (log)	7.19E-01
110	Oribacterium	2.36E-01	(.0376181049;1.485465659)	1.24E-01	CD25+ CD56hi (log)	7.19E-01
111	Prevotella	5.15E-01	(.2202220413;1.202779364)	1.25E-01	CD25+ (log)	7.19E-01
112	Corynebacterium	2.04E+00	(.8207160711;5.046957638)	1.25E-01	NKT CD8+ total (log)	7.19E-01
113	Streptobacillus	2.80E+00	(.7496161483;10.42728068)	1.26E-01	CD56hi (sqrt)	7.19E-01
114	Capnocytophaga	3.16E+00	(.7161382204;13.94970383)	1.29E-01	CD56hi (sqrt)	7.19E-01
115	Lautropia	3.47E-01	(.0887252555;1.360254175)	1.29E-01	CD56hi (sqrt)	7.19E-01
116	Xanthobacteraceae _unclassified	4.49E-01	(.1591453461;1.267089776)	1.30E-01	CD25+ (log)	7.19E-01
117	Atopobium	6.07E-02	(.0016056145;2.293008766)	1.30E-01	CD25+ CD56hi (log)	7.19E-01
118	Neisseriaceae_uncla ssified	3.39E-01	(.082546763;1.391823715)	1.33E-01	NKT CD8+ total (log)	7.20E-01
119	Streptobacillus	3.09E+00	(.701924478;13.63553979)	1.36E-01	CD56hi (sqrt)	7.26E-01
120	Streptobacillus	5.11E-01	(.2112612911;1.237284883)	1.37E-01	CD25+ (log)	7.26E-01
121	Prevotella	1.80E-01	(.018788668;1.72487207)	1.37E-01	CD25+ CD56hi (log)	7.26E-01
122	Sphingomonas	2.07E+00	(.7883034931;5.456098204)	1.39E-01	CD69+ CD56lo (log)	7.28E-01
123	Caulobacter	4.44E-01	(.1507449772;1.308177664)	1.41E-01	NKT CD8+ total (log)	7.29E-01
124	Oribacterium	2.68E+00	(.7165320886;10.05827621)	1.43E-01	CD56hi (sqrt)	7.34E-01
125	Caulobacter	2.17E-01	(.02803967;1.676326385)	1.43E-01	CD25+ CD56lo (log)	7.34E-01
126	Fusobacterium	2.50E+00	(.7338160316;8.489130739)	1.43E-01	CD56hi (sqrt)	7.34E-01
127	Micrococcaceae_un classified	6.02E-01	(.3046256869;1.189692762)	1.44E-01	CD25+ (log)	7.34E-01
128	Lautropia	8.23E-01	(.632127881;1.071029783)	1.47E-01	NK total (sqrt)	7.34E-01
129	Lautropia	5.00E-01	(.1955978449;1.280542189)	1.49E-01	NK total (log)	7.34E-01
130	Comamonadaceae_ unclassified	8.24E-01	(.6335018939;1.071833065)	1.49E-01	CD56lo (sqrt)	7.34E-01
131	Actinobacillus	3.31E-01	(.0736795917;1.487438023)	1.49E-01	CD56hi (sqrt)	7.34E-01
132	Comamonas	4.64E-01	(.1632161228;1.318175736)	1.49E-01	NK total (log)	7.34E-01
133	Pseudomonas	3.06E-01	(.061110742;1.532353519)	1.50E-01	CD69+ CD56hi (log)	7.34E-01
134	Xanthobacteraceae _unclassified	4.87E-01	(.1821229754;1.303202746)	1.52E-01	CD69+ CD56hi (log)	7.34E-01

135	Enterococcaceae_unclassified	3.50E-01	(.0825524195;1.480279714)	1.53E-01	CD69+ CD56lo (log)	7.34E-01
136	Prevotellaceae_unclassified	3.06E-01	(.059396348;1.574369932)	1.56E-01	CD69+ CD56lo (log)	7.34E-01
137	Actinobacillus	3.00E-01	(.0565956036;1.591096613)	1.57E-01	CD69+ (log)	7.34E-01
138	Parvimonas	5.96E+00	(.5018915552;70.79115182)	1.57E-01	CD25+ CD56hi (log)	7.34E-01
139	Porphyromonas	2.13E+00	(.7470026203;6.058868558)	1.57E-01	CD69+ CD56hi (log)	7.34E-01
140	Atopobium	3.98E+00	(.5805670975;27.35090128)	1.60E-01	CD69+ CD56hi (log)	7.34E-01
141	Streptobacillus	1.24E+00	(.9165635458;1.665211059)	1.65E-01	CD56lo (sqrt)	7.46E-01
142	Enterococcaceae_unclassified	3.49E-01	(.0786759125;1.545298477)	1.65E-01	NK total (log)	7.46E-01
143	Capnocytophaga	4.23E-01	(.1236587872;1.447425997)	1.70E-01	CD25+ CD56lo (log)	7.59E-01
144	Actinomyces	6.08E-01	(.2967402553;1.244249356)	1.73E-01	CD69+ CD56hi (log)	7.66E-01
145	Comamonas	5.11E-01	(.1945232008;1.342694215)	1.73E-01	CD56lo (log)	7.66E-01
146	Lautropia	4.54E-01	(.145137206;1.419490101)	1.75E-01	CD56hi (sqrt)	7.66E-01
147	Planococcaceae_unclassified	1.64E+00	(.7987998109;3.384860053)	1.77E-01	CD69+ (log)	7.67E-01
148	Streptobacillus	2.24E+00	(.691698947;7.284916426)	1.78E-01	CD56lo (log)	7.67E-01
149	Absconditabacteriales_(SR1)_ge	2.12E+00	(.7079850686;6.336149374)	1.79E-01	CD69+ CD56hi (log)	7.67E-01
150	Afipia	2.60E+00	(.640239329;10.51816569)	1.82E-01	CD56hi (sqrt)	7.72E-01
151	Lachnoanaerobaculum	2.96E-01	(.049123715;1.783901566)	1.84E-01	NK total (log)	7.76E-01
152	Actinobacillus	4.46E-01	(.1346788817;1.475291891)	1.86E-01	NKT CD8+ total (log)	7.79E-01
153	Fluviicola	3.87E-01	(.094796597;1.583394837)	1.87E-01	NKT CD8+ total (log)	7.79E-01
154	Lautropia	5.41E-01	(.2163273804;1.354955234)	1.90E-01	CD56lo (log)	7.85E-01
155	Actinobacillus	3.31E-01	(.0634828091;1.728487826)	1.90E-01	CD69+ CD56lo (log)	7.85E-01
156	Neisseriaceae_unclassified	1.90E-01	(.0157420151;2.292061579)	1.91E-01	CD25+ CD56hi (log)	7.85E-01
157	Lachnoanaerobaculum	3.15E-01	(.0551093167;1.805651222)	1.95E-01	CD56lo (log)	7.91E-01
158	Absconditabacteriales_(SR1)_ge	2.98E+00	(.5711436515;15.5189001)	1.95E-01	CD56hi (sqrt)	7.91E-01
159	Sphingomonas	1.95E+00	(.7064374687;5.378002744)	1.97E-01	CD69+ (log)	7.91E-01
160	Prevotella	4.56E-01	(.1379961654;1.510123153)	1.99E-01	CD69+ (log)	7.92E-01
161	Lautropia	8.45E-01	(.6529934182;1.093279864)	2.00E-01	CD56lo (sqrt)	7.92E-01
162	Lachnoanaerobaculum	1.76E+00	(.739815262;4.210149888)	2.00E-01	NKT CD8+ total (log)	7.92E-01
163	Parvimonas	2.37E+00	(.6294908221;8.933722857)	2.02E-01	CD56hi (sqrt)	7.92E-01
164	Prevotellaceae_ge	4.91E+00	(.4257554364;56.70270025)	2.02E-01	CD25+ CD56hi (log)	7.92E-01
165	Sphingomonas	8.56E-01	(.673380515;1.088172872)	2.04E-01	NK total (sqrt)	7.92E-01
166	Parvimonas	4.01E-01	(.0979654519;1.643003685)	2.04E-01	CD56lo (log)	7.92E-01
167	Unclassified	1.45E+00	(.813882987;2.597118298)	2.06E-01	NKT CD8+ total (log)	7.92E-01
168	Atopobium	1.51E+00	(.7895758069;2.883217926)	2.13E-01	NK total (sqrt)	8.08E-01
169	Streptobacillus	2.12E+00	(.640474166;7.007851851)	2.19E-01	NK total (log)	8.24E-01
170	Capnocytophaga	2.83E+00	(.5386298199;14.82731427)	2.19E-01	CD56hi (sqrt)	8.24E-01
171	Catonella	8.29E-01	(.61384171;1.12005391)	2.22E-01	NK total (sqrt)	8.27E-01
172	Enterococcaceae_unclassified	4.05E-01	(.0940105464;1.742707998)	2.25E-01	CD69+ (log)	8.32E-01
173	Parvimonas	7.95E-01	(.5474858971;1.153219189)	2.26E-01	CD56lo (sqrt)	8.33E-01
174	Planococcaceae_unclassified	5.07E-01	(.1690029137;1.523705953)	2.27E-01	CD56hi (sqrt)	8.33E-01
175	Fluviicola	2.68E+00	(.542020809;13.26659196)	2.27E-01	CD69+ CD56lo (log)	8.33E-01
176	Comamonas	1.54E+00	(.7636001418;3.102028371)	2.28E-01	NKT CD8+ total (log)	8.33E-01
177	Aquabacterium	2.08E+00	(.6314897836;6.882224528)	2.28E-01	CD56hi (sqrt)	8.33E-01
178	Corynebacterium	2.62E-01	(.0297256148;2.314178848)	2.28E-01	CD25+ CD56hi (log)	8.33E-01
179	Capnocytophaga	6.03E-01	(.2644582756;1.375239272)	2.29E-01	CD25+ (log)	8.33E-01
180	Afipia	5.34E-01	(.1915626044;1.487972682)	2.30E-01	NK total (log)	8.33E-01
181	Flavobacterium	4.54E-01	(.1226786943;1.678190937)	2.36E-01	CD25+ CD56hi (log)	8.33E-01

182	Fusobacterium	6.06E-01	(.2615658488;1.404627786)	2.43E-01	NKT CD8+ total (log)	8.50E-01
183	Prevotella	5.06E-01	(.1602143841;1.595586236)	2.45E-01	CD69+ CD56lo (log)	8.52E-01
184	Catonella	8.40E-01	(.6255891277;1.127467905)	2.45E-01	CD56lo (sqrt)	8.52E-01
185	Veillonella	5.05E-01	(.1591839545;1.602828929)	2.46E-01	CD56hi (sqrt)	8.52E-01
186	Prevotellaceae_ge	2.61E+00	(.5151375457;13.17594391)	2.47E-01	CD69+ CD56hi (log)	8.52E-01
187	Lachnoanaerobaculum	1.89E-01	(.0111655072;3.188397299)	2.48E-01	CD25+ CD56hi (log)	8.52E-01
188	Alloprevotella	2.93E+00	(.4730709106;18.20082544)	2.48E-01	CD69+ CD56hi (log)	8.52E-01
189	Lachnospiraceae_unclassified	5.03E-01	(.1564728978;1.61416987)	2.48E-01	CD69+ CD56lo (log)	8.52E-01
190	Rothia	2.04E+00	(.6039608881;6.893427191)	2.51E-01	CD56hi (sqrt)	8.52E-01
191	Comamonadaceae_unclassified	1.61E+00	(.7151398009;3.606770831)	2.51E-01	CD25+ (log)	8.52E-01
192	Atopobium	1.43E+00	(.7734351294;2.650023396)	2.53E-01	CD56lo (sqrt)	8.52E-01
193	Neisseria	6.38E-01	(.2945774035;1.382617879)	2.55E-01	CD25+ (log)	8.52E-01
194	Absconditabacteriales_(SR1)_ge	2.40E+00	(.5198463041;11.07530842)	2.62E-01	CD56hi (sqrt)	8.60E-01
195	Lautropia	4.23E+00	(.3341041193;53.63011908)	2.65E-01	CD25+ CD56hi (log)	8.67E-01
196	Afipia	8.47E-01	(.631623207;1.135358926)	2.66E-01	NK total (sqrt)	8.67E-01
197	Pasteurellaceae_unclassified	4.06E-01	(.0824657315;1.998657258)	2.68E-01	CD25+ CD56hi (log)	8.67E-01
198	Acinetobacter	4.58E-01	(.1148900563;1.824221605)	2.68E-01	NK total (log)	8.67E-01
199	Actinomyces	6.62E-01	(.3190964817;1.373623455)	2.68E-01	CD69+ (log)	8.67E-01
200	Oxalobacteraceae_unclassified	1.61E+00	(.6928841371;3.733791261)	2.69E-01	CD69+ (log)	8.67E-01
201	Streptobacillus	1.19E+00	(.8764104227;1.605817659)	2.69E-01	NK total (sqrt)	8.67E-01
202	Cardiobacterium	1.24E+00	(.8443803279;1.82643028)	2.71E-01	NK total (sqrt)	8.67E-01
203	Comamonas	8.46E-01	(.6278828433;1.140010673)	2.72E-01	NK total (sqrt)	8.67E-01
204	Catonella	4.23E-01	(.090194916;1.985228799)	2.76E-01	CD25+ CD56hi (log)	8.67E-01
205	Enterococcaceae_unclassified	3.06E+00	(.407700782;22.95771949)	2.77E-01	CD56hi (sqrt)	8.67E-01
206	Acinetobacter	4.77E-01	(.1259529771;1.809985575)	2.77E-01	CD56lo (log)	8.67E-01
207	Cardiobacterium	1.22E+00	(.8493720697;1.762123851)	2.79E-01	CD56lo (sqrt)	8.67E-01
208	Neisseria	1.67E+00	(.6573640773;4.252892471)	2.80E-01	CD56lo (log)	8.67E-01
209	Actinomyces	6.67E-01	(.3195259902;1.393784029)	2.82E-01	CD69+ CD56lo (log)	8.67E-01
210	Lachnospiraceae_unclassified	5.24E-01	(.1606993661;1.709375871)	2.84E-01	CD69+ (log)	8.67E-01
211	Oribacterium	1.75E+00	(.6258006392;4.888261874)	2.86E-01	CD25+ CD56lo (log)	8.67E-01
212	Streptococcus	6.91E-01	(.3494612623;1.366772444)	2.88E-01	CD69+ CD56lo (log)	8.67E-01
213	Fluviicola	2.24E+00	(.5039286992;9.940983108)	2.90E-01	CD25+ CD56lo (log)	8.67E-01
214	Paucibacter	1.99E+00	(.5544925605;7.1475608)	2.91E-01	NKT CD8+ total (log)	8.67E-01
215	Capnocytophaga	5.51E-01	(.1812894436;1.671744273)	2.92E-01	CD69+ CD56hi (log)	8.67E-01
216	Enterococcaceae_unclassified	3.41E+00	(.345929957;33.53228104)	2.94E-01	CD25+ CD56hi (log)	8.67E-01
217	Campylobacter	1.86E+00	(.5834425351;5.936419923)	2.94E-01	CD56lo (log)	8.67E-01
218	Oribacterium	2.21E+00	(.4951564076;9.849987323)	2.99E-01	CD56hi (sqrt)	8.74E-01
219	Oribacterium	5.53E-01	(.1807743144;1.692100212)	2.99E-01	CD69+ CD56hi (log)	8.74E-01
220	Afipia	6.03E-01	(.2323856319;1.567163839)	3.00E-01	CD69+ CD56hi (log)	8.74E-01
221	Prevotellaceae_ge	5.06E-01	(.1394833318;1.835890175)	3.00E-01	CD25+ (log)	8.74E-01
222	Rothia	1.18E+00	(.8643269804;1.600418482)	3.02E-01	NK total (sqrt)	8.74E-01
223	Rothia	1.80E+00	(.5886883875;5.506066679)	3.03E-01	CD56lo (log)	8.74E-01
224	Parvimonas	1.96E+00	(.5424946341;7.045430561)	3.05E-01	NKT CD8+ total (log)	8.74E-01
225	Lachnospiraceae_unclassified	1.82E+00	(.5764293462;5.716669332)	3.08E-01	CD69+ CD56hi (log)	8.74E-01
226	Afipia	2.05E+00	(.5079011382;8.308398897)	3.13E-01	CD56hi (sqrt)	8.81E-01
227	Oxalobacteraceae_unclassified	1.50E+00	(.6784780267;3.328589542)	3.15E-01	CD69+ CD56lo (log)	8.85E-01
228	Fusobacterium	1.84E+00	(.5582605297;6.056957877)	3.17E-01	CD56hi (sqrt)	8.85E-01
229	Cardiobacterium	2.23E+00	(.4633962285;10.71895951)	3.17E-01	NK total (log)	8.85E-01

230	Comamonas	8.70E-01	(.6622168829;1.143141449)	3.18E-01	CD56lo (sqrt)	8.85E-01
231	Fusobacterium	5.78E-01	(.1967436912;1.698182337)	3.19E-01	CD69+ (log)	8.85E-01
232	Sphingomonas	6.54E-01	(.2835586878;1.508117105)	3.19E-01	NK total (log)	8.85E-01
233	Cardiobacterium	2.47E+00	(.4171777326;14.60429847)	3.19E-01	CD56hi (sqrt)	8.85E-01
234	Aquabacterium	5.29E-01	(.1503262437;1.859644145)	3.21E-01	CD69+ CD56hi (log)	8.85E-01
235	Rothia	1.83E+00	(.5496488855;6.118667372)	3.24E-01	NK total (log)	8.85E-01
236	Fluviicola	4.96E-01	(.1228062232;2.001139608)	3.24E-01	NK total (log)	8.85E-01
237	Oxalobacteraceae_ unclassified	1.87E+00	(.5384551921;6.479422731)	3.25E-01	NKT CD8+ total (log)	8.85E-01
238	Bradyrhizobium	5.80E-01	(.1960874531;1.716718526)	3.25E-01	CD25+ CD56lo (log)	8.85E-01
239	Afipia	3.93E-01	(.0608372744;2.536192298)	3.26E-01	CD25+ CD56hi (log)	8.85E-01
240	Rothia	6.74E-01	(.3066790033;1.482001598)	3.27E-01	CD69+ CD56hi (log)	8.85E-01
241	Pasteurellaceae_un classified	4.48E-01	(.0902218454;2.228678909)	3.27E-01	CD56hi (sqrt)	8.85E-01
242	Acinetobacter	1.57E+00	(.6332930819;3.906438216)	3.29E-01	NKT CD8+ total (log)	8.85E-01
243	Sphingomonas	8.89E-01	(.7020608705;1.126667302)	3.31E-01	CD56lo (sqrt)	8.85E-01
244	Neisseria	1.68E+00	(.5908784551;4.756496631)	3.32E-01	NK total (log)	8.85E-01
245	Streptobacillus	6.07E-01	(.2213843494;1.663210875)	3.32E-01	NKT CD8+ total (log)	8.85E-01
246	Xanthobacteraceae _unclassified	2.74E-01	(.0199167027;3.762460411)	3.33E-01	CD25+ CD56hi (log)	8.85E-01
247	Fluviicola	2.15E+00	(.4569211541;10.11869152)	3.33E-01	CD69+ (log)	8.85E-01
248	Absconditabacterial es_(SR1)_ge	5.23E-01	(.141025001;1.940736686)	3.33E-01	NKT CD8+ total (log)	8.85E-01
249	Cardiobacterium	2.06E+00	(.4742919595;8.945446609)	3.35E-01	CD56lo (log)	8.85E-01
250	Rothia	1.15E+00	(.8611883717;1.547975775)	3.37E-01	CD56lo (sqrt)	8.85E-01
251	Paucibacter	1.69E+00	(.5786886295;4.917493358)	3.38E-01	CD69+ CD56lo (log)	8.85E-01
252	Lautropia	4.40E-01	(.0820520488;2.361810653)	3.38E-01	CD69+ CD56hi (log)	8.85E-01
253	Campylobacter	1.15E+00	(.8624339665;1.537260769)	3.39E-01	CD56lo (sqrt)	8.85E-01
254	Streptococcus	7.12E-01	(.3546007052;1.428801912)	3.39E-01	CD69+ (log)	8.85E-01
255	Lachnoanaerobacul um	3.63E-01	(.0449720469;2.92293396)	3.41E-01	CD56hi (sqrt)	8.85E-01
256	Actinobacillus	6.58E-01	(.2762817475;1.565957151)	3.44E-01	CD25+ (log)	8.85E-01
257	Acinetobacter	8.39E-01	(.5799637384;1.212473348)	3.49E-01	NK total (sqrt)	8.85E-01
258	Atopobium	6.81E-01	(.3038487317;1.527599254)	3.52E-01	CD25+ CD56lo (log)	8.85E-01
259	Terrisporobacter	1.71E+00	(.5533674671;5.258921454)	3.52E-01	NKT CD8+ total (log)	8.85E-01
260	Veillonella	6.83E-01	(.3053390053;1.527354403)	3.53E-01	CD25+ (log)	8.85E-01
261	Parvimonas	4.82E-01	(.1031497788;2.254746173)	3.54E-01	NK total (log)	8.85E-01
262	Micrococcaceae_un classified	7.23E-01	(.3635969952;1.43749812)	3.55E-01	CD25+ CD56lo (log)	8.85E-01
263	Comamonas	6.24E-01	(.2278759507;1.706359243)	3.58E-01	CD69+ CD56hi (log)	8.85E-01
264	Parvimonas	1.87E+00	(.4909682734;7.153757986)	3.58E-01	CD56hi (sqrt)	8.85E-01
265	Bradyrhizobium	8.78E-01	(.6656484192;1.158641776)	3.58E-01	NK total (sqrt)	8.85E-01
266	Alloprevotella	2.20E-01	(.0085637691;5.676692664)	3.62E-01	CD56hi (sqrt)	8.85E-01
267	Xanthobacteraceae _unclassified	1.17E+00	(.8329500314;1.648121375)	3.63E-01	CD56lo (sqrt)	8.85E-01
268	Caulobacter	8.04E-01	(.5030813452;1.286391854)	3.64E-01	NK total (sqrt)	8.85E-01
269	Streptococcus	7.58E-01	(.417151903;1.37836609)	3.64E-01	CD25+ CD56lo (log)	8.85E-01
270	Bradyrhizobium	6.38E-01	(.2412131913;1.688760568)	3.66E-01	NK total (log)	8.85E-01
271	Corynebacterium	1.65E+00	(.554149757;4.932206225)	3.67E-01	NK total (log)	8.85E-01
272	Comamonadaceae_ unclassified	1.37E+00	(.68677093;2.748540688)	3.69E-01	NKT CD8+ total (log)	8.85E-01
273	Catonella	1.58E+00	(.5779114907;4.337258769)	3.72E-01	CD69+ CD56lo (log)	8.85E-01
274	Atopobium	2.60E+00	(.3160186945;21.3519091)	3.74E-01	CD56hi (sqrt)	8.85E-01
275	Unclassified	1.32E+00	(.7102905369;2.470761562)	3.77E-01	CD69+ (log)	8.85E-01
276	Corynebacterium	6.08E-01	(.2008311232;1.83862434)	3.78E-01	CD25+ CD56lo (log)	8.85E-01
277	Planococcaceae_un classified	6.34E-01	(.2284315696;1.756972831)	3.80E-01	CD56lo (log)	8.85E-01
278	Pasteurellaceae_un classified	5.90E-01	(.1807277637;1.922906467)	3.81E-01	CD25+ CD56lo (log)	8.85E-01

279	Afipia	6.22E-01	(.2140550848;1.80734794)	3.83E-01	CD56lo (log)	8.85E-01
280	Oxalobacteraceae_unclassified	1.66E+00	(.5263450861;5.262656974)	3.86E-01	CD69+ CD56hi (log)	8.85E-01
281	Methylobacterium-Methylorubrum	1.38E+00	(.6646202123;2.866152368)	3.87E-01	CD25+ CD56lo (log)	8.85E-01
282	Flavobacterium	1.42E+00	(.6407460826;3.138166382)	3.89E-01	CD25+ (log)	8.85E-01
283	Cardiobacterium	2.24E+00	(.3532724206;14.20879504)	3.92E-01	CD56hi (sqrt)	8.85E-01
284	Neisseria	4.01E-01	(.0491192649;3.275471392)	3.94E-01	CD69+ (log)	8.85E-01
285	Aquabacterium	4.98E-01	(.1000648396;2.479032981)	3.95E-01	CD25+ CD56hi (log)	8.85E-01
286	Fusobacterium	6.27E-01	(.2134135344;1.839531721)	3.95E-01	CD69+ CD56lo (log)	8.85E-01
287	Weeksellaceae_unclassified	4.58E-01	(.0751583978;2.78667429)	3.96E-01	CD25+ CD56hi (log)	8.85E-01
288	Aquabacterium	5.34E-01	(.1254639603;2.275908946)	3.97E-01	NK total (log)	8.85E-01
289	Corynebacterium	1.13E+00	(.8565638954;1.477875617)	3.97E-01	NK total (sqrt)	8.85E-01
290	Afipia	8.82E-01	(.6586894288;1.180764262)	3.99E-01	CD56lo (sqrt)	8.85E-01
291	Veillonella	1.22E+00	(.7684116231;1.930902432)	4.01E-01	CD56lo (sqrt)	8.85E-01
292	Neisseria	4.19E-01	(.0541726099;3.240881202)	4.05E-01	CD69+ CD56lo (log)	8.85E-01
293	Sphingomonas	7.09E-01	(.3151169095;1.594782674)	4.06E-01	CD56lo (log)	8.85E-01
294	Pasteurellaceae_unclassified	1.65E+00	(.502202191;5.4462253)	4.08E-01	NKT CD8+ total (log)	8.85E-01
295	Oribacterium	1.43E+00	(.6075601717;3.380172954)	4.11E-01	CD25+ (log)	8.88E-01
296	Acinetobacter	8.63E-01	(.6057397803;1.22863994)	4.13E-01	CD56lo (sqrt)	8.89E-01
297	Atopobium	3.34E+00	(.180931637;61.71598445)	4.17E-01	NK total (log)	8.95E-01
298	Lachnoanaerobaculum	1.59E+00	(.5155064623;4.904170495)	4.20E-01	CD25+ (log)	8.97E-01
299	Porphyromonas	6.64E-01	(.2445867757;1.801915142)	4.21E-01	CD69+ (log)	8.98E-01
300	Veillonella	6.83E-01	(.2681265645;1.73727663)	4.23E-01	CD25+ CD56lo (log)	8.98E-01
301	Porphyromonas	1.53E+00	(.5385331233;4.363350529)	4.24E-01	CD56lo (log)	8.98E-01
302	Campylobacter	1.67E+00	(.4763061837;5.841978215)	4.24E-01	NK total (log)	8.98E-01
303	Prevotellaceae_unclassified	3.80E-01	(.0351703352;4.111218393)	4.26E-01	CD25+ CD56hi (log)	8.98E-01
304	Veillonella	5.27E-01	(.1078114894;2.571961343)	4.28E-01	CD56hi (sqrt)	8.98E-01
305	Actinobacillus	7.24E-01	(.3247741551;1.612735616)	4.29E-01	CD25+ CD56lo (log)	8.98E-01
306	Prevotellaceae_unclassified	4.85E-01	(.0800734827;2.931657856)	4.30E-01	CD69+ CD56hi (log)	8.98E-01
307	Actinomyces	1.24E+00	(.7286330553;2.098839533)	4.31E-01	CD25+ (log)	8.98E-01
308	Paucibacter	1.72E+00	(.4401401011;6.750574129)	4.34E-01	CD56hi (sqrt)	8.98E-01
309	Planococcaceae_unclassified	6.31E-01	(.198739196;2.005468084)	4.35E-01	NK total (log)	8.98E-01
310	Atopobium	2.82E+00	(.2057795891;38.67798223)	4.37E-01	CD56lo (log)	8.99E-01
311	Pasteurellaceae_unclassified	7.63E-01	(.3850827579;1.512299034)	4.39E-01	CD25+ (log)	8.99E-01
312	Parvimonas	8.51E-01	(.566264365;1.279533918)	4.39E-01	NK total (sqrt)	8.99E-01
313	Paucibacter	1.40E+00	(.5939318507;3.316566133)	4.40E-01	CD25+ CD56lo (log)	8.99E-01
314	Lachnoanaerobaculum	8.25E-01	(.502137104;1.355486205)	4.48E-01	NK total (sqrt)	9.08E-01
315	Neisseria	5.11E-01	(.0896231022;2.910044295)	4.49E-01	CD25+ CD56hi (log)	9.08E-01
316	Aquabacterium	8.69E-01	(.6049273628;1.249334656)	4.49E-01	NK total (sqrt)	9.08E-01
317	Porphyromonas	6.87E-01	(.2599395071;1.817975922)	4.50E-01	CD69+ CD56lo (log)	9.08E-01
318	Pseudomonas	1.60E+00	(.471005847;5.454176255)	4.50E-01	CD69+ CD56lo (log)	9.08E-01
319	Campylobacter	6.97E-01	(.2718201673;1.785362635)	4.52E-01	CD25+ (log)	9.08E-01
320	Corynebacterium	1.50E+00	(.5212308602;4.290687903)	4.54E-01	CD56lo (log)	9.08E-01
321	Cardiobacterium	6.48E-01	(.2061418454;2.039122879)	4.59E-01	CD25+ (log)	9.10E-01
322	Weeksellaceae_unclassified	1.56E+00	(.4805114526;5.038591395)	4.61E-01	CD56hi (sqrt)	9.12E-01
323	Flavobacterium	1.49E+00	(.5122229389;4.352735337)	4.63E-01	CD25+ CD56lo (log)	9.12E-01
324	Micrococcaceae_unclassified	8.62E-01	(.5803922372;1.281039717)	4.63E-01	CD56lo (sqrt)	9.12E-01
325	Cardiobacterium	4.01E-01	(.0345963195;4.648029309)	4.65E-01	CD25+ CD56hi (log)	9.12E-01

326	Micrococcaceae_unclassified	8.54E-01	(.5577571056;1.306353118)	4.66E-01	NK total (sqrt)	9.12E-01
327	Neisseria	6.60E-01	(.2134377133;2.039352274)	4.70E-01	NKT CD8+ total (log)	9.16E-01
328	Weeksellaceae_unclassified	7.40E-01	(.327196553;1.675523931)	4.71E-01	CD25+ CD56lo (log)	9.16E-01
329	Campylobacter	1.12E+00	(.8243719076;1.517431834)	4.72E-01	NK total (sqrt)	9.16E-01
330	Alloprevotella	1.44E+00	(.5300236485;3.905814561)	4.75E-01	CD25+ (log)	9.17E-01
331	Enterococcaceae_unclassified	2.08E+00	(.2763653868;15.65817829)	4.77E-01	CD56hi (sqrt)	9.18E-01
332	Unclassified	1.24E+00	(.6865469762;2.225838766)	4.80E-01	CD69+ CD56lo (log)	9.21E-01
333	Pasteurellaceae_unclassified	5.65E-01	(.1146519861;2.784905208)	4.83E-01	CD69+ CD56hi (log)	9.24E-01
334	Aquabacterium	1.33E+00	(.5987648621;2.947625371)	4.85E-01	NKT CD8+ total (log)	9.25E-01
335	Atopobium	5.77E-01	(.1228827916;2.711074539)	4.86E-01	CD25+ (log)	9.25E-01
336	Actinomyces	1.10E+00	(.8468441762;1.417870354)	4.87E-01	NK total (sqrt)	9.25E-01
337	Alloprevotella	1.49E+00	(.4855371511;4.556877491)	4.87E-01	CD25+ CD56lo (log)	9.25E-01
338	Acinetobacter	6.50E-01	(.1907455616;2.212149444)	4.90E-01	CD69+ CD56hi (log)	9.25E-01
339	Sphingomonas	1.78E+00	(.3435071209;9.25533311)	4.91E-01	CD56hi (sqrt)	9.25E-01
340	Porphyromonas	1.10E+00	(.8420055099;1.430157511)	4.92E-01	CD56lo (sqrt)	9.25E-01
341	Rothia	6.13E-01	(.1515262284;2.476951797)	4.92E-01	CD25+ CD56lo (log)	9.25E-01
342	Lachnoanaerobaculum	6.84E-01	(.2304162035;2.028693221)	4.93E-01	CD25+ CD56lo (log)	9.25E-01
343	Actinomyces	1.37E+00	(.5524974765;3.409844999)	4.95E-01	CD56hi (sqrt)	9.25E-01
344	Paucibacter	1.75E+00	(.3498634436;8.760111813)	4.95E-01	CD56hi (sqrt)	9.25E-01
345	Fusobacterium	7.42E-01	(.3131701088;1.756350075)	4.97E-01	CD69+ CD56hi (log)	9.25E-01
346	Actinomyces	1.09E+00	(.853571255;1.384502682)	4.99E-01	CD56lo (sqrt)	9.25E-01
347	Aquabacterium	6.30E-01	(.1639809998;2.421868185)	5.01E-01	CD56lo (log)	9.25E-01
348	Streptobacillus	7.09E-01	(.2593342981;1.936087531)	5.02E-01	CD69+ CD56hi (log)	9.25E-01
349	Sphingomonas	1.67E+00	(.372075229;7.492955762)	5.03E-01	CD56hi (sqrt)	9.25E-01
350	Fluviicola	1.89E+00	(.2907459542;12.27838369)	5.05E-01	CD25+ (log)	9.25E-01
351	Xanthobacteraceae_unclassified	1.58E+00	(.4124862009;6.033250096)	5.05E-01	CD56hi (sqrt)	9.25E-01
352	Veillonella	7.54E-01	(.3271594661;1.739333408)	5.08E-01	CD69+ CD56hi (log)	9.25E-01
353	Lachnoanaerobaculum	8.42E-01	(.5047928187;1.403279109)	5.09E-01	CD56lo (sqrt)	9.25E-01
354	Lautropia	7.63E-01	(.3426933563;1.700403801)	5.09E-01	NKT CD8+ total (log)	9.25E-01
355	Xanthobacteraceae_unclassified	1.56E+00	(.4163298219;5.826869166)	5.10E-01	CD56lo (log)	9.25E-01
356	Methylobacterium-Methylorubrum	1.47E+00	(.4658282822;4.642296475)	5.11E-01	CD69+ (log)	9.25E-01
357	Caulobacter	8.46E-01	(.5102958778;1.401098404)	5.15E-01	CD56lo (sqrt)	9.25E-01
358	Selenomonas	1.44E+00	(.4795644583;4.32656766)	5.15E-01	CD56lo (log)	9.25E-01
359	Selenomonas	5.48E-01	(.0891646426;3.366984639)	5.16E-01	CD69+ (log)	9.25E-01
360	Capnocytophaga	1.51E+00	(.4361802341;5.192929795)	5.18E-01	CD56lo (log)	9.25E-01
361	Selenomonas	6.48E-01	(.1738765404;2.41420289)	5.18E-01	CD25+ CD56lo (log)	9.25E-01
362	Prevotellaceae_unclassified	1.47E+00	(.4509295911;4.786084519)	5.23E-01	NK total (log)	9.25E-01
363	Bradyrhizobium	9.17E-01	(.7014303274;1.19819614)	5.25E-01	CD56lo (sqrt)	9.25E-01
364	Fluviicola	8.87E-01	(.614184469;1.282183522)	5.25E-01	NK total (sqrt)	9.25E-01
365	Planococcaceae_unclassified	1.42E+00	(.483850705;4.146358165)	5.25E-01	CD25+ CD56hi (log)	9.25E-01
366	Neisseriaceae_unclassified	6.44E-01	(.1635848622;2.536681725)	5.29E-01	CD25+ CD56lo (log)	9.25E-01
367	Xanthobacteraceae_unclassified	1.12E+00	(.7867621185;1.592696179)	5.31E-01	NK total (sqrt)	9.25E-01
368	Methylobacterium-Methylorubrum	1.41E+00	(.4811818803;4.126975749)	5.32E-01	CD69+ CD56lo (log)	9.25E-01
369	Planococcaceae_unclassified	7.40E-01	(.2866289621;1.910677501)	5.34E-01	CD56hi (sqrt)	9.25E-01

370	Porphyromonas	7.75E-01	(.3457750378;1.736439063)	5.36E-01	CD25+ (log)	9.25E-01
371	Prevotellaceae_unclassified	1.41E+00	(.472167254;4.231869151)	5.36E-01	CD56lo (log)	9.25E-01
372	Bradyrhizobium	8.14E-01	(.4229659592;1.56653927)	5.38E-01	CD25+ (log)	9.25E-01
373	Veillonella	1.64E+00	(.3391276569;7.895007666)	5.40E-01	CD56lo (log)	9.25E-01
374	Afipia	1.56E+00	(.376962676;6.449367323)	5.40E-01	CD25+ CD56lo (log)	9.25E-01
375	Flavobacterium	1.53E+00	(.387477383;6.06591513)	5.43E-01	CD69+ CD56hi (log)	9.25E-01
376	Enterococcaceae_unclassified	6.91E-01	(.2104139259;2.27216608)	5.43E-01	NKT CD8+ total (log)	9.25E-01
377	Corynebacterium	1.43E+00	(.4488014041;4.56541698)	5.44E-01	CD69+ CD56lo (log)	9.25E-01
378	Paucibacter	9.18E-01	(.6957950691;1.211352983)	5.46E-01	NK total (sqrt)	9.25E-01
379	Alloprevotella	6.02E-01	(.1163326007;3.119270393)	5.46E-01	CD69+ CD56lo (log)	9.25E-01
380	Bradyrhizobium	7.43E-01	(.2834480523;1.948523439)	5.46E-01	CD56lo (log)	9.25E-01
381	Catonella	1.27E+00	(.5763472331;2.811676526)	5.51E-01	CD25+ CD56lo (log)	9.25E-01
382	Absconditabacteriales_(SR1)_ge	6.35E-01	(.142477597;2.834304753)	5.52E-01	CD69+ CD56lo (log)	9.25E-01
383	Selenomonas	5.93E-01	(.1039180979;3.384457852)	5.57E-01	CD69+ CD56lo (log)	9.26E-01
384	Terrisporobacter	5.59E-01	(.0792914102;3.934964753)	5.59E-01	CD69+ CD56hi (log)	9.27E-01
385	Prevotellaceae_unclassified	1.18E+00	(.6806304063;2.036901348)	5.59E-01	CD25+ CD56lo (log)	9.27E-01
386	Neisseria	1.08E+00	(.8428054375;1.371918157)	5.59E-01	CD56lo (sqrt)	9.27E-01
387	Lautropia	7.00E-01	(.2110979693;2.322112338)	5.60E-01	CD25+ CD56lo (log)	9.27E-01
388	Absconditabacteriales_(SR1)_ge	1.42E+00	(.4354881178;4.639864682)	5.60E-01	NK total (log)	9.27E-01
389	Catonella	1.35E+00	(.4916361558;3.687641185)	5.63E-01	CD69+ (log)	9.27E-01
390	Afipia	1.41E+00	(.4378637308;4.529729937)	5.66E-01	NKT CD8+ total (log)	9.27E-01
391	Absconditabacteriales_(SR1)_ge	6.36E-01	(.1350076476;2.992319706)	5.66E-01	CD69+ (log)	9.27E-01
392	Porphyromonas	5.74E-01	(.0860394865;3.830210175)	5.67E-01	CD56hi (sqrt)	9.27E-01
393	Bradyrhizobium	1.29E+00	(.5358992248;3.121767909)	5.67E-01	NKT CD8+ total (log)	9.27E-01
394	Micrococcaceae_unclassified	7.09E-01	(.2162345802;2.325230493)	5.70E-01	CD56lo (log)	9.27E-01
395	Veillonella	1.14E+00	(.7308368143;1.765520216)	5.71E-01	NK total (sqrt)	9.27E-01
396	Alloprevotella	6.40E-01	(.1353155904;3.024101826)	5.73E-01	CD69+ (log)	9.27E-01
397	Micrococcaceae_unclassified	7.00E-01	(.2016814877;2.426638788)	5.73E-01	NK total (log)	9.27E-01
398	Absconditabacteriales_(SR1)_ge	1.38E+00	(.4523294461;4.188236485)	5.74E-01	CD56lo (log)	9.27E-01
399	Aquabacterium	9.07E-01	(.6450594696;1.275266878)	5.74E-01	CD56lo (sqrt)	9.27E-01
400	Catonella	7.60E-01	(.2914121941;1.982306651)	5.75E-01	CD69+ CD56hi (log)	9.27E-01
401	Comamonas	1.22E+00	(.6054265639;2.466272577)	5.76E-01	CD25+ (log)	9.27E-01
402	Prevotellaceae_unclassified	6.42E-01	(.1351897204;3.05046662)	5.77E-01	CD56hi (sqrt)	9.27E-01
403	Pasteurellaceae_unclassified	6.41E-01	(.1312061019;3.130762633)	5.83E-01	CD56lo (log)	9.27E-01
404	Selenomonas	1.39E+00	(.4294250767;4.49332543)	5.83E-01	NK total (log)	9.27E-01
405	Methylobacterium-Methylorubrum	7.49E-01	(.2642332765;2.121430277)	5.86E-01	CD69+ CD56hi (log)	9.27E-01
406	Selenomonas	1.08E+00	(.8112806748;1.447274083)	5.87E-01	CD56lo (sqrt)	9.27E-01
407	Caulobacter	2.86E-01	(.003145784;26.08593853)	5.87E-01	CD25+ CD56hi (log)	9.27E-01
408	Lachnospiraceae_unclassified	4.75E-01	(.0315398133;7.1642657)	5.91E-01	CD25+ CD56hi (log)	9.27E-01
409	Porphyromonas	7.91E-01	(.333333459;1.876055517)	5.94E-01	CD25+ CD56lo (log)	9.27E-01
410	Caulobacter	6.60E-01	(.1427966984;3.05196857)	5.95E-01	CD69+ CD56lo (log)	9.27E-01
411	Catonella	1.26E+00	(.5253754868;3.029637703)	6.03E-01	CD25+ (log)	9.35E-01
412	Campylobacter	7.69E-01	(.2851786852;2.072061346)	6.03E-01	CD25+ CD56lo (log)	9.35E-01
413	Pasteurellaceae_unclassified	6.37E-01	(.1140483142;3.559463357)	6.08E-01	NK total (log)	9.37E-01
414	Corynebacterium	1.08E+00	(.8130305606;1.424426357)	6.08E-01	CD56lo (sqrt)	9.37E-01

415	Porphyromonas	1.31E+00	(.4631892376;3.72352046)	6.08E-01	NK total (log)	9.37E-01
416	Fusobacterium	1.28E+00	(.4969204246;3.299396667)	6.09E-01	CD56lo (log)	9.37E-01
417	Unclassified	7.34E-01	(.2235340894;2.409635124)	6.10E-01	CD25+ CD56hi (log)	9.37E-01
418	Comamonadaceae_unclassified	7.83E-01	(.306254324;2.00391503)	6.10E-01	CD69+ CD56hi (log)	9.37E-01
419	Corynebacterium	1.39E+00	(.3885226197;4.986940005)	6.12E-01	CD69+ (log)	9.37E-01
420	Fluviicola	6.64E-01	(.1369453136;3.221653404)	6.12E-01	CD56lo (log)	9.37E-01
421	Pseudomonas	1.41E+00	(.3779181618;5.226200057)	6.12E-01	CD69+ (log)	9.37E-01
422	Bradyrhizobium	6.35E-01	(.1097107128;3.678515401)	6.13E-01	CD25+ CD56hi (log)	9.37E-01
423	Fusobacterium	1.07E+00	(.8321277899;1.365250379)	6.14E-01	CD56lo (sqrt)	9.37E-01
424	Rothia	1.46E+00	(.3375221446;6.291795025)	6.14E-01	CD56hi (sqrt)	9.37E-01
425	Neisseriaceae_unclassified	1.80E+00	(.1836458629;17.60008519)	6.14E-01	CD56hi (sqrt)	9.37E-01
426	Paucibacter	9.33E-01	(.7096102423;1.225785779)	6.17E-01	CD56lo (sqrt)	9.37E-01
427	Corynebacterium	1.28E+00	(.4846081627;3.381146384)	6.18E-01	CD69+ CD56hi (log)	9.37E-01
428	Planococcaceae_unclassified	9.36E-01	(.7191561297;1.2172014)	6.20E-01	CD56lo (sqrt)	9.37E-01
429	Capnocytophaga	1.40E+00	(.3667088505;5.376394409)	6.20E-01	NK total (log)	9.37E-01
430	Weeksellaceae_unclassified	9.42E-01	(.7416466717;1.196493967)	6.24E-01	NK total (sqrt)	9.37E-01
431	Methylobacterium-Methylorubrum	7.77E-01	(.2811802464;2.146468176)	6.26E-01	NKT CD8+ total (log)	9.37E-01
432	Enterococcaceae_unclassified	1.26E+00	(.4935797305;3.214092044)	6.29E-01	CD25+ (log)	9.37E-01
433	Campylobacter	1.33E+00	(.4160534988;4.256450652)	6.30E-01	CD56hi (sqrt)	9.37E-01
434	Fusobacterium	1.27E+00	(.4783216541;3.358182525)	6.34E-01	NK total (log)	9.37E-01
435	Neisseria	1.07E+00	(.8170604171;1.391575208)	6.37E-01	NK total (sqrt)	9.37E-01
436	Pasteurellaceae_unclassified	9.06E-01	(.5984422981;1.371411846)	6.41E-01	CD56lo (sqrt)	9.37E-01
437	Xanthobacteraceae_unclassified	1.38E+00	(.351574338;5.426381061)	6.44E-01	NKT CD8+ total (log)	9.38E-01
438	Streptococcus	1.30E+00	(.4302445056;3.904112015)	6.45E-01	CD25+ CD56hi (log)	9.38E-01
439	Terrisporobacter	6.34E-01	(.0879216096;4.575135385)	6.52E-01	CD56hi (sqrt)	9.45E-01
440	Xanthobacteraceae_unclassified	1.36E+00	(.3539039668;5.246614041)	6.53E-01	NK total (log)	9.45E-01
441	Paucibacter	7.95E-01	(.2907918537;2.171091246)	6.54E-01	NK total (log)	9.45E-01
442	Pasteurellaceae_unclassified	9.03E-01	(.578715033;1.409692286)	6.54E-01	NK total (sqrt)	9.45E-01
443	Lautropia	1.18E+00	(.5564235077;2.522137774)	6.60E-01	CD25+ (log)	9.49E-01
444	Pasteurellaceae_unclassified	7.50E-01	(.2064382956;2.725508814)	6.62E-01	CD56hi (sqrt)	9.50E-01
445	Neisseriaceae_unclassified	1.57E+00	(.2065084314;11.91699006)	6.63E-01	CD69+ CD56hi (log)	9.50E-01
446	Afipia	1.21E+00	(.5165904972;2.819929526)	6.64E-01	CD25+ (log)	9.50E-01
447	Oribacterium	1.29E+00	(.4044996215;4.116368997)	6.67E-01	NKT CD8+ total (log)	9.50E-01
448	Selenomonas	1.30E+00	(.3952228769;4.267897504)	6.67E-01	NKT CD8+ total (log)	9.50E-01
449	Terrisporobacter	1.12E+00	(.6686230412;1.873422111)	6.68E-01	CD56lo (sqrt)	9.50E-01
450	Prevotellaceae_ge	1.62E+00	(.1773474938;14.83898842)	6.68E-01	CD56hi (sqrt)	9.50E-01
451	Weeksellaceae_unclassified	9.53E-01	(.765361842;1.187067123)	6.68E-01	CD56lo (sqrt)	9.50E-01
452	Micrococcaceae_unclassified	7.86E-01	(.257605259;2.39729773)	6.72E-01	NKT CD8+ total (log)	9.50E-01
453	Methylobacterium-Methylorubrum	1.19E+00	(.5256490554;2.708164612)	6.73E-01	CD25+ (log)	9.50E-01
454	Paucibacter	1.16E+00	(.5731389955;2.366897554)	6.73E-01	CD25+ (log)	9.50E-01
455	Prevotella	9.30E-01	(.6616216773;1.307724098)	6.77E-01	NK total (sqrt)	9.50E-01
456	Rothia	6.00E-01	(.0532067876;6.756761351)	6.79E-01	CD25+ CD56hi (log)	9.50E-01
457	Flavobacterium	1.07E+00	(.7669142208;1.502132665)	6.80E-01	CD56lo (sqrt)	9.50E-01

458	Comamonadaceae_unclassified	1.31E+00	(.3634394907;4.719316634)	6.80E-01	CD25+ CD56lo (log)	9.50E-01
459	Fusobacterium	1.05E+00	(.8211380061;1.352344708)	6.81E-01	NK total (sqrt)	9.50E-01
460	Actinomyces	7.71E-01	(.2212611352;2.688236372)	6.83E-01	CD25+ CD56hi (log)	9.50E-01
461	Acinetobacter	1.64E+00	(.1501840651;17.991307)	6.84E-01	CD25+ CD56hi (log)	9.50E-01
462	Terrisporobacter	1.11E+00	(.6680792401;1.84797238)	6.85E-01	NK total (sqrt)	9.50E-01
463	Oxalobacteraceae_unclassified	1.22E+00	(.4607713119;3.253166952)	6.85E-01	CD25+ (log)	9.50E-01
464	Bradyrhizobium	8.25E-01	(.3250535931;2.092355372)	6.85E-01	CD69+ CD56hi (log)	9.50E-01
465	Prevotella	7.83E-01	(.236622863;2.591944591)	6.89E-01	NK total (log)	9.50E-01
466	Weeksellaceae_unclassified	1.20E+00	(.4925860996;2.91165681)	6.91E-01	NKT CD8+ total (log)	9.50E-01
467	Prevotellaceae_ge	1.49E+00	(.2084573517;10.60878118)	6.92E-01	CD56hi (sqrt)	9.50E-01
468	Flavobacterium	1.22E+00	(.4497746705;3.323669254)	6.94E-01	CD69+ (log)	9.50E-01
469	Pasteurellaceae_unclassified	7.31E-01	(.1510498031;3.534305173)	6.96E-01	CD69+ (log)	9.50E-01
470	Porphyromonas	1.05E+00	(.8080066561;1.373628998)	7.00E-01	NK total (sqrt)	9.50E-01
471	Lachnospiraceae_unclassified	8.69E-01	(.4227453664;1.785833163)	7.02E-01	CD25+ (log)	9.50E-01
472	Parvimonas	1.29E+00	(.3408819926;4.903159308)	7.06E-01	CD69+ CD56hi (log)	9.52E-01
473	Flavobacterium	1.19E+00	(.4708853113;3.022807397)	7.10E-01	CD69+ CD56lo (log)	9.56E-01
474	Caulobacter	1.57E+00	(.1443847039;16.99529867)	7.12E-01	CD56hi (sqrt)	9.57E-01
475	Sphingomonas	7.85E-01	(.2153446809;2.864828084)	7.15E-01	CD69+ CD56hi (log)	9.58E-01
476	Terrisporobacter	7.61E-01	(.1762791623;3.287006484)	7.15E-01	CD25+ CD56lo (log)	9.58E-01
477	Oxalobacteraceae_unclassified	1.31E+00	(.3039946749;5.677665076)	7.15E-01	CD25+ CD56lo (log)	9.58E-01
478	Lachnospiraceae_unclassified	1.40E+00	(.2239197519;8.811706381)	7.17E-01	CD56hi (sqrt)	9.58E-01
479	Absconditabacteriales_(SR1)_ge	1.50E+00	(.1614974547;13.93713687)	7.21E-01	CD25+ CD56hi (log)	9.59E-01
480	Streptococcus	8.94E-01	(.4763508017;1.676466526)	7.26E-01	NKT CD8+ total (log)	9.64E-01
481	Flavobacterium	1.23E+00	(.3897534635;3.861786433)	7.27E-01	CD56lo (log)	9.64E-01
482	Actinomyces	7.83E-01	(.1931330397;3.172149378)	7.32E-01	CD56hi (sqrt)	9.67E-01
483	Veillonella	1.32E+00	(.2693104977;6.474053409)	7.32E-01	NK total (log)	9.67E-01
484	Paucibacter	8.40E-01	(.3085151208;2.287228507)	7.33E-01	CD56lo (log)	9.67E-01
485	Porphyromonas	7.42E-01	(.1334928296;4.127458674)	7.34E-01	CD56hi (sqrt)	9.67E-01
486	Cardiobacterium	7.95E-01	(.2112842861;2.993004181)	7.35E-01	CD69+ CD56hi (log)	9.67E-01
487	Neisseriaceae_unclassified	9.39E-01	(.6490486767;1.358403666)	7.38E-01	NK total (sqrt)	9.67E-01
488	Planococcaceae_unclassified	9.53E-01	(.7178604416;1.264925655)	7.39E-01	NK total (sqrt)	9.67E-01
489	Sphingomonas	1.22E+00	(.3708380534;4.0251119)	7.42E-01	CD25+ CD56lo (log)	9.67E-01
490	Streptococcus	1.03E+00	(.8741161676;1.207333819)	7.44E-01	NK total (sqrt)	9.67E-01
491	Weeksellaceae_unclassified	1.23E+00	(.3560351691;4.215664331)	7.47E-01	CD69+ CD56hi (log)	9.70E-01
492	Unclassified	8.80E-01	(.4046653813;1.914627351)	7.48E-01	CD56hi (sqrt)	9.70E-01
493	Caulobacter	1.46E+00	(.1399525612;15.27389061)	7.51E-01	CD56hi (sqrt)	9.70E-01
494	Caulobacter	7.77E-01	(.162869823;3.702822482)	7.51E-01	NK total (log)	9.70E-01
495	Comamonas	8.38E-01	(.280615185;2.503154574)	7.52E-01	CD25+ CD56lo (log)	9.70E-01
496	Caulobacter	7.72E-01	(.1468127795;4.056801127)	7.60E-01	CD56lo (log)	9.76E-01
497	Selenomonas	1.05E+00	(.7755061126;1.416023504)	7.61E-01	NK total (sqrt)	9.76E-01
498	Absconditabacteriales_(SR1)_ge	1.05E+00	(.7718205348;1.425242246)	7.61E-01	CD56lo (sqrt)	9.76E-01
499	Neisseriaceae_unclassified	6.68E-01	(.0493460552;9.033901971)	7.61E-01	CD69+ (log)	9.76E-01
500	Lachnospiraceae_unclassified	1.25E+00	(.2906580697;5.411593021)	7.61E-01	CD56hi (sqrt)	9.76E-01
501	Comamonadaceae_unclassified	7.78E-01	(.1536856821;3.938880875)	7.62E-01	CD25+ CD56hi (log)	9.76E-01

502	Streptococcus	1.16E+00	(.4341926912;3.125692102)	7.62E-01	CD56hi (sqrt)	9.76E-01
503	Corynebacterium	7.78E-01	(.1506396788;4.017855234)	7.64E-01	CD56hi (sqrt)	9.76E-01
504	Selenomonas	8.17E-01	(.2157581754;3.097106114)	7.67E-01	CD69+ CD56hi (log)	9.76E-01
505	Parvimonas	7.77E-01	(.145695875;4.13978139)	7.67E-01	CD69+ CD56lo (log)	9.76E-01
506	Flavobacterium	1.05E+00	(.7407799564;1.50059221)	7.69E-01	NK total (sqrt)	9.76E-01
507	Fluviicola	9.40E-01	(.6219786595;1.421198225)	7.70E-01	CD56lo (sqrt)	9.76E-01
508	Actinobacillus	1.19E+00	(.3684857804;3.843212217)	7.71E-01	CD56lo (log)	9.76E-01
509	Micrococcaceae_unclassified	1.27E+00	(.254807602;6.318385702)	7.71E-01	CD69+ (log)	9.76E-01
510	Pseudomonas	7.63E-01	(.1216758628;4.786934323)	7.73E-01	CD56hi (sqrt)	9.76E-01
511	Caulobacter	8.16E-01	(.2046813785;3.253321603)	7.73E-01	CD69+ CD56hi (log)	9.76E-01
512	Oribacterium	8.52E-01	(.2849529595;2.544859729)	7.74E-01	CD69+ (log)	9.76E-01
513	Parvimonas	7.83E-01	(.1462278264;4.187566148)	7.74E-01	CD69+ (log)	9.76E-01
514	Flavobacterium	1.20E+00	(.3440461061;4.172274393)	7.76E-01	NK total (log)	9.76E-01
515	Campylobacter	8.82E-01	(.3704039513;2.101305161)	7.77E-01	NKT CD8+ total (log)	9.76E-01
516	Lachnoanaerobaculum	8.80E-01	(.3624022324;2.136703569)	7.78E-01	CD69+ CD56hi (log)	9.76E-01
517	Micrococcaceae_unclassified	1.17E+00	(.3984884942;3.418114592)	7.78E-01	CD69+ CD56hi (log)	9.76E-01
518	Prevotellaceae_unclassified	8.14E-01	(.1937779146;3.420839159)	7.79E-01	CD56hi (sqrt)	9.76E-01
519	Neisseriaceae_unclassified	9.51E-01	(.6715608604;1.347728347)	7.79E-01	CD56lo (sqrt)	9.76E-01
520	Streptococcus	1.09E+00	(.5941033571;1.99606769)	7.83E-01	CD69+ CD56hi (log)	9.76E-01
521	Pseudomonas	5.93E-01	(.0140168221;25.0478226)	7.84E-01	CD25+ CD56hi (log)	9.76E-01
522	Terrisporobacter	8.12E-01	(.1815214335;3.629412615)	7.85E-01	CD69+ (log)	9.76E-01
523	Catonella	8.20E-01	(.1884405946;3.569326598)	7.92E-01	CD56hi (sqrt)	9.76E-01
524	Cardiobacterium	8.60E-01	(.2816295205;2.628097217)	7.92E-01	NKT CD8+ total (log)	9.76E-01
525	Unclassified	1.03E+00	(.8469038513;1.243413414)	7.92E-01	NK total (sqrt)	9.76E-01
526	Lachnospiraceae_unclassified	8.32E-01	(.2032126998;3.405258512)	7.98E-01	NK total (log)	9.76E-01
527	Lachnoanaerobaculum	1.21E+00	(.2817250674;5.192432531)	7.98E-01	CD69+ (log)	9.76E-01
528	Atopobium	1.35E+00	(.1330613146;13.72129208)	7.99E-01	CD69+ CD56lo (log)	9.76E-01
529	Fluviicola	1.56E+00	(.0487899245;49.94559105)	8.01E-01	CD56hi (sqrt)	9.76E-01
530	Micrococcaceae_unclassified	1.22E+00	(.2548673837;5.862470632)	8.02E-01	CD69+ CD56lo (log)	9.76E-01
531	Weeksellaceae_unclassified	1.17E+00	(.341312962;4.00117849)	8.04E-01	CD56hi (sqrt)	9.76E-01
532	Lachnoanaerobaculum	1.18E+00	(.3101239752;4.520429758)	8.05E-01	CD69+ CD56lo (log)	9.76E-01
533	Prevotella	1.13E+00	(.4274364608;2.972200935)	8.09E-01	CD25+ CD56lo (log)	9.76E-01
534	Atopobium	1.35E+00	(.1144398329;15.86954249)	8.13E-01	CD69+ (log)	9.76E-01
535	Flavobacterium	1.13E+00	(.4189910561;3.021467389)	8.15E-01	NKT CD8+ total (log)	9.76E-01
536	Prevotellaceae_ge	9.43E-01	(.5790233267;1.537210044)	8.15E-01	CD56lo (sqrt)	9.76E-01
537	Capnocytophaga	1.04E+00	(.750141153;1.439581372)	8.17E-01	CD56lo (sqrt)	9.76E-01
538	Sphingomonas	9.06E-01	(.3881499776;2.113753477)	8.19E-01	NKT CD8+ total (log)	9.76E-01
539	Prevotellaceae_ge	9.41E-01	(.5525939205;1.602466945)	8.23E-01	NK total (sqrt)	9.76E-01
540	Absconditabacteriales_(SR1)_ge	1.04E+00	(.7513099313;1.432042956)	8.24E-01	NK total (sqrt)	9.76E-01
541	Aquabacterium	8.58E-01	(.2206137625;3.335371749)	8.25E-01	CD25+ CD56lo (log)	9.76E-01
542	Neisseriaceae_unclassified	7.61E-01	(.0645823369;8.974189178)	8.28E-01	CD69+ CD56lo (log)	9.76E-01
543	Oribacterium	1.15E+00	(.3256686383;4.031682372)	8.32E-01	NK total (log)	9.76E-01
544	Neisseriaceae_unclassified	1.15E+00	(.3152747141;4.196236547)	8.32E-01	CD56lo (log)	9.76E-01
545	Porphyromonas	8.90E-01	(.3035746188;2.610649452)	8.32E-01	NKT CD8+ total (log)	9.76E-01
546	Neisseria	1.20E+00	(.2159629568;6.673930076)	8.35E-01	CD56hi (sqrt)	9.76E-01
547	Comamonas	8.33E-01	(.1500246397;4.628313527)	8.35E-01	CD25+ CD56hi (log)	9.76E-01

548	Bradyrhizobium	8.60E-01	(.208480022;3.550563449)	8.35E-01	CD56hi (sqrt)	9.76E-01
549	Xanthobacteraceae_unclassified	1.11E+00	(.4133709848;2.976161986)	8.37E-01	CD69+ CD56lo (log)	9.76E-01
550	Catonella	1.15E+00	(.3101209045;4.243195154)	8.37E-01	CD56hi (sqrt)	9.76E-01
551	Lautropia	1.12E+00	(.3851584026;3.232361579)	8.40E-01	CD69+ CD56lo (log)	9.76E-01
552	Streptococcus	1.06E+00	(.575926517;1.967881294)	8.42E-01	NK total (log)	9.76E-01
553	Afipia	1.14E+00	(.3200122318;4.044834222)	8.42E-01	CD69+ (log)	9.76E-01
554	Lachnospiraceae_unclassified	9.61E-01	(.6505853792;1.419894629)	8.42E-01	NK total (sqrt)	9.76E-01
555	Caulobacter	8.54E-01	(.1737924003;4.194600138)	8.46E-01	CD69+ (log)	9.76E-01
556	Weeksellaceae_unclassified	9.12E-01	(.3539734281;2.351362598)	8.49E-01	NK total (log)	9.76E-01
557	Streptococcus	1.05E+00	(.6503454172;1.68628519)	8.49E-01	CD25+ (log)	9.76E-01
558	Actinomyces	1.12E+00	(.3536678454;3.521267579)	8.52E-01	NK total (log)	9.76E-01
559	Xanthobacteraceae_unclassified	1.14E+00	(.2920521928;4.426965274)	8.53E-01	CD56hi (sqrt)	9.76E-01
560	Paucibacter	1.09E+00	(.4341619153;2.728078104)	8.57E-01	CD69+ CD56hi (log)	9.76E-01
561	Aquabacterium	9.27E-01	(.4000273201;2.146246573)	8.59E-01	CD25+ (log)	9.76E-01
562	Lachnospiraceae_unclassified	8.88E-01	(.235550743;3.345345127)	8.60E-01	CD56lo (log)	9.76E-01
563	Lachnospiraceae_unclassified	1.07E+00	(.508928691;2.241595153)	8.62E-01	CD25+ CD56lo (log)	9.76E-01
564	Neisseria	8.65E-01	(.1684019674;4.444247277)	8.62E-01	CD69+ CD56hi (log)	9.76E-01
565	Actinobacillus	9.71E-01	(.6932741582;1.359461243)	8.63E-01	NK total (sqrt)	9.76E-01
566	Terrisporobacter	8.62E-01	(.1536962639;4.83769605)	8.66E-01	CD56hi (sqrt)	9.76E-01
567	Terrisporobacter	1.25E+00	(.0914876155;17.05589782)	8.68E-01	CD25+ CD56hi (log)	9.76E-01
568	Weeksellaceae_unclassified	1.07E+00	(.4741864346;2.42227114)	8.68E-01	CD69+ CD56lo (log)	9.76E-01
569	Rothia	9.38E-01	(.4418404136;1.992075916)	8.68E-01	CD25+ (log)	9.76E-01
570	Pasteurellaceae_unclassified	8.69E-01	(.163855114;4.612879802)	8.69E-01	CD69+ CD56lo (log)	9.76E-01
571	Prevotellaceae_unclassified	8.94E-01	(.2314880347;3.456059751)	8.72E-01	NKT CD8+ total (log)	9.76E-01
572	Unclassified	1.06E+00	(.5087758034;2.219274231)	8.72E-01	CD56hi (sqrt)	9.76E-01
573	Actinobacillus	1.11E+00	(.3149747419;3.904024323)	8.72E-01	CD25+ CD56hi (log)	9.76E-01
574	Actinomyces	1.09E+00	(.3881457606;3.050020982)	8.73E-01	CD56lo (log)	9.76E-01
575	Neisseriaceae_unclassified	1.12E+00	(.2741796356;4.550747455)	8.77E-01	NK total (log)	9.76E-01
576	Pseudomonas	1.13E+00	(.1945994885;6.563188529)	8.92E-01	NKT CD8+ total (log)	9.86E-01
577	Campylobacter	9.26E-01	(.3027081777;2.830528991)	8.92E-01	CD69+ CD56hi (log)	9.86E-01
578	Streptococcus	1.06E+00	(.4672534968;2.38964631)	8.95E-01	CD56hi (sqrt)	9.86E-01
579	Bradyrhizobium	9.26E-01	(.2907440897;2.950311586)	8.97E-01	CD56hi (sqrt)	9.87E-01
580	Oxalobacteraceae_unclassified	9.24E-01	(.2728973021;3.129663516)	8.99E-01	NK total (log)	9.88E-01
581	Oxalobacteraceae_unclassified	1.02E+00	(.7364194165;1.416460632)	8.99E-01	CD56lo (sqrt)	9.88E-01
582	Absconditabacteriales_(SR1)_ge	9.32E-01	(.2985818451;2.908767902)	9.03E-01	CD25+ (log)	9.89E-01
583	Lachnoanaerobaculum	1.10E+00	(.2362629258;5.116317273)	9.04E-01	CD56hi (sqrt)	9.89E-01
584	Unclassified	1.05E+00	(.4841933887;2.266538122)	9.06E-01	NK total (log)	9.89E-01
585	Fusobacterium	1.08E+00	(.2972654616;3.907497921)	9.09E-01	CD25+ CD56lo (log)	9.90E-01
586	Corynebacterium	9.55E-01	(.4303656177;2.119197122)	9.10E-01	CD25+ (log)	9.90E-01
587	Oribacterium	1.02E+00	(.7051104244;1.4783393)	9.12E-01	NK total (sqrt)	9.90E-01
588	Capnocytophaga	1.02E+00	(.7203697275;1.442699026)	9.13E-01	NK total (sqrt)	9.90E-01
589	Prevotellaceae_ge	1.10E+00	(.1811462972;6.660923495)	9.19E-01	CD56lo (log)	9.94E-01
590	Unclassified	9.63E-01	(.4642495333;1.997558401)	9.19E-01	CD56lo (log)	9.94E-01
591	Prevotellaceae_ge	1.11E+00	(.1462949661;8.411029295)	9.20E-01	NK total (log)	9.94E-01

592	Neisseriaceae_unclassified	1.16E+00	(.0586438623;23.11899242)	9.20E-01	CD56hi (sqrt)	9.94E-01
593	Prevotellaceae_ge	1.05E+00	(.3926108838;2.812546707)	9.21E-01	CD25+ CD56lo (log)	9.94E-01
594	Afipia	9.36E-01	(.25329055;3.461883963)	9.22E-01	CD69+ CD56lo (log)	9.94E-01
595	Oribacterium	9.52E-01	(.33130214;2.735489613)	9.27E-01	CD69+ CD56lo (log)	9.94E-01
596	Unclassified	9.91E-01	(.8170680972;1.202640873)	9.29E-01	CD56lo (sqrt)	9.94E-01
597	Xanthobacteraceae_unclassified	1.04E+00	(.3729499139;2.907370886)	9.38E-01	CD69+ (log)	1.00E+00
598	Terrisporobacter	1.04E+00	(.3535705496;3.075393029)	9.40E-01	CD25+ (log)	1.00E+00
599	Actinobacillus	1.01E+00	(.7415770996;1.381126533)	9.40E-01	CD56lo (sqrt)	1.00E+00
600	Oxalobacteraceae_unclassified	9.88E-01	(.7019234827;1.389768104)	9.43E-01	NK total (sqrt)	1.00E+00
601	Prevotella	9.88E-01	(.7066465245;1.381273536)	9.44E-01	CD56lo (sqrt)	1.00E+00
602	Lachnospiraceae_unclassified	9.87E-01	(.678294721;1.435212008)	9.44E-01	CD56lo (sqrt)	1.00E+00
603	Methylobacterium-Methylorubrum	1.05E+00	(.2542443117;4.351644445)	9.44E-01	CD25+ CD56hi (log)	1.00E+00
604	Selenomonas	9.35E-01	(.1376325306;6.352957158)	9.45E-01	CD56hi (sqrt)	1.00E+00
605	Terrisporobacter	9.49E-01	(.2120779329;4.246226374)	9.45E-01	CD69+ CD56lo (log)	1.00E+00
606	Prevotellaceae_unclassified	1.01E+00	(.7207654913;1.419564123)	9.47E-01	CD56lo (sqrt)	1.00E+00
607	Fluviicola	1.11E+00	(.0349244908;35.47745914)	9.52E-01	CD56hi (sqrt)	1.00E+00
608	Prevotella	9.69E-01	(.2994724203;3.136696491)	9.58E-01	CD56lo (log)	1.00E+00
609	Rothia	1.03E+00	(.3407999216;3.106679409)	9.60E-01	CD69+ (log)	1.00E+00
610	Streptococcus	1.01E+00	(.5682449293;1.810037934)	9.62E-01	CD56lo (log)	1.00E+00
611	Oxalobacteraceae_unclassified	9.74E-01	(.3103187599;3.056886263)	9.64E-01	CD56lo (log)	1.00E+00
612	Fluviicola	9.59E-01	(.1516012256;6.071995717)	9.65E-01	CD69+ CD56hi (log)	1.00E+00
613	Weeksellaceae_unclassified	9.82E-01	(.405173834;2.381862519)	9.69E-01	CD56lo (log)	1.01E+00
614	Actinobacillus	1.02E+00	(.3346715905;3.123097708)	9.69E-01	CD69+ CD56hi (log)	1.01E+00
615	Corynebacterium	9.73E-01	(.2483071995;3.816188335)	9.69E-01	CD56hi (sqrt)	1.01E+00
616	Neisseriaceae_unclassified	1.02E+00	(.3770181728;2.752140494)	9.71E-01	CD25+ (log)	1.01E+00
617	Neisseria	1.04E+00	(.0985732872;11.04923711)	9.72E-01	CD56hi (sqrt)	1.01E+00
618	Lautropia	1.02E+00	(.3266797203;3.189112232)	9.72E-01	CD69+ (log)	1.01E+00
619	Pseudomonas	1.04E+00	(.1026037567;10.55727144)	9.73E-01	CD56hi (sqrt)	1.01E+00
620	Actinobacillus	1.02E+00	(.2814475508;3.71162176)	9.74E-01	NK total (log)	1.01E+00
621	Oribacterium	1.02E+00	(.308053961;3.368009889)	9.76E-01	CD56lo (log)	1.01E+00
622	Fusobacterium	1.02E+00	(.3227106212;3.208748777)	9.76E-01	CD25+ (log)	1.01E+00
623	Prevotella	1.02E+00	(.2256618365;4.632175147)	9.77E-01	CD69+ CD56hi (log)	1.01E+00
624	Rothia	1.01E+00	(.365703191;2.80599127)	9.80E-01	NKT CD8+ total (log)	1.01E+00
625	Sphingomonas	1.01E+00	(.4772146549;2.134861691)	9.81E-01	CD25+ (log)	1.01E+00
626	Streptococcus	9.98E-01	(.8529282383;1.168077958)	9.81E-01	CD56lo (sqrt)	1.01E+00
627	Selenomonas	9.81E-01	(.1750983643;5.490909628)	9.82E-01	CD56hi (sqrt)	1.01E+00
628	Oribacterium	9.97E-01	(.6958081593;1.427592524)	9.85E-01	CD56lo (sqrt)	1.01E+00
629	Weeksellaceae_unclassified	1.01E+00	(.4410908418;2.300789791)	9.86E-01	CD69+ (log)	1.01E+00
630	Atopobium	1.03E+00	(.0589484616;17.84099451)	9.86E-01	CD56hi (sqrt)	1.01E+00
631	Neisseria	1.01E+00	(.4966612751;2.0373971)	9.87E-01	CD25+ CD56lo (log)	1.01E+00
632	Prevotellaceae_unclassified	9.97E-01	(.6987757337;1.423610107)	9.89E-01	NK total (sqrt)	1.01E+00
633	Terrisporobacter	1.01E+00	(.1408812905;7.305698339)	9.89E-01	CD56lo (log)	1.01E+00
634	Campylobacter	9.93E-01	(.2900434583;3.400012437)	9.91E-01	CD56hi (sqrt)	1.01E+00
635	Rothia	1.00E+00	(.3340133575;3.021851954)	9.93E-01	CD69+ CD56lo (log)	1.01E+00
636	Planococcaceae_unclassified	9.97E-01	(.4241915055;2.3440137)	9.95E-01	CD69+ CD56hi (log)	1.01E+00
637	Terrisporobacter	1.00E+00	(.1297026507;7.705245331)	1.00E+00	NK total (log)	1.01E+00

OR=odds ratio; 95%-CI=95% confidence interval

p-values from fractional regressions adjusting for group variable, age, bmi, and sex
